# Integrated *in silico* and *in vitro* exploration of the anti-VEGFR-2 activities of a semisynthetic xanthine alkaloid inhibiting breast cancer

**DOI:** 10.1371/journal.pone.0316146

**Published:** 2025-01-27

**Authors:** Eslam B. Elkaeed, Hazem Elkady, Ahmed M. Khattab, Reda G. Yousef, Hanan A. Al-ghulikah, Dalal Z. Husein, Ibrahim M. Ibrahim, Mohamed A. Elkady, Ahmed M. Metwaly, Ibrahim H. Eissa

**Affiliations:** 1 Department of Pharmaceutical Sciences, College of Pharmacy, AlMaarefa University, Riyadh, Saudi Arabia; 2 Pharmaceutical Medicinal Chemistry & Drug Design Department, Faculty of Pharmacy (Boys), Al-Azhar University, Cairo, Egypt; 3 Pharmaceutical Chemistry Department, Faculty of Pharmacy, Merit University, Sohag, Egypt; 4 Department of Chemistry, College of Science, Princess Nourah Bint Abdulrahman University, Riyadh, Saudi Arabia; 5 Chemistry Department, Faculty of Science, New Valley University, El-Kharja, Egypt; 6 Biophysics Department, Faculty of Science, Cairo University, Cairo, Egypt; 7 Biochemistry & Molecular Biology Department, Faculty of Pharmacy (Boys), Al-Azhar University, Cairo, Egypt; 8 Pharmacognosy and Medicinal Plants Department, Faculty of Pharmacy (Boys), Al-Azhar University, Cairo, Egypt; University of Helsinki, FINLAND

## Abstract

This study presents **T-1-NBAB**, a new compound derived from the natural xanthine alkaloid theobromine, aimed at inhibiting VEGFR-2, a crucial protein in angiogenesis. **T-1-NBAB**’s potential to interacts with and inhibit the VEGFR-2 was indicated using *in silico* techniques like molecular docking, MD simulations, MM-GBSA, PLIP, essential dynamics, and bi-dimensional projection experiments. DFT experiments was utilized also to study the structural and electrostatic properties of **T-1-NBAB**. Computational analysis was performed to predict the ADME-Tox profiles of **T-1-NBAB**. After semisynthesis, the *in vitro* results showed that **T-1-NBAB** effectively inhibits VEGFR-2, with an IC_50_ of 0.115 μM, compared to sorafenib’s 0.0591 μM. *In vitro* tests also demonstrated significant activity of **T-1-NBAB** against breast cancer cell lines MCF7 and T47D, with IC_50_ values of 16.88 μM and 61.17 μM, respectively, and high selectivity. Importantly, **T-1-NBAB** induced early and late apoptosis in MCF7 cells, indicating its potential as a strong anticancer agent. Additionally, **T-1-NBAB** reduced the migration and healing abilities of MCF7 cells, suggesting it could be a promising anti-angiogenic agent. Overall, these findings suggest that **T-1-NBAB** is a promising lead compound for further research as a potential treatment for breast cancer.

## 1. Introduction

Cancer remains a leading cause of death worldwide. Traditional nonsurgical treatments like chemotherapy and radiation have long faced challenges such as low survival rates, high morbidity, recurrence, and poor prognosis. For example, in 2020, the incidence of breast cancer exceeded 2.26 million cases [1]. Advancements in understanding human neoplastic diseases and technology allow for the development of new antineoplastic drugs to decrease cancer-related fatalities [2].

Metastasis is an involved set of steps. Cancer cells need to move from the original tumor, enter the lymphatic system or bloodstream, avoid detection by the immune system, stay alive in the bloodstream, and grow in distant organs [3, 4]. A crucial component of cancer progression and development is the growth of new blood vessels from established vasculatures [5, 6].

One kind of transmembrane receptor is Vascular endothelial growth factor receptor 2 (VEGFR-2), which is mostly located on the surface of endothelial cells [7]. These cells are responsible for lining the inside of blood vessels. Because it is overexpressed in numerous tumor forms, VEGFR-2 is of special importance in the setting of cancer [8]. As a result, a viable approach to therapeutic cancer therapy is the creation of inhibitors to block the VEFG/VEGFR-2 signaling pathway [9].

Metastasis is the main cause for the poor prognosis in breast cancer in which tumor angiogenesis plays critical roles [10–12]. It was reported that VEGFR-2 is up-regulated in invasive primary and metastatic breast cancers [13]. Also, VEGFR-2 expression correlates positively with lymph node metastasis of breast cancer. Patients with high expression of VEGFR-2 had a significantly worse Overall Survival [14]. It was found that VEGFR-2 inhibitors slowed the growth rate of primary tumors and reduced blood vessel density, neither agent was able to prevent lymphatic metastasis when given after tumor cells had seeded the lymph node. Anti-angiogenic therapies, mostly targeting the VEGF/VEGFR signaling axis, are currently routinely used in the clinic to treat several advanced or metastatic cancers, including colon, kidney, liver and breast cancers [15, 16].

Research on small molecule VEGFR-2 inhibitors is gaining significant attention in the search for new anticancer medications. Several powerful VEGFR-2 inhibitors have been created and have shown therapeutic effectiveness in treating cancer patients [17].

Regrettably, the FDA-approved VEGFR-2 inhibitors have been linked to several adverse effects, including hypertension [18], proteinuria, hemorrhage and/or bleeding [19], hypothyroidism, fistula, bowel perforation, left ventricular diastolic dysfunction, thrombotic microangiopathy, reversible posterior leukoencephalopathy syndrome and arterial thrombosis [18, 20], proteinuria [21], germline polymorphisms [22, 23]. So that, the discovery of new less toxic VEGFR-2 inhibitors in an urgent need.

Throughout history, nature has consistently provided mankind with essential resources such as food, medicine, and cosmetics, serving as a dependable source of sustenance and healing [24, 25]. The search for natural anti-cancer drugs has led researchers to explore xanthine derivatives, known for their antimutagenic effects against several cancers, including ovarian cancer [26, 27], and glioblastoma multiforme [28, 29]. Our laboratory has identified numerous potential anticancer candidates that exhibit VEGFR-2-inhibitory activity. These candidates come from various classes and derivatives, including nicotinamide [30–32], xanthines [33–37], thiazolidine [38, 39], naphthalene [40], pyridine [41], quinoline [42] indole [40], and isatin [43].

### 1.1. Rationale

VEGFR-2 inhibitors work by blocking the ATP binding site of the VEGFR-2 protein [44, 45]. To fit properly into the active site of VEGFR-2, these inhibitors have four key pharmacophoric features [46–48]. These features include a hetero aromatic system buried in the hinge region of the receptor and can form important hydrogen bonds (H_Bs) with Cys917 [49, 50]. Another feature is a spacer moiety to occupy the gate keeper region [51]. The spacer moiety gives the ideal length for the ligand to facilitate the third feature (pharmacophore) to occupy the region of the DFG motif that has two important amino acids (Glu883 and Asp1044) to be anchored be the pharmacophore moiety. The reported pharmacophore moieties are urea, thiourea, amide, and diamides [52]. The fourth feature is the hydrophobic tail which occupies the allosteric pocket of the ATP binding site [53].

The structures of toceranib **I** and sunitinib **II**, FDA-approved anti-VEGFR-2 drugs, have isatin moiety as a heterocyclic head. In addition, these drugs have amide groups as pharmacophore moieties. On the other hand, the structures of sorafenib **III** and cabozantinib **IV**, FDA-approved anti-VEGFR-2 drugs, have a substituted phenyl ring as a hydrophobic tail. Furthermore, our team has discovered compound **V** as a VEGFR-2 inhibitor and apoptosis inducer with an IC_50_ value of 2.7 nM. The such compound has *N*-phenylacetamide moiety as a spacer group [54].

**Fig 1:**
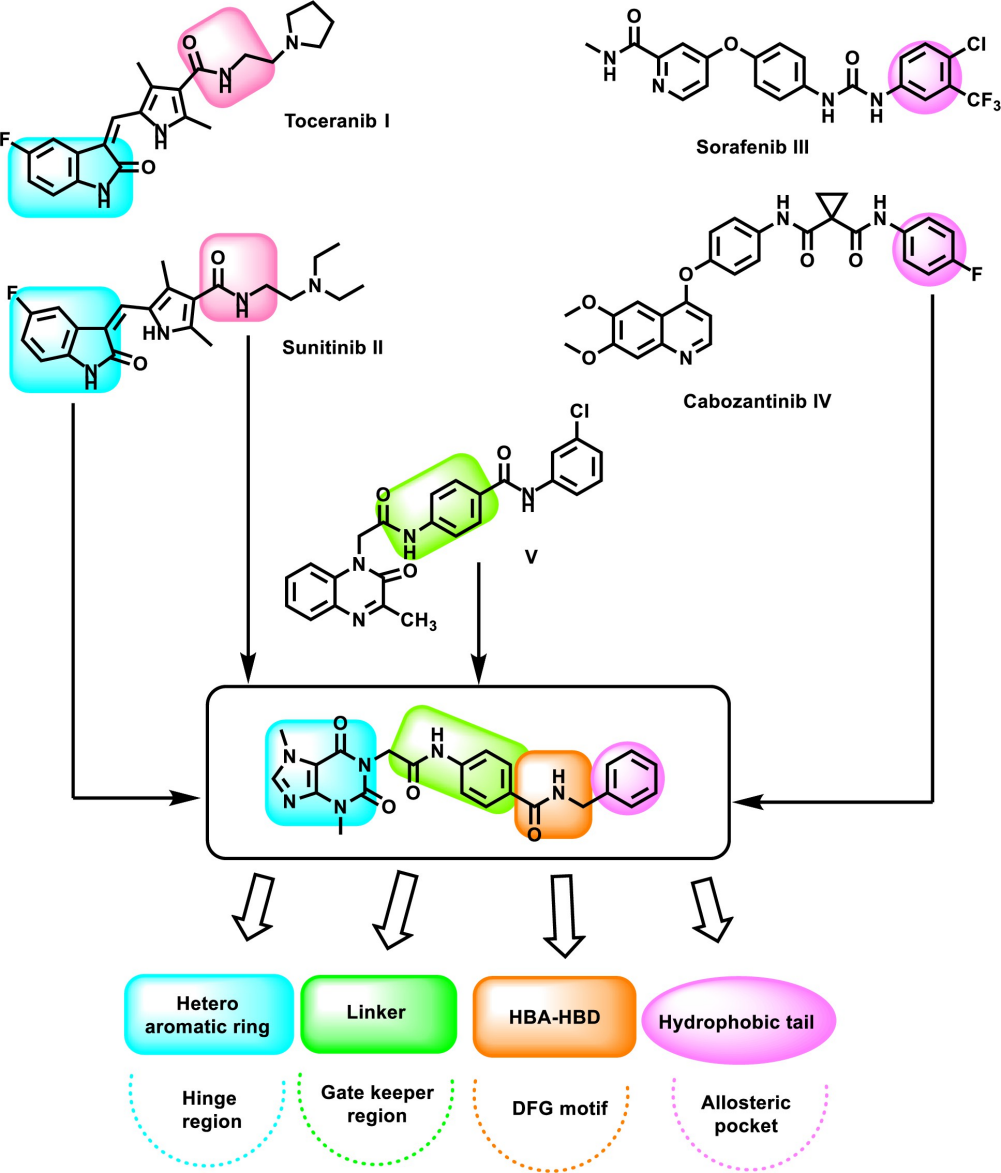
Rational design of T-1-NBAB.

In this work, we depended on a ligand-based drug design approach to design a new VEGFR-2 inhibitor (**T-1-NBAB**) have essential pharmacophoric features (**Fig 1**). We followed the principles of the Computer Assisted Drug Design (CADD) method as outlined by Eissa *et al*. (2023) [55], employing the xanthine moiety as a start compound. The xanthine moiety, ring equivalent of isatin moieties of **I** and **II**, were employed as heterocyclic-head to target the hinge region. In addition, the amide moiety of **I** and **II** was also incorporated in the designed compound as a pharmacophore moiety to target the DFG motif region. Furthermore, the linker **(***N*-phenylacetamide) moiety of compound **V** was used as a linker in the designed molecule targeting the gate keeper region. Finally, we used the benzyl moiety, the ring equivalent of substituted phenyl rings of the moiety of compounds **III** and **IV**, as a hydrophobic tail targeting the allosteric pocket.

In this work, the xanthine moiety was utilized as a heterocyclic head based on some pharmacodynamic features. The xanthine derivatives were reported to target many regulatory enzymes leading to phosphodiesterase inhibition, adenosine antagonizing activity, activation of histone deacetylase, etc [56]. Various natural and synthetic xanthine derivatives have been recognized as therapeutically potent compounds and reported for targeting various diseases as respiratory diseases [57], blood pressure [58], renal disease [59], inflammation [60], infection [61], and tumor [62]. In addition, xanthine derivatives antioxidant activities [63].

## 2. Results and discussions

### 2.1. Computational studies

#### 2.1.1. Molecular docking

**Fig 2:**
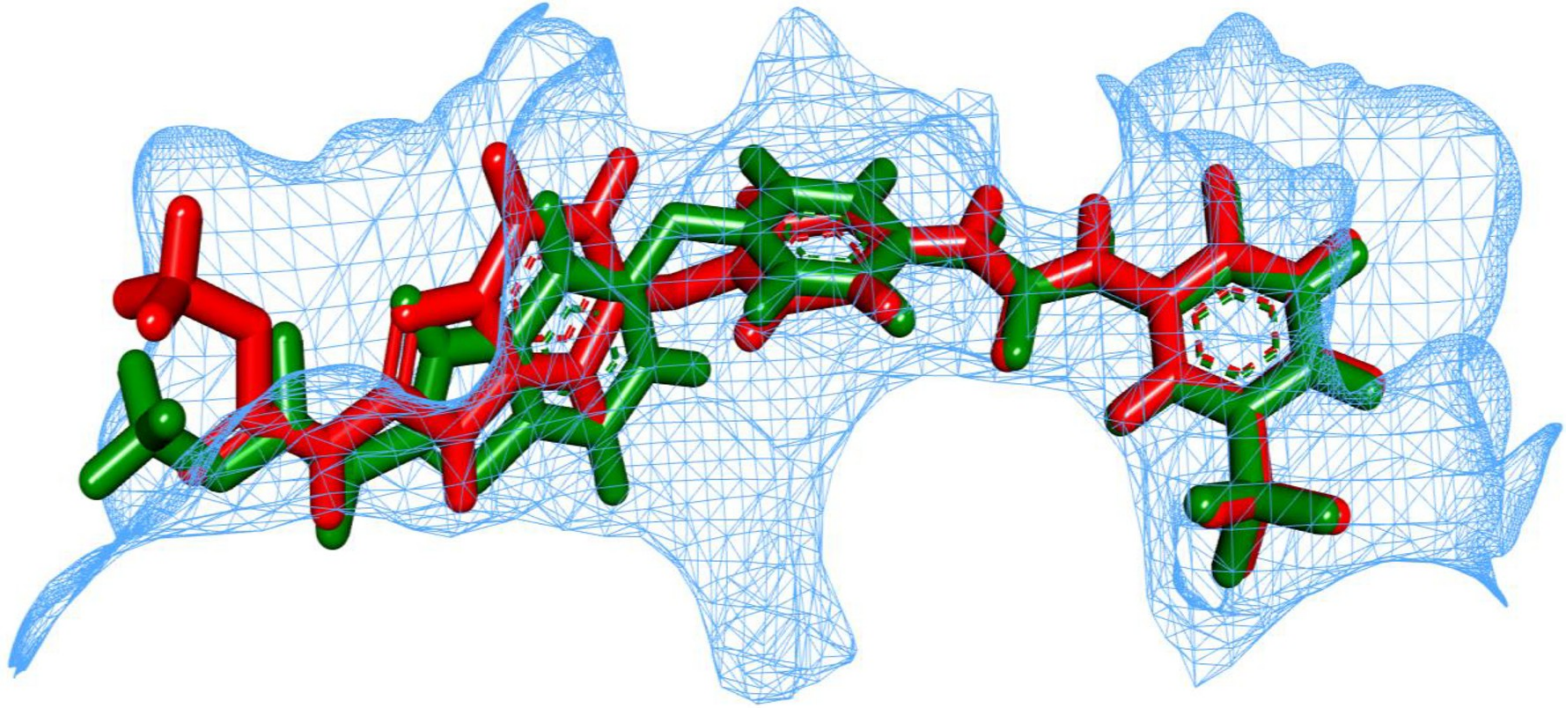
Validation of docking procedure inside VEGFR-2; native pose (orange) and the outputted pose (green).

The prediction of a molecule’s capability to bind to a given protein target is made feasible by a molecular docking tool [64]. This work used the MOE 2019 protocol to conduct docking studies of **T-1-NBAB** against the target enzyme, VEGFR-2 (pdb code: 2OH4). First, the native ligand (sorafenib) was docked into VEGFR-2 active site and produced a small RMSD value which supported the validity of the docking process **(Fig 2)**.

**Fig 3:**
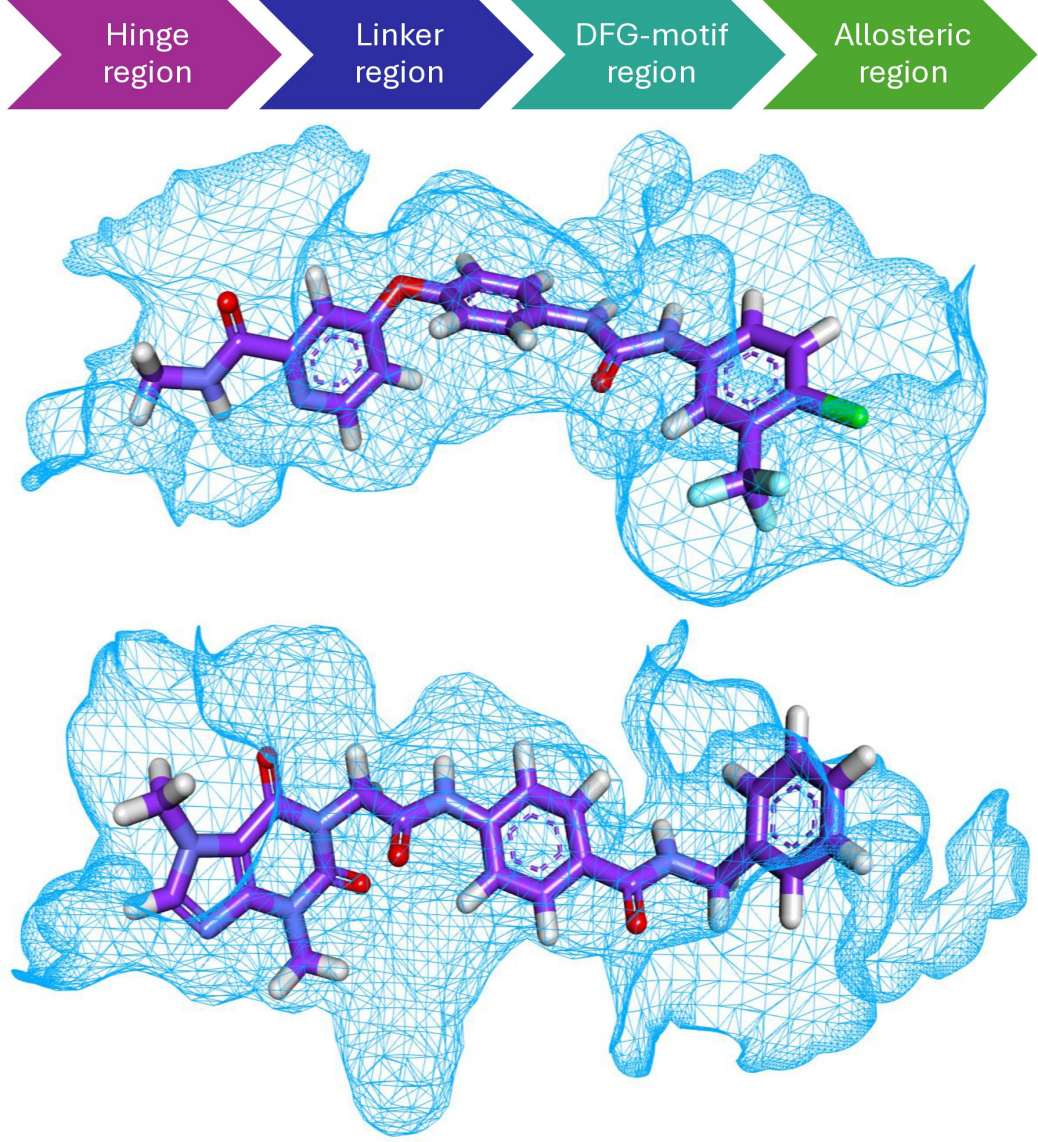
Mapping surfaces of sorafenib and T-1-NBAB inside the VEGFR-2’s active site.

Mapping surface images showed that sorafenib and T-1-NBAB occupied the VEGFR-2’s active site as presented in **Fig 3.**

**Fig 4:**
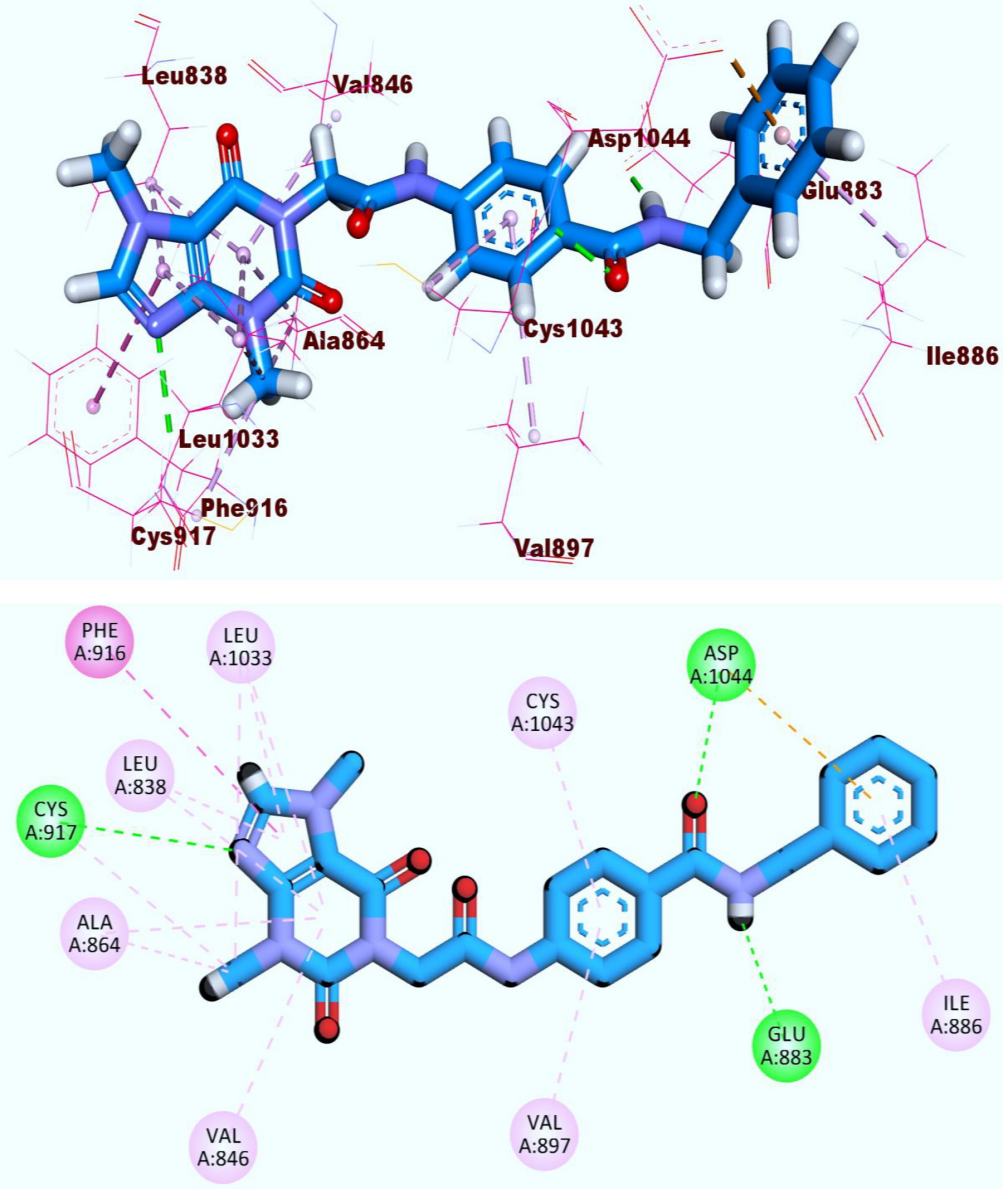
3D and 2D binding of T-1-NBAB against the VEGFR-2’s active pocket. The hydrogen bonds are represented in green dashed lines and the hydrophobic interactions are represented pink dashed lines.

The 3d and 2d depictions, **Fig 4**, described **T-1-NBAB’s** binding inside the VEGFR-2’s active site main regions. The purine fragment of **T-1-NBAB** could occupy the hinge region that was composed of Cys917, Leu1033, Ala864, Leu838, and Phe916 residues where one key H_B with Cys917 and eleven hydrophobic interactions (H_Is) were observed in this region. As well, the central phenyl moiety formed two pi-pi interactions with the amino acids Val897 and Cys1043. The amide group that was close to benzyl moiety formed two H_Bs with the essential amino acid residues, Glu883 and Asp1044, in the DFG motif region. **T-1-NBAB** fits precisely into the allostric pocket and interacts with the hydrophobic back bone (Ile886) by its terminal benzyl head. These results indicate and predict a potent hindering potential of **T-1-NBAB** against the VEGFR-2.

#### 2.1.2. Molecular dynamic (MD) simulation

**Fig 5:**
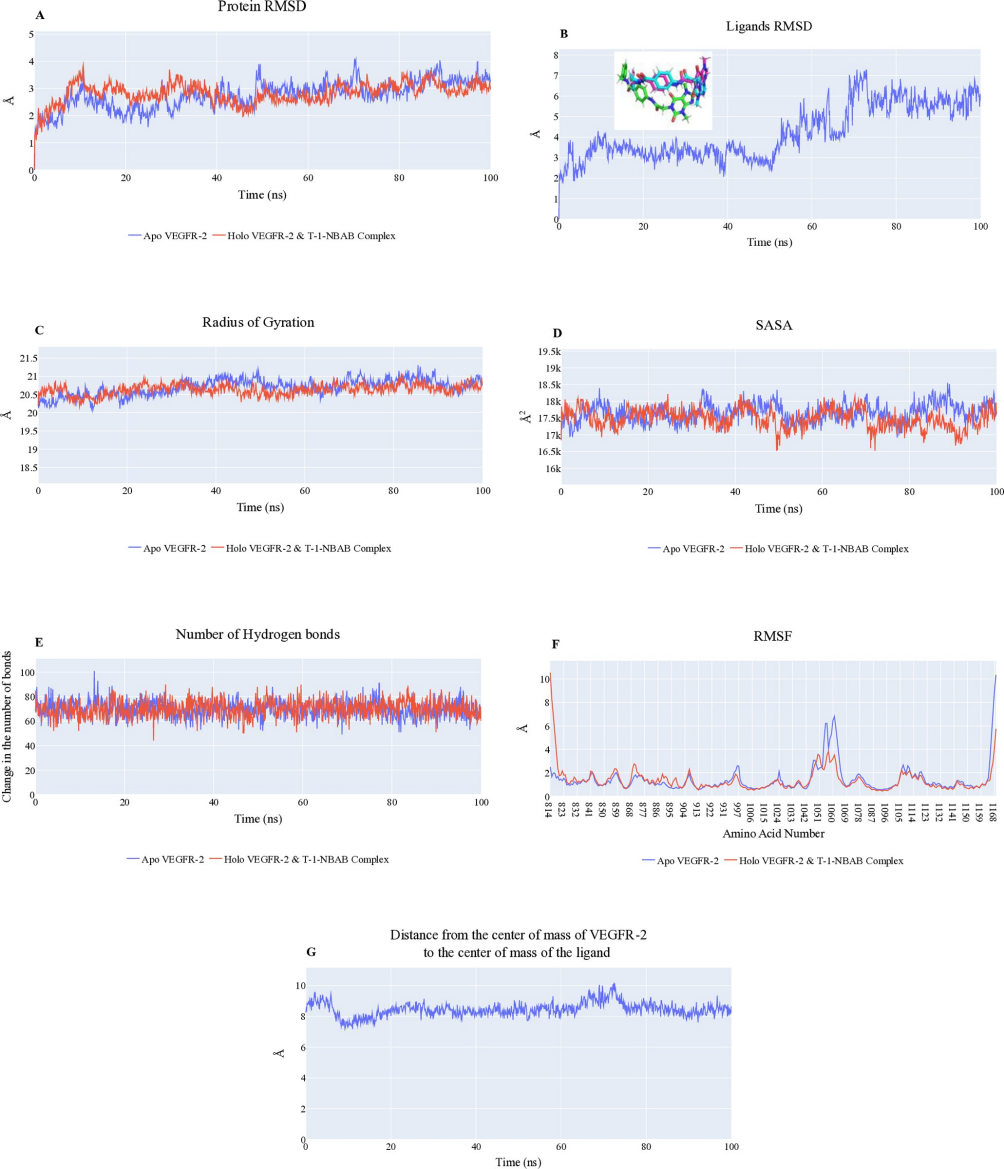
A) RMSD values of VEGFR-2, B) RMSD values of **T-1-NBAB,** with an inset showing **T-1-NBAB** at various times: green (12.1 ns), cyan (74.2 ns), and magenta (93.8 ns), C) radius of gyration, D) SASA, E) Changes in VEGFR-2’s hydrogen bonds number, F) RMSF, G) Distance from the mass center between **T-1-NBAB** and the VEGFR-2.

Throughout the production run, the two systems (**T-1-NBAB**-apo (unbound)VEGFR-2 and **T-1-NBAB**-holo (bound) VEGFR-2) were studied, and it was found that **T-1-NBAB** showed two different conformations within the binding pocket, always maintaining a stable average distance from the VEGFR-2’s center of mass. **Fig 5A** (blue and red lines) demonstrate that the RMSD plots of apo and holo VEGFR-2 remain stable at an average value of 2.7 Å after around 10 ns. However, the RMSD of **T-1-NBAB** exhibits two conformations over the simulated time window. **T-1-NBAB** shows an average of 3.15 Å in the first 50 ns, while the second state has a higher average value of 5.28 Å. The inset of **Fig 5B** shows what has prompted this sharp increase. The **T-1-NBAB** is shown in green at 12.1 ns, in cyan at 74.2 ns, and in magenta at 93.8 ns exhibiting a motion inside the binding pocket. **Fig 5C** illustrates a consistent pattern in the radius of gyration (RoG), with an average measurement of 20.5 Å across **T-1-NBAB**-holo VEGFR-2 and **T-1-NBAB**-apo VEGFR-2 complexes. This suggests a similar degree of compactness in VEGFER-2’s structures. **Fig 5D** presents the solvent-accessible surface area (SASA) values, indicating that the proteins, both in **T-1-NBAB**-apo VEGFR-2 and **T-1-NBAB**-holo VEGFR-2 complexes, maintain average SASA values of 17,656 Å^2^ and 17,491 Å^2^, respectively. This data reflects the exposed surface area available to the solvent in each protein state, showing a slight reduction in the holo form. **Fig 5E** shows a continuous fluctuation in the number of hydrogen bonds, with both systems averaging around 70 H-bonds. This consistency suggests similar stability and intermolecular interactions within the VEGFR-2 structure. The RMSF plot (**Fig 5F**) indicates a very minor variation across the amino acids (less than 2 Å), except for the holo protein’s N-terminal, and Ala1048:Ala1063 loop showed variation levels of 10.5, and 3.8 Å, respectively. Also, in the apo system, the Tyr994:Asp996 loop (2.5 Å), the Gly1046:Leu1065 loop (6.6 Å), and the Pro1106:Ile1112 loop (2.6 Å). Additionally, the C-terminal (10.3 Å for the apoVEGFR-2 protein and 5.7 Å for the holo VEGFR-2 protein). In conclusion, **T-1-NBAB** maintains a consistent distance from its center of mass to the VEGFR-2 center of mass having an average spacing of 8.4 Å (**Fig 5G**).

#### 2.1.3. MM-GBSA studies

**Fig 6:**
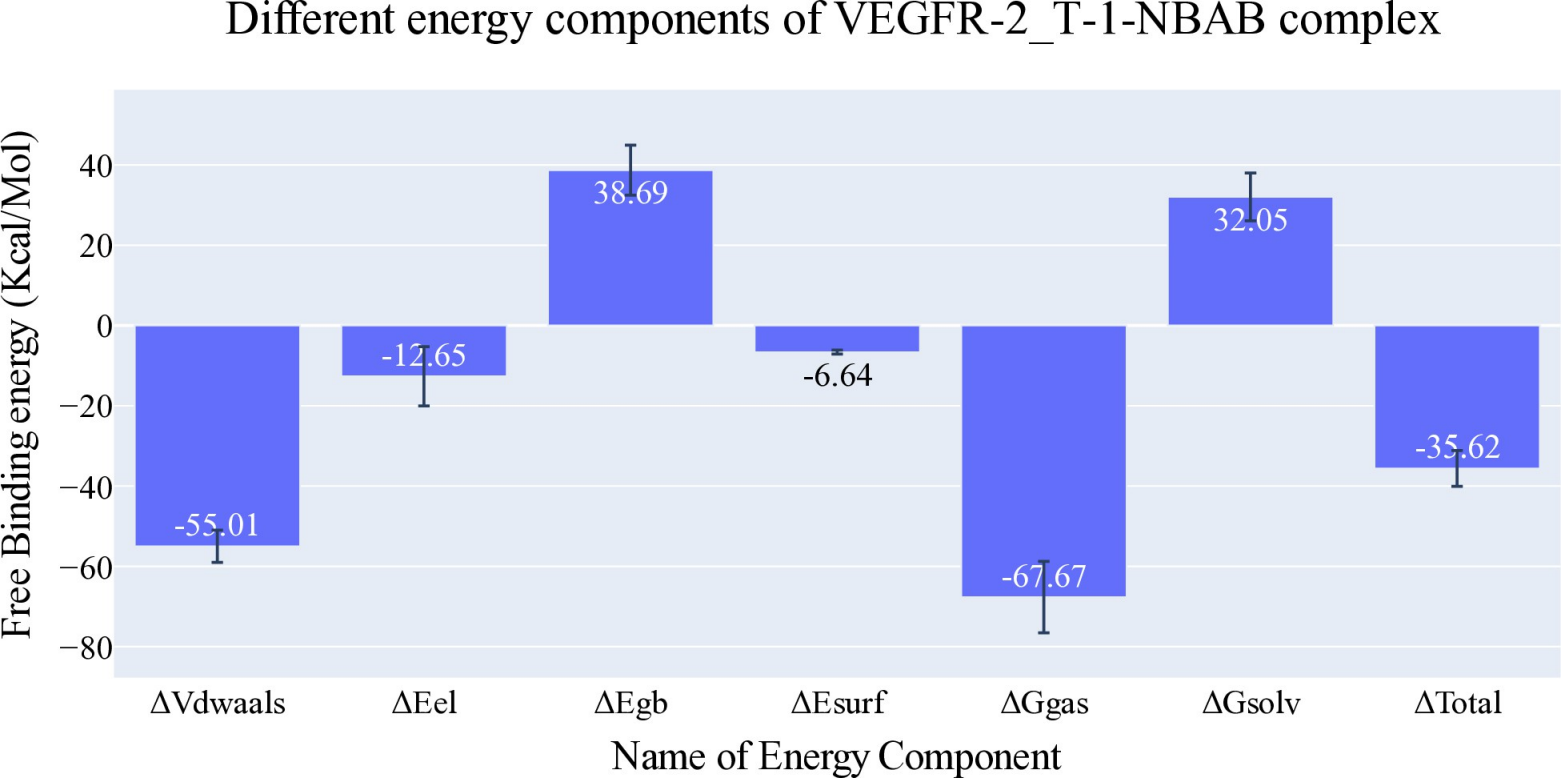
MM-GBSA analysis values. Bars denote the standard deviations.

**Fig 7:**
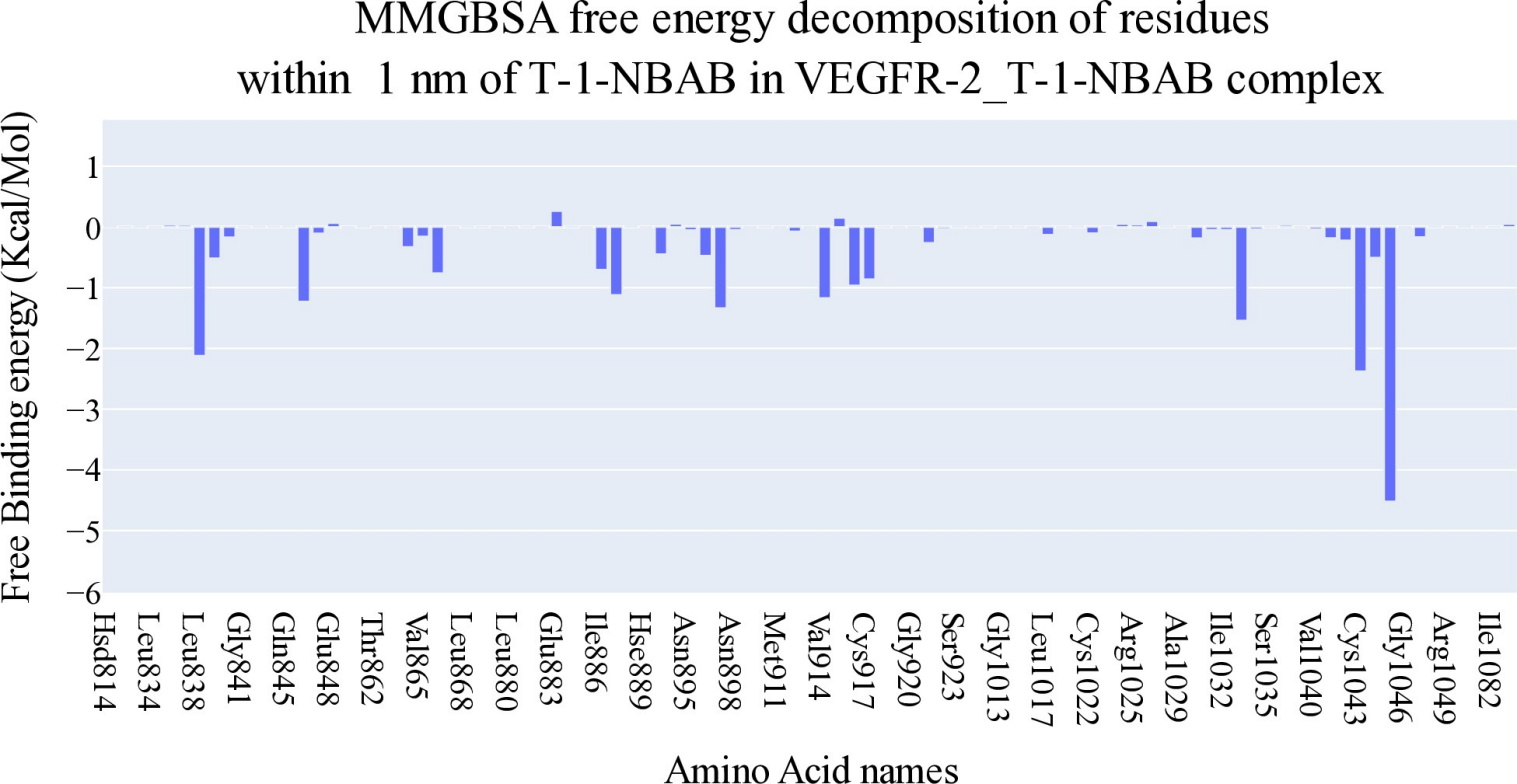
VEGFR-2-T-1-NBAB’s binding free energy decomposition.

**Fig 6** depicts the various components contributing to the MM-GBSA binding free energy analysis. For the **T-1-NBAB**, the average overall binding energy is -35.62 kcal/mol. Among the different energy components, the electrostatic interaction is the least favorable, averaging around -12.65 kcal/mol. In contrast, the van der Waals interactions provide the most favorable contribution, with an average energy of approximately -55 kcal/mol. These results highlight the dominant role of van der Waals forces in the binding affinity of **T-1-NBAB**, while electrostatic interactions contribute less favorably. Additionally, a decomposition analysis, shown in Fig 7, identified amino acids within 1 nm of **T-1-NBAB** that contribute to the binding with values less than -1 kcal/mol. The identified amino acids are Leu838 (-2.12 Kcal/Mol), Val846 (-1.22 Kcal/Mol), Leu887 (-1.11 Kcal/Mol), Val897 (-1.33 Kcal/Mol), Val914 (-1.16 Kcal/Mol), Leu1033 (-1.54 Kcal/Mol), Cys1043 (-2.37 Kcal/Mol), and Phe1045 (-4.51 Kcal/Mol).

#### 2.1.4. PLIP studies

**Fig 8:**
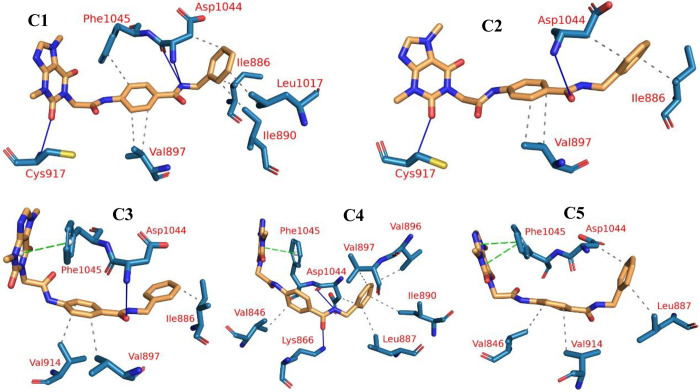
Different interactions of VEGFR-2-T-1-NBAB’s clusters. H-bond: Blue solid, Pi-stacking: green dashed, hydrophobic interaction: dashed grey, amino acids: blue, and **T-1-NBAB**: orange.

We then clustered the trajectories of the VEGFR-2-**T-1-NBAB** complex and generated a representative frame for each cluster. Using the elbow method, we determined that there were five clusters. For each cluster representative, we identified the interaction counts and types between VEGFR-2 and **T-1-NBAB** using the PLIP webserver. Table 1 shows the number and type of PLIP interactions. There was a significant variation in the total number of interactions reported, with 24 hydrophobic interactions, 8 hydrogen bonds, and 4 Pi-stacking interactions. One amino acid occurs more often than any other in each type of interaction. In the first four clusters, Asp1044 establishes a stable H-bond, whereas Val897 demonstrates a stable hydrophobic contact. One amino acid, Phe1045, establishes the Pi-stacking interaction starting from the third cluster representative at 56.2 ns. Besides the producing the interaction numbers and types from PLIP, the experiment generates a.pse file to examine the 3D conformation of the VEGFR-2-**T-1-NBAB**’s interactions (**Fig 8**).

**Table 1:** PLIP interactions calculations. Bold amino acids represent those with the highest number of interactions.

Cluster number	No. of H- bonds	Amino acids	Number of pi-stacking	Amino acids	No. of hydrophobic interactions	Amino acids
**C1**	3	Cys917—**Asp1044** (2)	0	None	7	Ile886—Ile890—**Val897** (2)—Leu1017—Asp1044—Phe1045
**C2**	2	Cys917—**Asp1044**	0	None	4	Ile886—**Val897** (2)—Asp1044
**C3**	1	**Asp1044**	1	**Phe1045**	3	Ile886—**Val897**—Val914
**C4**	2	Lys866—**Asp1044**	1	**Phe1045**	6	Val846—Leu887—Ile890—Val896—**Val897**—Asp1044
**C5**	0	None	2	**Phe1045** (2)	4	Val846—Leu887—Val914—Asp1044

#### 2.1.5. Essential dynamic studies

**Fig 9:**
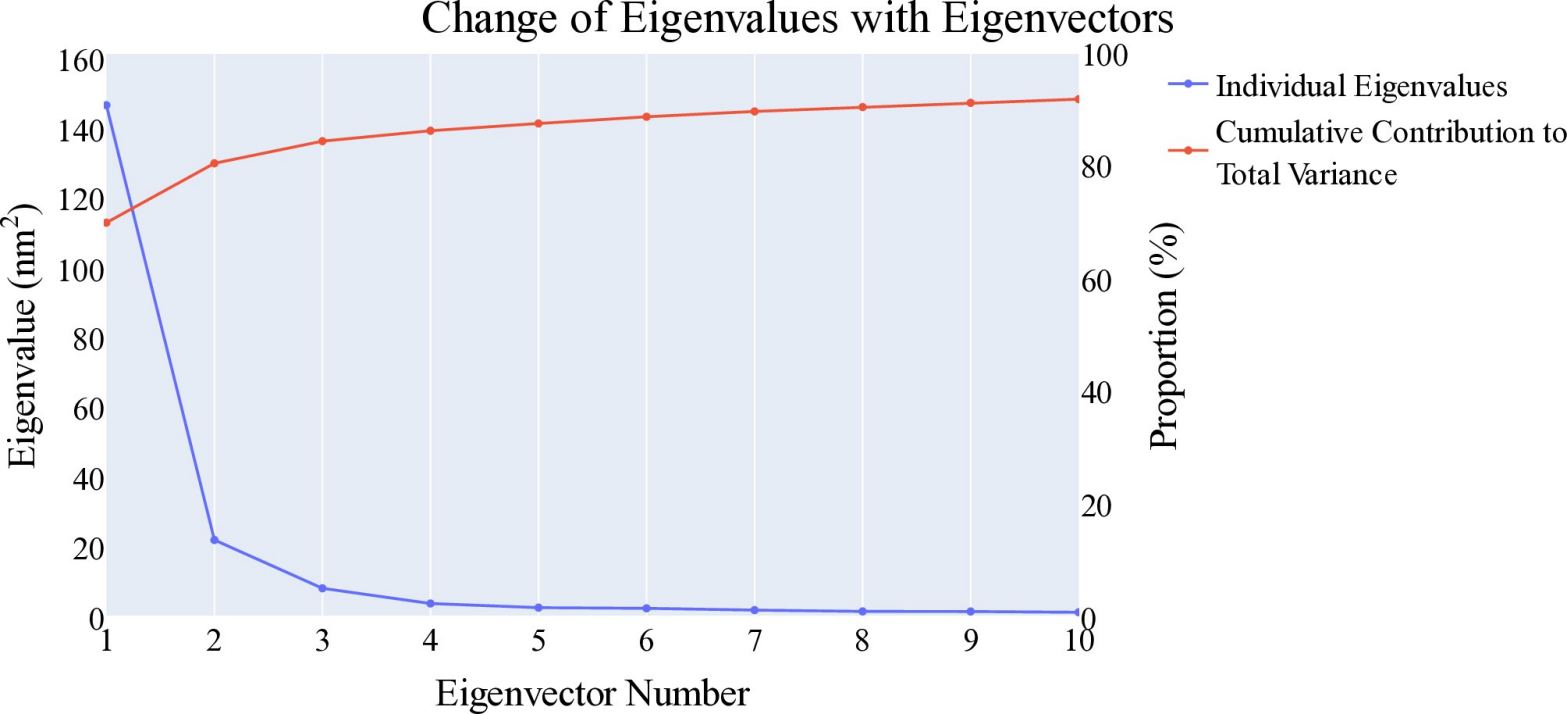
Eigenvalues changes.

**Fig 10:**
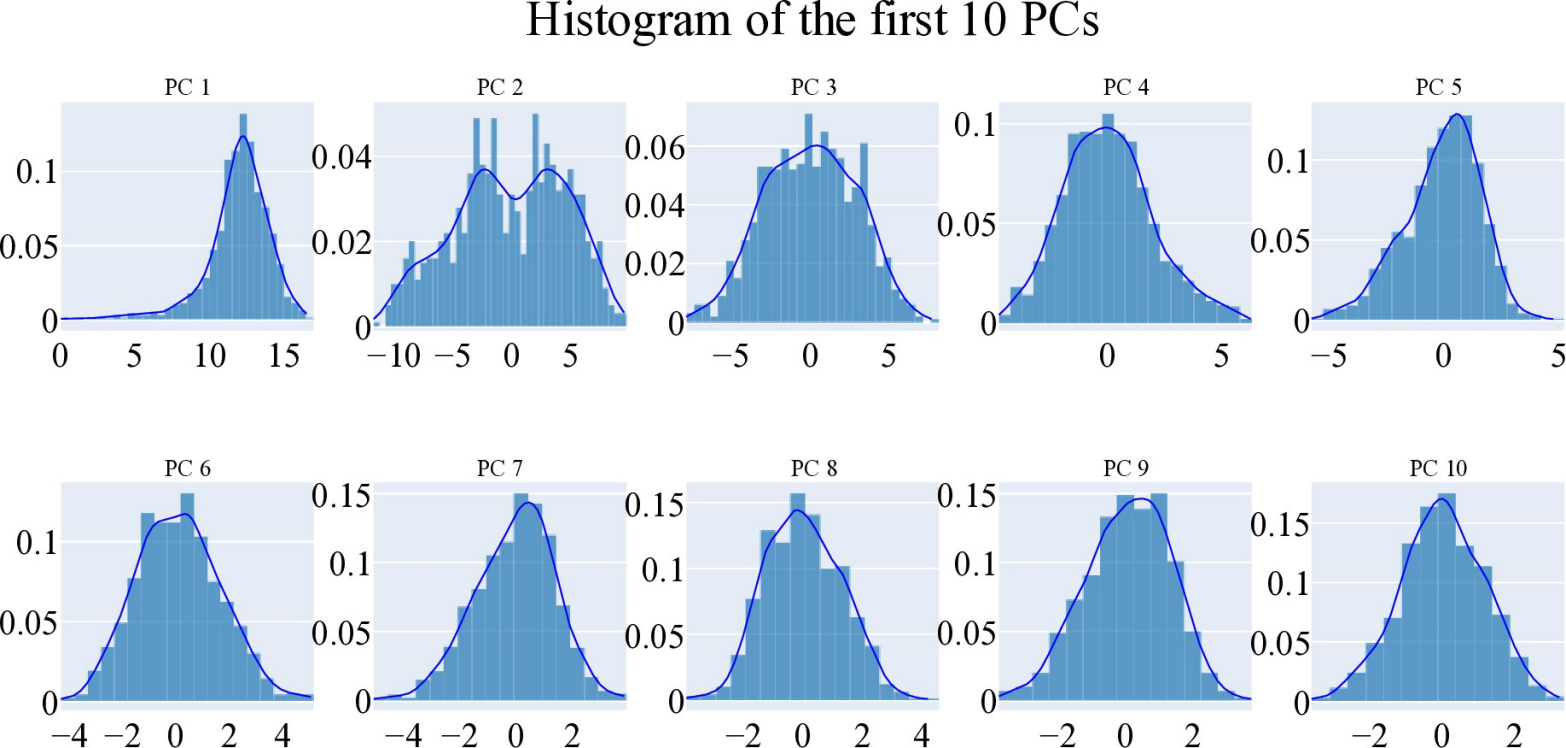
First ten eigenvectors’ distribution.

**Fig 11:**
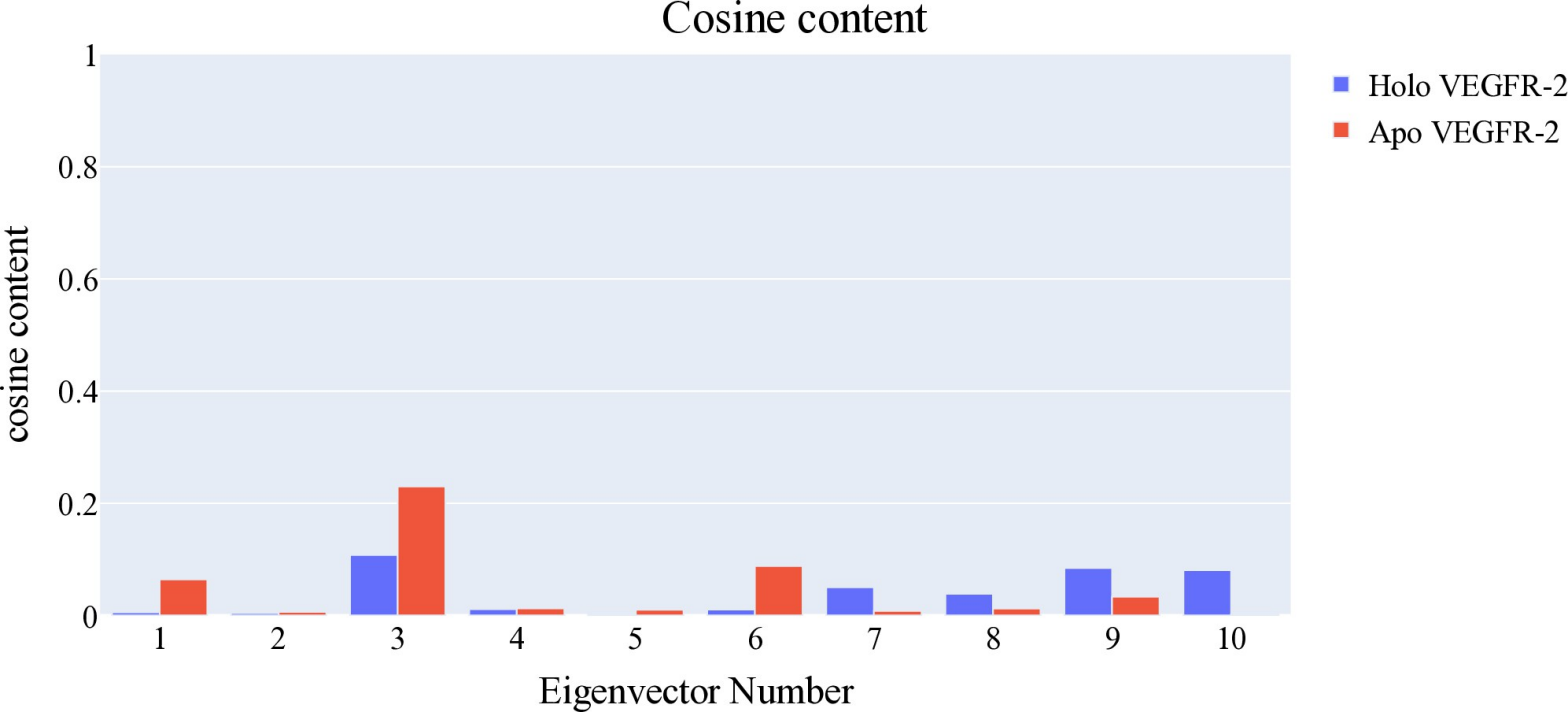
Cosine content values for the trajectories of T-1-NBAB- apo-VEGFR-2 (red) and T-1-NBAB- holo-VEGFR-2 (blue) complexes.

Principal component analysis was performed to identify the sources of coordinated movement within the trajectories of **T-1-NBAB**- apo-VEGFR-2 and **T-1-NBAB**- holo-VEGFR-2 complexes. The essential subspace was determined using a scree plot, the distribution of eigenvectors, and the variance explained by additional eigenvectors. The scree plot showed a marked decrease in the slope after the second principal component, indicating the top three eigenvectors accounted for approximately 84.5% of the total variance, with the first eigenvector alone capturing nearly 70% (**Fig 9**). The first three eigenvectors displayed a non-Gaussian distribution (**Fig 10**), making them representative of the essential subspace. To evaluate the randomness of motion, the cosine content was calculated for the first ten eigenvectors in both simulations of **T-1-NBAB**- apo-VEGFR-2 and **T-1-NBAB**- holo-VEGFR-2 complexes. The cosine content for these eigenvectors was below 0.25 in both protein states (**Fig 11**). The limited overlap between the first three eigenvectors (25.5% according to Root Mean Square Inner Product analysis) indicated distinct sampling in the trajectories of **T-1-NBAB**- apo-VEGFR-2 and **T-1-NBAB**- holo-VEGFR-2 complexes. Furthermore, RMSIP analysis showed that the similarity between the apo and holo C matrices was only 36.3%.

#### 2.1.6. Bidimensional projections assessments

**Fig 12:**
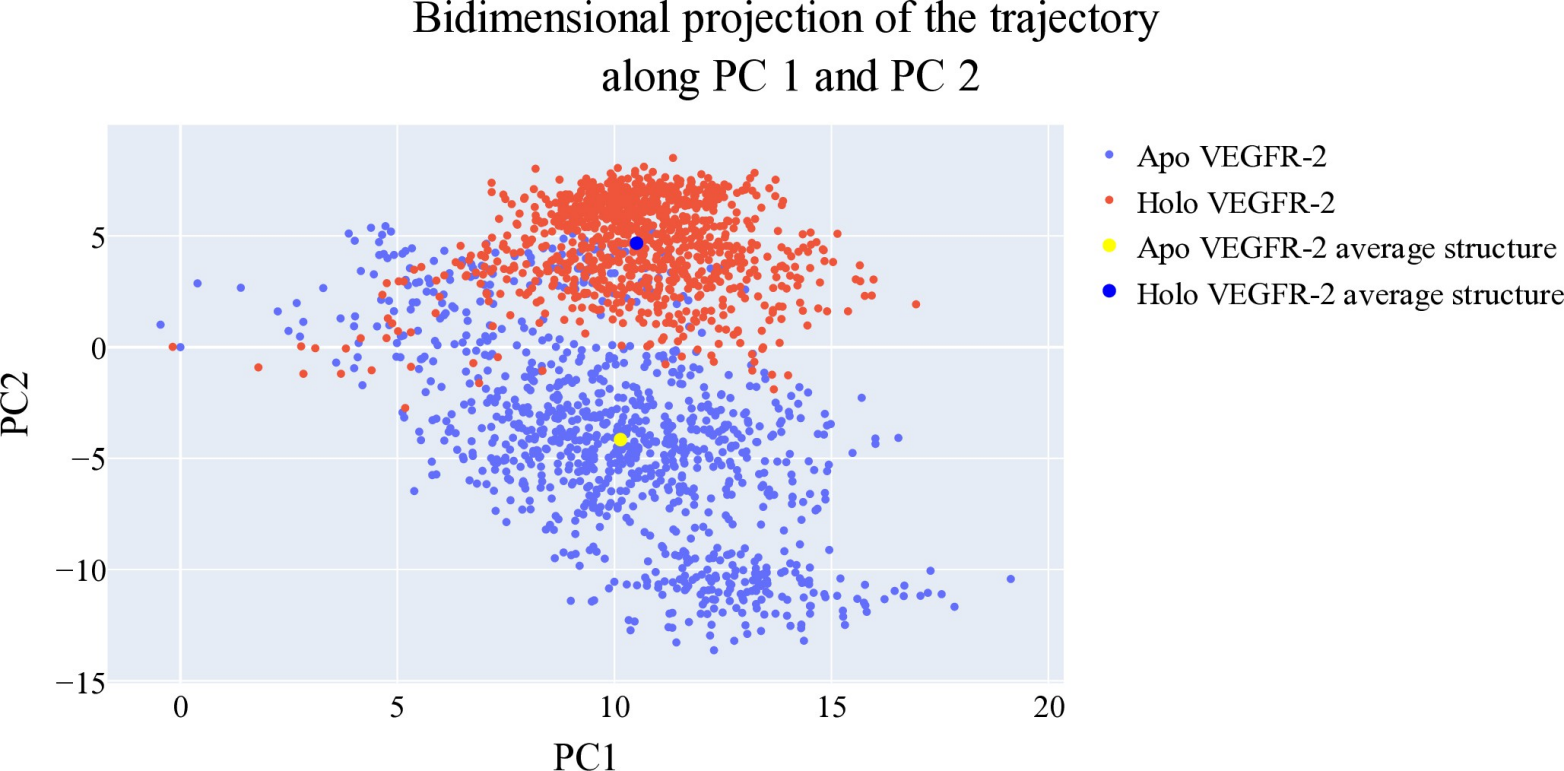
1^st^ two eigenvectors’ projection.

**Fig 13:**
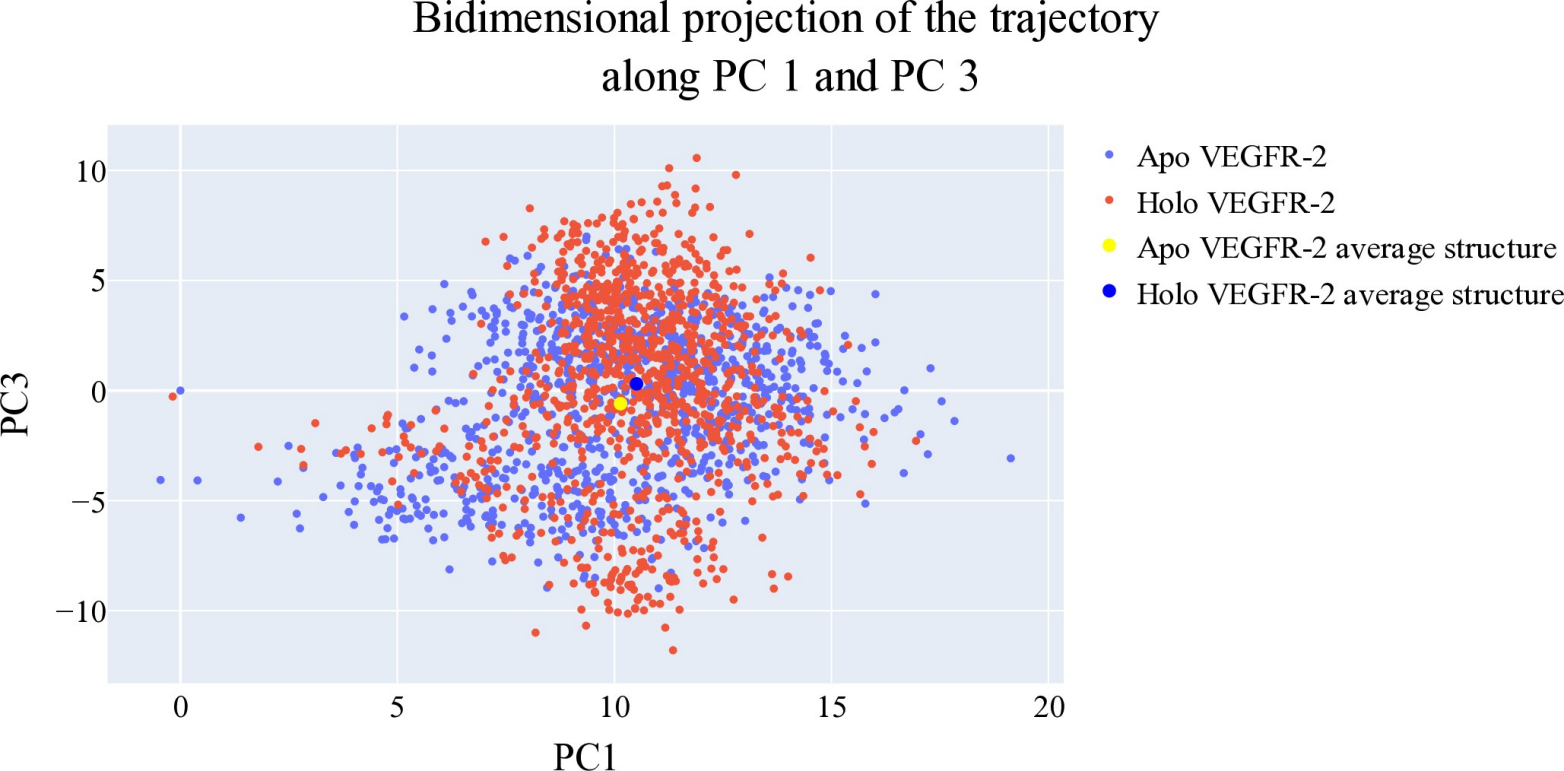
1^st^ and 3^rd^ eigenvectors’ projection.

**Fig 14:**
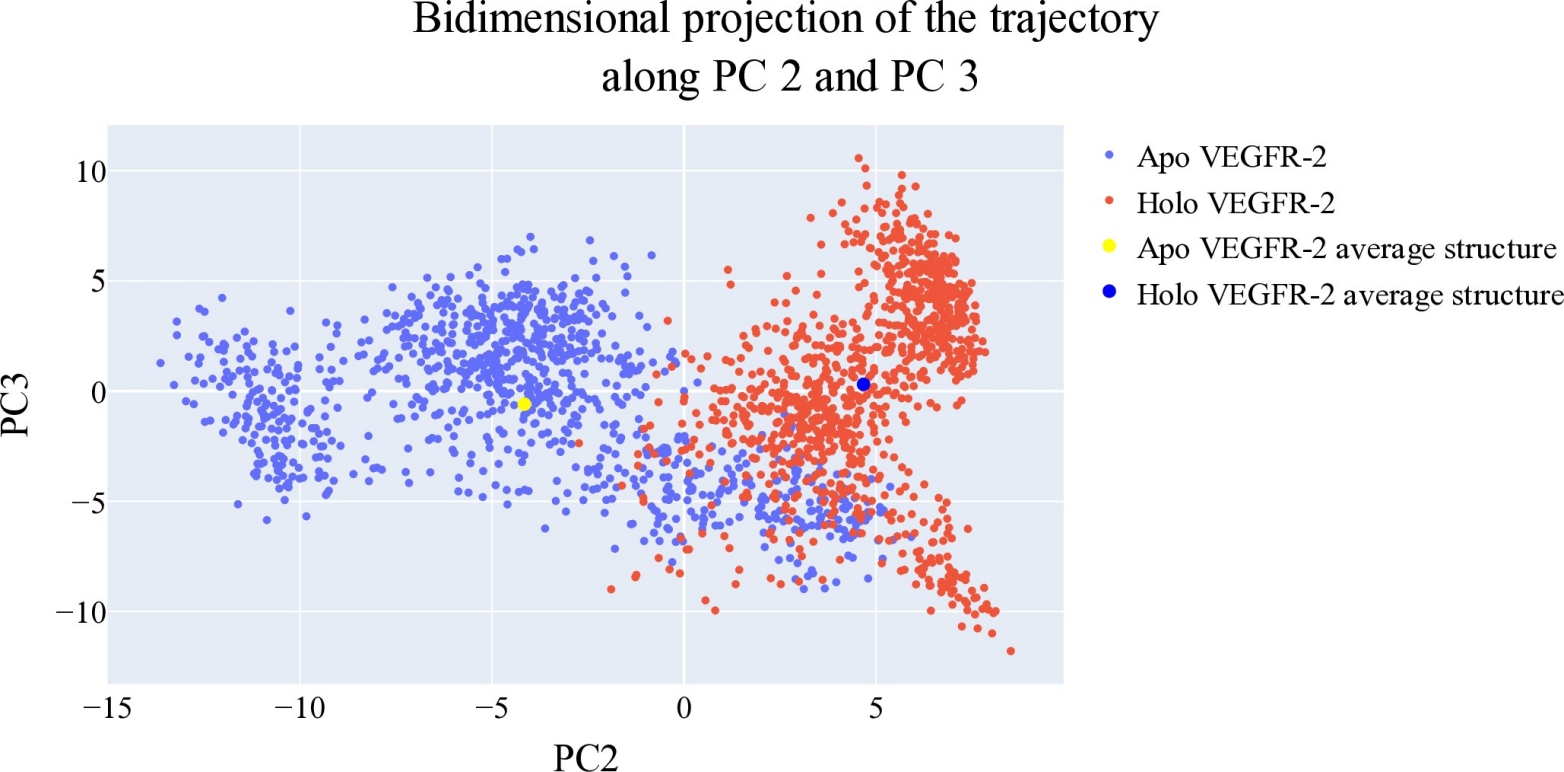
2^nd^ and 3^rd^ eigenvectors projection.

The projections of each trajectory onto the first three eigenvectors of the combined C matrix are displayed in **Figs 12–14**. In these plots, a large dot represents the average structure of each trajectory. **Fig 12**, showing the projection onto the first two eigenvectors, reveals distinct average structures with minimal overlap between the trajectories of **T-1-NBAB**- apo*-*VEGFR-2 (blue) and **T-1-NBAB**- holo-VEGFR-2 (red) complexes. In **Fig 13**, the projection onto the first and third eigenvectors indicates that the two average structures are quite similar, with significant overlap in their trajectories. **Fig 14**, depicting the projection onto the second and third eigenvectors, shows little overlap between the trajectories of **T-1-NBAB**- apo*-*VEGFR-2 (blue) and **T-1-NBAB**- holo-VEGFR-2 (red) complexes, with their average structures appearing different.

**Fig 15:**
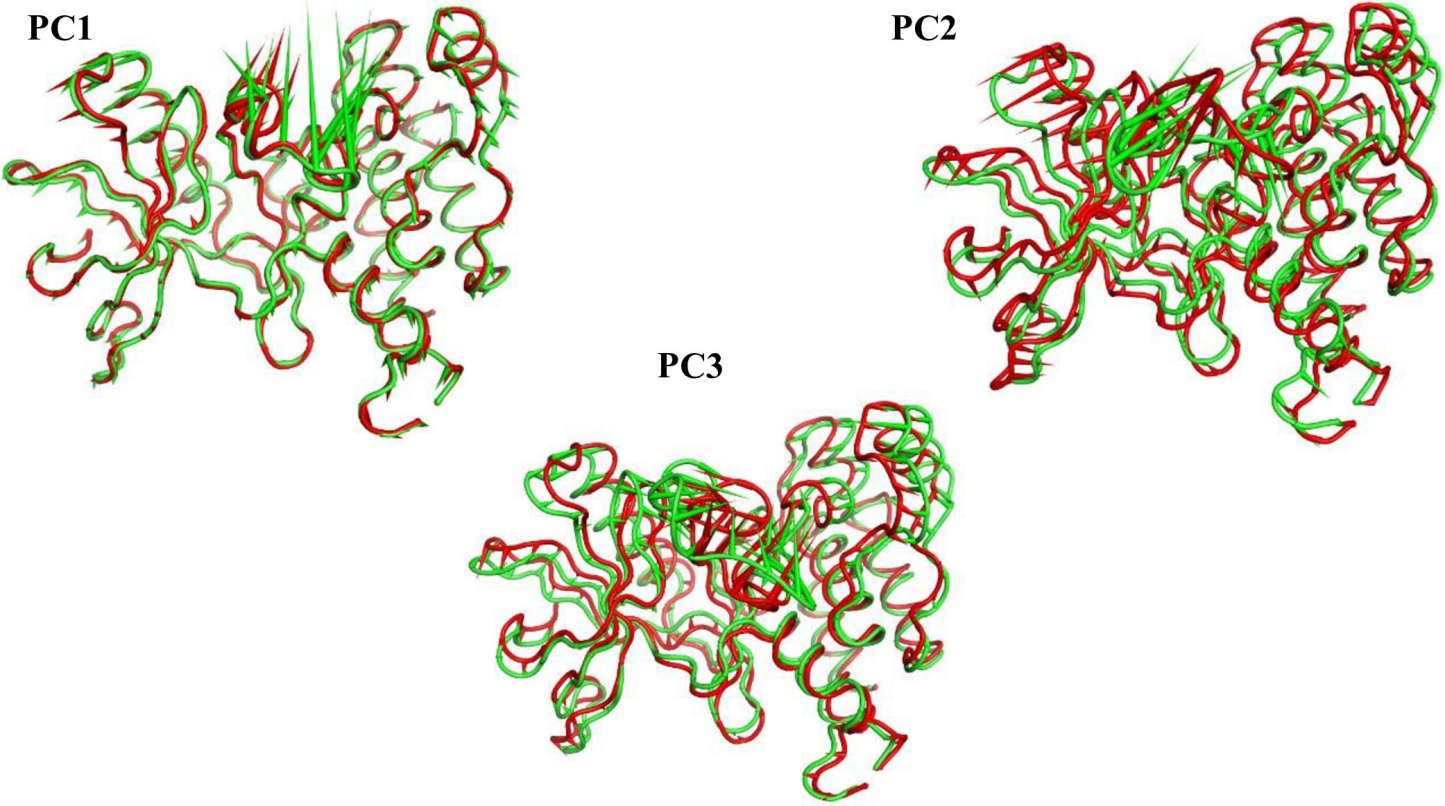
Porcupine representation of the 1^st^ three eigenvectors for T-1-NBAB- apo-VEGFR-2 (green) and T-1-NBAB- holo-VEGFR-2 (red) complexes.

To illustrate the motions captured by the first three eigenvectors, porcupine diagrams are shown in **Fig 15**. The most notable motion identified in these eigenvectors is the movement of the Gly1046 loop. The first eigenvector characterizes the loop’s opening motion in both trajectories, though the extent of this motion differs. The second eigenvector reveals that the loop opens in the apo protein but closes in the holo structure, indicating opposing motions in the two systems. The third eigenvector shows similar loop-opening motion in both trajectories.

#### 2.1.7. Geometry, electronic structure and topology analysis

**Fig 16:**
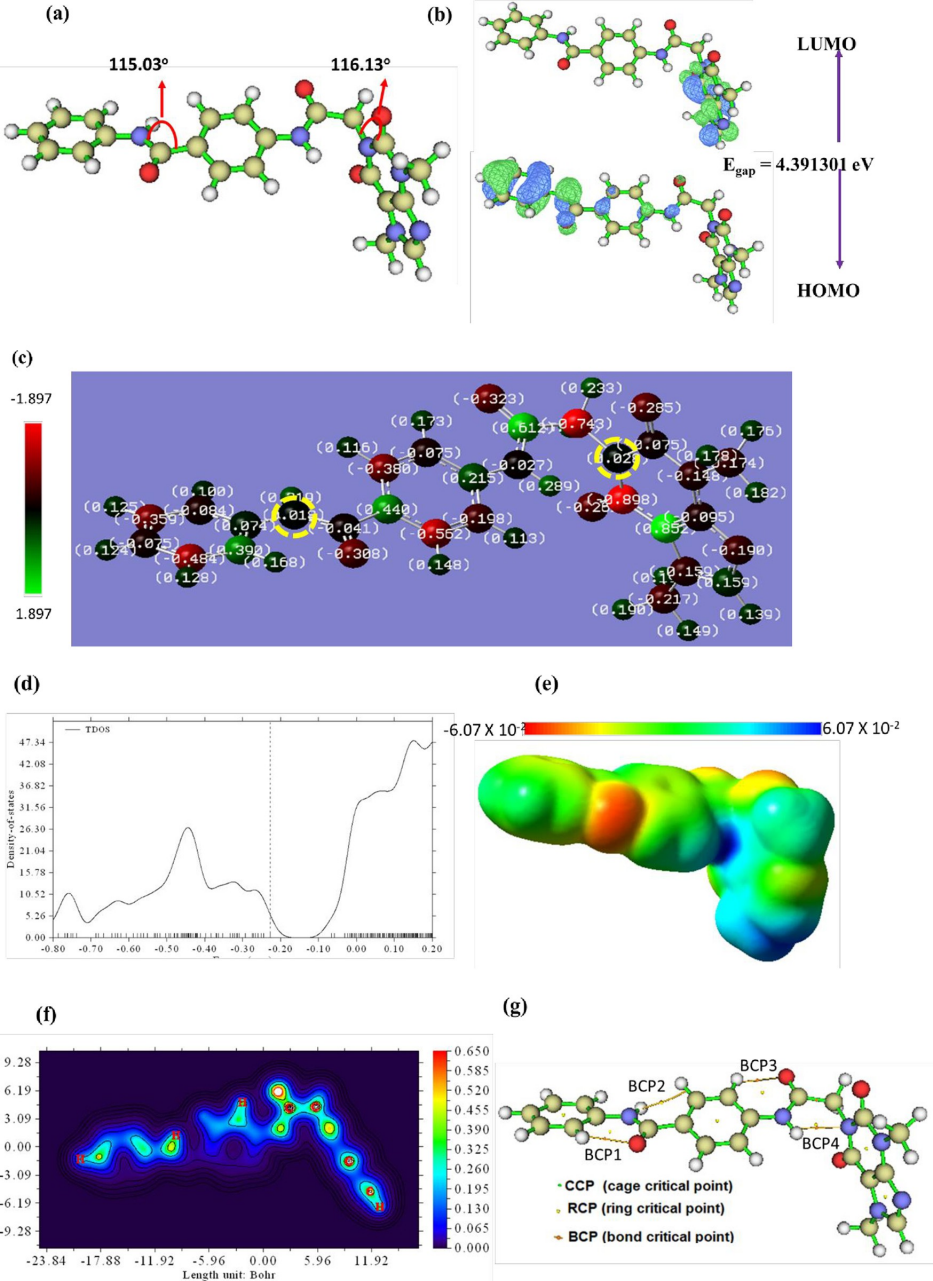
A) **T-1-NBAB**’s optimized structure geometry, B) **T-1-NBAB**’s Distribution of Mullikan charge, C) **T-1-NBAB**’s FMO electron density distribution, D) **T-1-NBAB**’s TDOS, E and F) **T-1-NBAB**’s QTAIM maps and G) **T-1-NBAB**’s ESP, at B3LYB/6-311+G(d,p).

Using the DFT/B3LYP/6-311G+(d, p) chemistry model, the structure of **T-1-NBAB** has been optimized, as illustrated in **Fig 16A**. It is clear that the dimethyl purine dione part of **T-1-NBAB** is nearly perpendicular to the rest of the structure, and the calculated dipole moment is 7.4 Debye, **Table 2**, which reflects good charge separation within the chemical structure. The wavefunctions of LUMO are located over the dimethyl purine dione part while the HOMO wavefunctions are located over the rest of the molecule. The calculated HOMO-LUMO energy (E_gap_) clearly depicted that chemical charge transfer could occur within the molecule easily. The E_gap_ value is found to be 4.39 e.V, **Fig 16B** [65]. **Fig 16C** shows the Mulliken charge dispersion along the geometry of the **T-1-NBAB**. All oxygen atoms and nitrogen atoms except for the two nitrogen that labeled in the dashed yellow circle have a negative charge. According to the color scale, the purine ring included the largest positive and negative charges in the geometry which help in the process of charge transfer. The plot of the total density of state (TDOS) in **Fig 16D** indicates the orbitals with the highest density. For **T-1-NBAB**, the highest electron density is localized over unoccupied orbitals after LUMO.

**Table 2:** T-1-NBAB’s DFT parameters.

IP	EA	μ (eV)	χ (eV)	η (eV)	σ (eV)	ω (eV)	Dm (Debye)	TE (eV)	ΔN_max_	ΔE (eV)
-6.176	-1.785	-3.980	3.980	2.196	0.455	17.394	7.405	-40280.6	1.813	-17.394

The molecule’s global reactivity indices of **T-1-NBAB** were determined, as shown in **Table 1**. Further proof of the prepared structure’s reactivity is provided by the magnitude of the softness (**σ**), electronegativity (χ) and electrophilicity (ω), suggesting the softness and the strong probability of electronic charge transfer inside the geometry of the **T-1-NBAB** [66]. The MESP map that resulted from the analysis of the Molecular Electrostatic Potential (MESP) surface is depicted in **Fig 16G**. The reddish negative MESP areas are concentrated above oxygen. These areas have an abundance of electrons, making them primed for an electrophilic reaction. The positive MESP surface (blue zones), which needs electrons, is primed to be attacked by a nucleophile. The blue zones are concentrated around hydrogen atoms that participate in H_Bs with targets as donors. The oxygen atoms serve as acceptors for H_Bs. The neutral green and yellow regions interact hydrophobically with amino acids. **T-1-NBAB** ’s potential to interact with the protein target was validated by the differential charge distribution.

The interaction inside the molecule is shown by bond routes and bond critical points, according to the quantum theory of atoms in molecules (QTAIM) studies, **Fig 16E** and **16G**. The filled colored contour mapping of **T-1-NBAB** as shown in **Fig 16E** indicates that the dimethyl purine dione moiety is semi-perpendicular to the remainder of the chemical system. **S.1 Table** and **S.1 Fig** in the **S1 File** provide detailed information on all produced bond critical points (BCPs), cage critical points (CCPs), and bond paths. **Fig 16F** illustrates four newly formed BCPs with positive electron density function values (ρ) less than 0.1 a.u. Additionally, Laplacian values (∇2ρ) were positive, indicating a non-covalent (close-shell) bonding. The electrostatic nature of these new bond paths is suggested by the positive values for total energy density (H(r)). The new BCPs formed four CCPs, which enhance geometric stability.

#### 2.1.8. ADMET profile assessment

**Fig 17:**
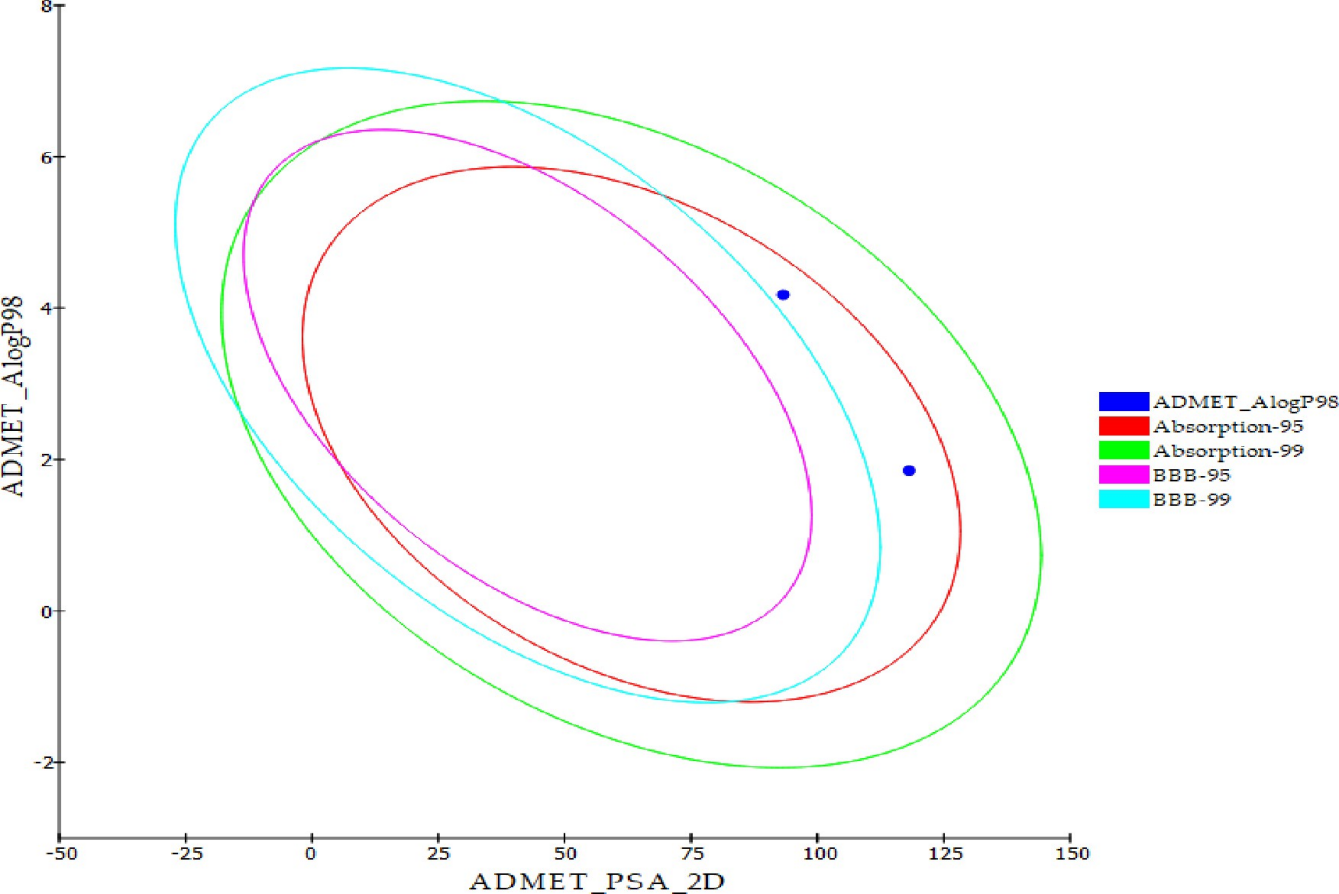
Calculated ADMET profiles for T-1-NBAB and sorafenib. ADMET_AlogP98: lipid-water partition coefficient; ADMET_PSA_2D: polar molecular surface area. Two-dimensional polar surface area (PSA_2D) each drug is plotted against their computed atom-type partition coefficient (ALogP98). The area encompassed by the ellipse represents good absorption without any violation of the ADMET properties. Based on Egan *et al*. [67] absorption model the 95% and 99% confidence limit ellipses corresponding to the blood–brain barrier (BBB) and intestinal absorption models are indicated.

Approval of a new medication requires investigation into its pharmacokinetic characteristics and biological activity. Early examination of a lead compound’s pharmacokinetic features is critical in the drug discovery process to avoid approval delays and potential late-stage retractions. To determine the ADMET parameters for **T-1-NBAB** compared to sorafenib, *in silico* ADMET parameters from Discovery Studio 4.0 were used. The ADMET results indicated that **T-1-NBAB** had a better profile than sorafenib (**Fig 17** and **Table 3**). Both compounds showed poor ability to cross the blood-brain barrier (BBB), good intestinal absorption (I-A) values, and were unlikely to inhibit CYP2D6. Additionally, **T-1-NBAB** showed better level of solubility in aqueous media (A-S) and had an ability to bind to the plasma protein (PPB) with a level of less than 90%. Interestingly, **T-1-NBAB** demonstrated an expected hepatosafety, whereas sorafenib was anticipated to be hepatotoxic.

**Table 3:** T-1-NBAB and Sorafenib ADMET parameters.

Comp.	BBB	A-S	I-A	Hepatotoxicity	CYP2D6	PPB
**T-1-NBAB**	Very low	Good	Good	No predicted toxicity	No inhibition	< 90%
Sorafenib		Low		Toxicity predicted		> 90%

#### 2.1.9. *In silico* toxicity studies

Ensuring that a new drug is effective and safe for human is critical to minimize disapproval [68]. *In vivo* and *in vitro* toxicity examination methods can be, time-consuming, expensive and have several ethically restrictions. Therefore, researchers have turned to *in silico* approached in the toxicity prediction [69]. In this study, the toxicity profile of **T-1-NBAB** was predicted utilizing eight different models included mutagenicity, carcinogenicity, and toxicity in rodent models, along with evaluations of oral and ocular irritability. The following table summarizes the findings for these parameters (**Table 4**) in Discovery Studio and comparing sorafenib.

**Table 4:** The predicted toxicity of T-1-NBAB and sorafenib.

Model	T-1-NBAB	Sorafenib
Ames mutagenicity	Non-Mutagenic	
FDA Rodent (Male-rats) Carcinogenicity	Non-Carcinogenic	
Carcinogenic (Mouse) Potency TD_50_	14.244 mg/kg /day	39.771 mg/kg /day
Maximum Tolerated (Rats) Feeding Dose	0.026 g/kg	0.082 g/kg
Oral LD_50_ (Rats)	7.252 g/kg	0.823 g/kg
Chronic LOAEL (Rats)	0.038 g/kg	0.005 g/kg
Ocular irritability	Mild	
Topical irritability	No irritation	

The comparative *in silico* toxicity analysis of **T-1-NBAB** and sorafenib reveals several noteworthy advantages of **T-1-NBAB** in terms of safety and efficacy.

Regarding mutagenicity and carcinogenicity, both **T-1-NBAB** and sorafenib were found to be non-mutagenic in the Ames test and non-carcinogenic in male rats, which is a positive indicator of **T-1-NBAB**’s genetic safety. This similarity suggests that **T-1-NBAB** has a low likelihood of causing mutations or cancer in treated organisms, which is essential for long-term therapeutic use. On the other hand, **T-1-NBAB** demonstrated a significantly lower carcinogenic potency in mice with a TD_50_ of 14.244 mg/kg/day compared to sorafenib’s 39.771 mg/kg/day. This suggests that **T-1-NBAB** has a lower risk of causing cancer at therapeutic doses, making it a safer option for prolonged use.

Considering the acute Toxicity, the maximum tolerated feeding dose in rats for **T-1-NBAB** is 0.026 g/kg, which is considerably lower than sorafenib’s 0.082 g/kg. Furthermore, **T-1-NBAB** exhibits a much higher oral LD_50_ of 7.252 g/kg compared to 0.823 g/kg for sorafenib. This indicates that **T-1-NBAB** is less toxic acutely, allowing for a wider safety margin in dosing.

In terms of chronic toxicity, **T-1-NBAB** showed a higher LOAEL (Lowest Observed Adverse Effect Level) of 0.038 g/kg in rats compared to sorafenib’s 0.005 g/kg. This suggests that **T-1-NBAB** is less toxic over long-term exposure, reducing the risk of adverse effects from extended treatment periods.

Finally, both compounds exhibit mild ocular irritability and no topical irritation. This indicates that neither **T-1-NBAB** nor sorafenib pose significant risks of causing irritation when in contact with eyes or skin, which is beneficial for patient comfort and compliance.

### 2.2. Chemistry

**Scheme 1:**
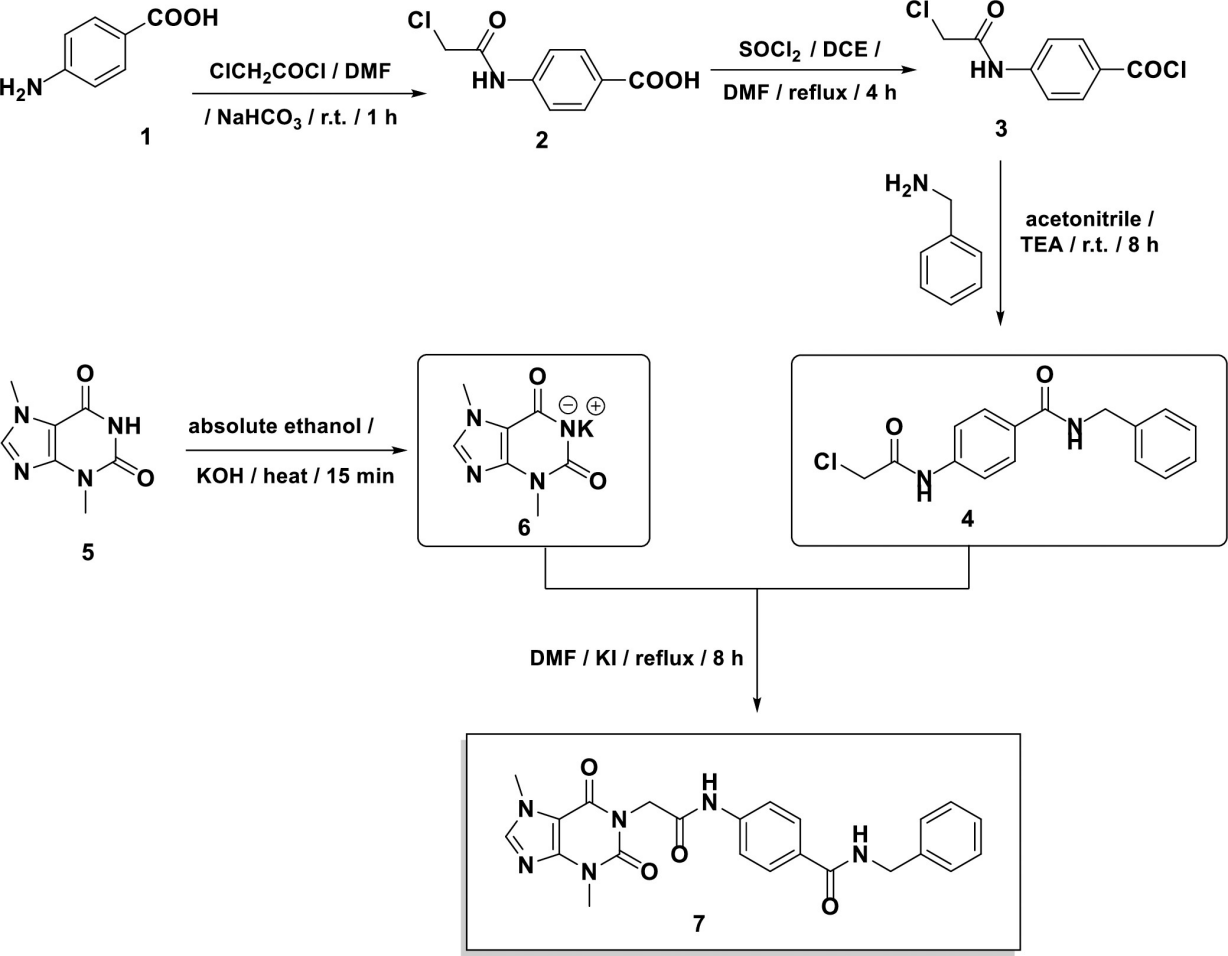
Semi-synthesis of T-1-NBAB.

**T-1-NBAB** was synthesizes as shown in Scheme 1. First, the chloroacetamide derived compound **2** was prepared through the acetylation of p-aminobenzoic acid **1**, using chloroacetyl chloride [70–72]. Then, the carboxylic acid group of **2** reacted with SOCl_2_ to afford acyl chloride **3** [73]. Compound **3** was subsequently reacted with benzylamine in acetonitrile with triethylamine, yielding the desired intermediate *N*-benzyl-4-(2-chloroacetamido)benzamide, **4**, in a high yield. Meanwhile, the theobromine’s potassium salt, 6, was obtained by treating the theobromine, **5**, with alcoholic KOH under continuous stirring [74, 75]. Compound **6** was then refluxed with Compound **4** in DMF, catalyzed by a small amount of KI yielding the final compound **T-1-NBAB**.

EI-MS analysis of **T-1-NBAB** exhibited a molecular ion peak at 446.29 m/z. Elemental analysis yielded C, 62.04%; H, 5.09%; N, 19.04%. IR spectroscopy of **T-1-NBAB** indicated absorption bands characteristic of carbonyl groups at 1704 and 1665 cm^-1^. ^1^H NMR spectroscopy revealed signals at 4.72 and 4.48 ppm corresponding to two methylene groups, along with singlet signals at 10.53 and 8.96 ppm for the amidic protons. In the ^13^C NMR spectrum, distinct peaks at 43.95 and 43.05 ppm (CH_2_ groups) and 33.68 and 29.93 ppm (CH_3_ groups) provided further confirmation of the proposed structure’s validity.

### 2.3. Biology

#### 2.3.1. *In vitro* inhibition of VEGFR-2

The primary objective of designing **T-1-NBAB** was to target VEGFR-2 serving as a principal angiogenesis regulator. Subsequently, *in silico* analysis revealed promising inhibitory properties of **T-1-NBAB**. As a result, **T-1-NBAB** was semi-synthesized to perform further experiments to investigate its *in vitro* potential as a VEGFR-2 inhibitor. Remarkably, **T-1-NBAB** exhibited potent inhibitory activity against VEGFR-2 showing an IC_50_ of 0.115 ± 0.005 μM, comparing sorafenib’s IC_50_ (0.0583±0.003), thereby validating the computational analysis and establishing the **T-1-NBAB**’s strong suppressive potential. These results underscore the significance of the rational design approach and hold promising implications for new VEGFR-2 inhibitor development.

#### 2.3.2. Cytotoxicity and selectivity

The potential of **T-1-NBAB** as an anticancer agent has been highlighted through both *in silico* and *in vitro* studies, primarily due to its anti-VEGFR-2 properties. To further investigate **T-1-NBAB**’s cytotoxicity, it was tested against breast carcinoma epithelial cancer cell lines MCF7 and T47D. The compound showed promising anticancer effects, with IC_50_ values of 16.88 μM and 61.17 μM for MCF7 and T47D cell lines, respectively. In comparison, **T-1-NBAB** had a significantly higher IC_50_ value of 68.44 μM against Vero (normal epithelial) cell lines, indicating a lower toxicity to normal cells.

The selectivity index (SI) measures a compound’s specificity for target cells over non-target cells. It is calculated by dividing the IC_50_ value for normal cells by the IC_50_ value for cancer cells. A higher SI indicates greater selectivity, meaning the compound is more toxic to cancer cells than to normal cells, which is desirable for anticancer agents [76]. The SI values of **T-1-NBAB** against MCF7 and T47D were calculated to be 4.1 and 1.1, respectively. Notably, the treatment of MCF7 cells demonstrated impressive activity, with a high selectivity index value, suggesting that **T-1-NBAB** has significant cytotoxicity specifically towards cancerous cells while sparing normal cells.

#### 2.3.3. Apoptosis assay

**Fig 18:**
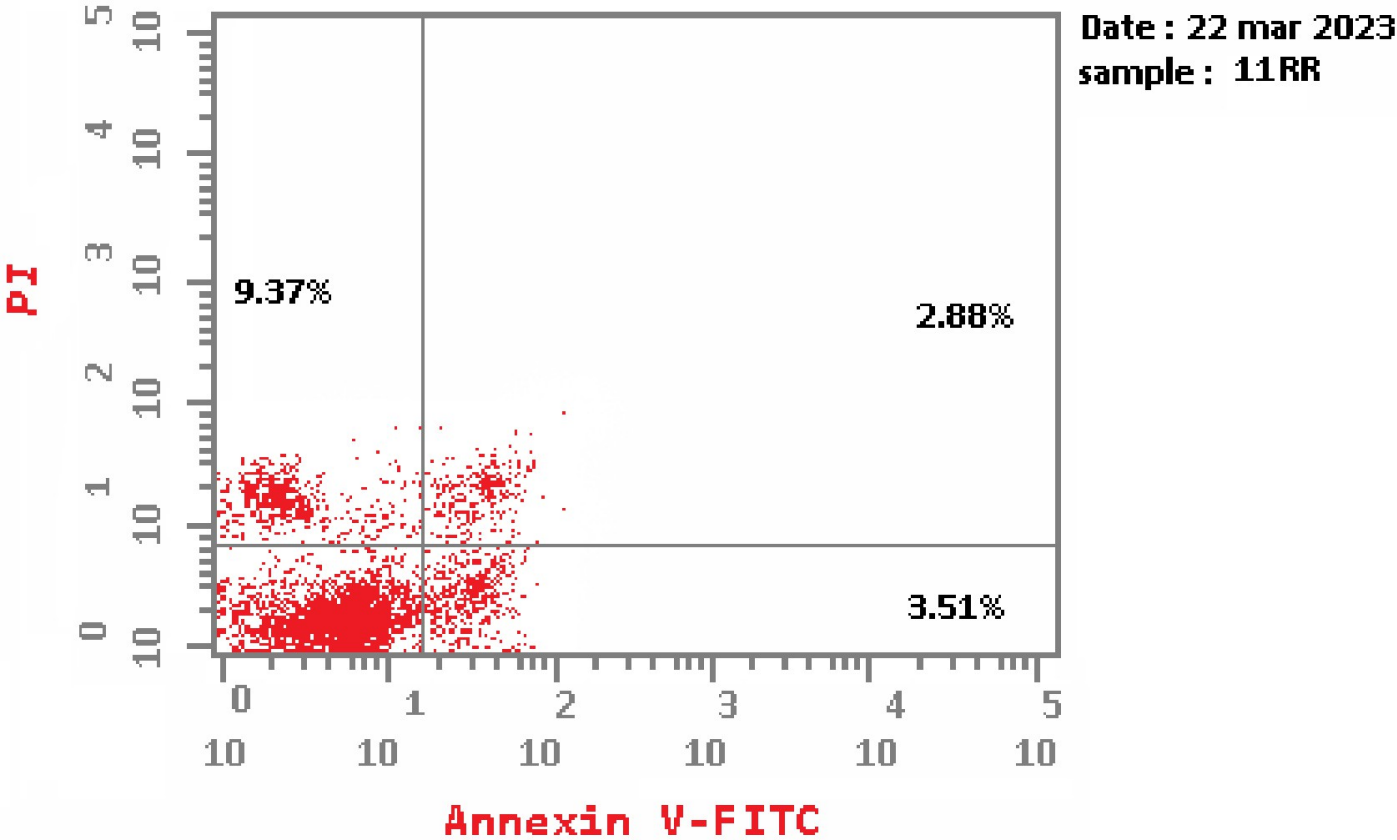
Flow cytometry chart of T-1-NBAB apoptotic potential in MCF7 cells.

The **T-1-NBAB**’s effect as an apoptotic agent was assessed via flow cytometry analysis, on MCF7 cells using Annexin V and PI double staining [72]. Notably, treatment with **T-1-NBAB** induced a statistically significant increase in the proportion of apoptotic MCF7 cells in the early stages of apoptosis (0.72% to 3.51%) and the late stage of apoptosis (0.12% to 2.88%) comparing control. Interestingly, the necrosis percentage increased to 9.37, compared to 2.21% in control (**Table 5** and **Fig 18**). These findings suggest that **T-1-NBAB** may have the potential as an agent for inducing apoptosis in cancer cells.

**Table 5:** T-1-NBAB apoptosis induction against MCF7 cells.

Comp.	Apoptosis			Necrosis
	Total	Early	Late	
**T-1-NBAB**	15.76	3.51	2.88	9.37
**Control**	3.04	0.72	0.12	2.21

#### 2.3.4. The effect of T-1-NBAB on MCF7’s healing and migration

**Fig 19:**
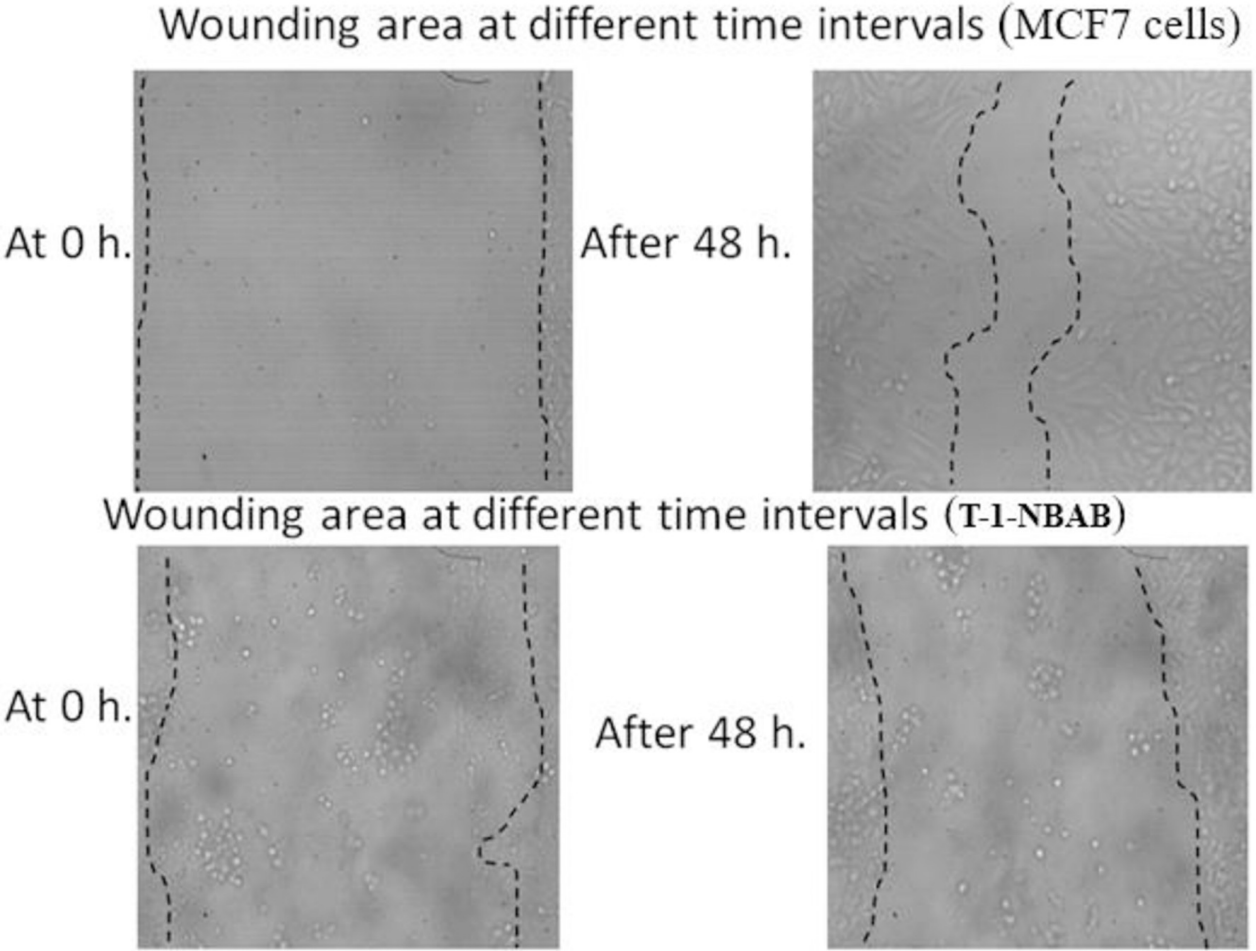
T-1-NBAB’s effect on MCF7’s healing and migration.

The wound healing assay, a simple and cost-effective procedure, can assess the mobility and repair capacity of cancerous cells [77]. In this method, a scratch is created on a layer of cancer cells, and the initial width is recorded. The healing of the scratch was observed periodically for both untreated and treated cells. Throughout the study, images of the scratched areas from both groups were compared at the 0-hour and 48-hour time points [78]. The results (Table 6 and Fig 19) demonstrated that after 48 hours, the untreated MCF7 (control) cells had closed 65.9% of the scratch. On the other hand, the MCF7 cells treated with **T-1-NBAB** only reduced the scratch width by 12%, showing that the **T-1-NBAB** treatment greatly inhibited scratch closure.

**Table 6:** The effect of T-1-NBAB on MCF7’s healing and migration.

Item	at 0h		at 48 h		RM um	Wound closure % um^2^	Area difference %
	area	width	area	width			
Control ^a^	1000.333	999.3853	340.5	339.5385	13.74681	65.96135	659.8333
**T-1-NBAB** ^a^	917	916.0155	805.5	804.5532	2.322132	12.15921	111.5

^a^ Data are presented as a mean of five times values.

## 3. Experimental

### 3.1. Docking studies

Docking was accomplished for **T-1-NBAB** against VEGFR-2 by MOE2014 software [79]. An elaborate and extensive discussions are included in the S1 File (**Molecular Docking studies**).

### 3.2. MD simulations studies

The stability of the VEGFR-2_**T-1-NBAB** complex, the interaction strength, and the differences between VEGFR-2_apo _**T-1-NBAB** and VEGFR-2_holo_**T-1-NBAB** complexes were evaluated through a 100-ns unbiased MD simulation. The input files for the simulation were prepared using CHARMM-GUI web server’s solution builder module [80, 81] in GROMACS 2021 [82]. An elaborate and extensive discussions are included in the S1 File (**Molecular Dynamic Simulation**).

### 3.3. Binding free energy calculation using MM-GBSA

To investigate the interactions in the VEGFR-2_**T-1-NBAB** complex deeper, the gmx_MMPBSA program was employed to assess the binding affinity using the Molecular Mechanics Generalized Born Surface Area (MM-GBSA) [83, 84]. An elaborate and extensive discussions are included in the S1 File (**Binding free energy calculation using MM-GBSA**).

### 3.4. ED analysis

Principal Component Analysis (PCA) was used to investigate the synchronized motion of a specific group of atoms by analyzing the mass-weighted covariance matrix (C) along MD trajectories. In this case, PCA focused on the coordinated mobility of the alpha carbons in the amino acids Glu826 and Leu1161 [85]. To directly compare the frames in the reduced subspace, we followed a series of steps. Initially, we merged the trajectories of VEGFR-2_apo _**T-1-NBAB** and VEGFR-2_holo_**T-1-NBAB** complexes. These combined trajectories were then aligned to the equilibrated state of the apo-protein. Subsequently, we generated a new covariance matrix (C matrix) for the combined trajectories and projected each trajectory onto this matrix. To evaluate the similarity between the VEGFR-2_apo _**T-1-NBAB** and VEGFR-2_holo_**T-1-NBAB** complexes, we plotted their projections onto the first three eigenvectors. By utilizing different pairs of eigenvectors, we were able to visualize and analyze the relationship between the trajectories in the reduced subspace [86].

### 3.5. DFT

DFT investigations were conducted for **T-1-NBAB** by Gaussian 09 and GaussSum3.0 programs. An elaborate and extensive discussions are included in the S1 File (**Density Function Theory (DFT) calculations**).

### 3.6. ADMET studies

ADMET investigations were conducted for **T-1-NBAB** by Discovery Studio 4.0 [87]. An elaborate and extensive discussions are included in the S1 File (**Running of ADMET protocol**).

### 3.7. Toxicity studies

Toxicity investigations were conducted for **T-1-NBAB** by Discovery Studio 4.0 [88]. An elaborate and extensive discussions are included in the S1 File (**Running of Toxicity protocol**).

### 3.8. Semi-synthesis of T-2-PNPA

An elaborate discussion for the semi-synthesis and the EI-Ms, IR, ^1^H NMR, as well as ^13^C NMR of **T-1-NBAB** is included in the S1 File (**Chemistry**).

### 3.9. *In vitro* VEGFR-2 inhibition

Was accomplished for **T-1-NBAB** by Human VEGFR-2 ELISA kit [89]. An elaborate and extensive discussions are included in the S1 File (***In vitro* VEGFR kinase assay**).

### 3.10. *In vitro* cytotoxicity

The cytotoxic potential of **T-1-NBAB** against MCF7 and T47D cell lines was accomplished by MTT procedure [90]. An elaborate and extensive discussions are included in the S1 File (***In vitro* anticancer activity**).

### 3.11. Safety assessment and SI calculation

The cytotoxic potential of **T-1-NBAB** against non-cancerous (Vero) cell lines was accomplished by MTT procedure [91]. An elaborate and extensive discussions are included in the S1 File (**Selectivity index (SI)**).

### 3.12. Flowcytometry

**T-1-NBAB**’s apoptotic potentials and effect on MCF7 cell cycle were accomplished by flow cytometry analysis technique [92]. An elaborate and extensive discussions are included in the S1 File (**Flow cytometry analysis for apoptosis**).

### 3.13. Wound healing assay

Wound healing assay was accomplished for **T-1-NBAB** utilizing MCF7 cell lines [93]. An elaborate and extensive discussions are included in the S1 File (**Wound healing and migration assay**).

## 4. Conclusion

The study presented a CADD approach to design and evaluate a new compound, **T-1-NBAB**, as a VEGFR-2 inhibitor for potential therapeutic applications in breast cancer. Employing various computational techniques such as molecular docking, molecular dynamics simulations, MM-GPSA, PLIP, essential dynamics, and bi-dimensional projection experiments, **T-1-NBAB**’s binding and inhibiting potential against VEGFR-2 was indicated. Additionally, DFT studies analyzed **T-1-NBAB**’s 3D structure, electrostatic potential, global reactive indices, and total density of states. Fascinatingly, the *in silico* and *in vitro* results were in agreement as **T-1-NBAB** effectively inhibited VEGFR-2 and exhibited significant activities against MCF7 and T47D breast cancer cell lines with high selectivity values. Interestingly, **T-1-NBAB** induced both early and late apoptosis in MCF7 cell lines. Moreover, **T-1-NBAB** significantly reduced MCF7 cell migration and healing potential, suggesting its possible application as an anti-angiogenetic agent. Overall, the findings employed in this study indicate that **T-1-NBAB** could be a promising candidate for further research as a potential treatment for breast cancer. Accordingly, in the future plan, the central phenyl group will be modified to be a heterocyclic ring. This modification will may increase the water solubility and the binding affinity against VEGFR-2 hoping to increase the anti-proliferative and VEGFR-2 inhibitory activities.

## Supporting information

S1 File Contain an elaborative discussion of the employed methodologies, Spectral Data (EI-Ms, IR, ^1^H NMR, as well as ^13^C NMR of T-1-NBAB), S.1 Table and S.1 Fig illustrate the QTAIM parameters (a.u.) and the bond critical points (BCPs) of T-1-NBAB, respectively. Finally, a detailed toxicity report for compounds with high degree of structural similarities to **T-1-NBAB** that exhibited experimental toxicity or safety before.
(PDF)
