# Supplementary material for: Integrated in silico and in vitro exploration of the anti-VEGFR-2 activities of a semisynthetic xanthine alkaloid inhibiting breast cancer
Source: PLoS One. 2025 Jan 27;20(1):e0316146. doi: 10.1371/journal.pone.0316146 (PMC11771932; doi:10.1371/journal.pone.0316146)
Supplement: S1 File — Finally, a detailed toxicity report for compounds with high degree of structural similarities to T-1-NBAB that exhibited experimental toxicity or safety before. (PDF) [file pone.0316146.s001.pdf]

Supplementary file

Integrated In silico and In vitro Exploration of the Anti-VEGFR-2 Activities of a Semisynthetic Xanthine Alkaloid Inhibiting Breast Cancer

Eslam B. Elkaeed 1, Hazem Elkady2*, Ahmed M. Khattab 2, Reda G. Yousef 2, Hanan A. Al-ghulikah 3, Dalal Z. Husein4, Ibrahim M. Ibrahim 5, Mohamed A. Elkady 6, Ahmed M. Metwaly 7* Ibrahim H. Eissa2*

[a] Pharmaceutical Medicinal Chemistry & Drug Design Department, Faculty of Pharmacy (Boys), Al-Azhar University, Cairo11884, Egypt. ahmedmustafa.2.stu.1@azhar.edu.eg; redayousof@azhar.edu.eg Hazemelkady@azhar.edu.eg

[b] Department of Pharmaceutical Sciences, College of Pharmacy, AlMaarefa University, Riyadh 13713, Saudi Arabia. ikaeed@mcst.edu.sa

[c] Department of Pharmaceutical Sciences, College of Pharmacy, Princess Nourah bint Abdulrahman University, P.O. Box 84428, Riyadh 11671, Saudi Arabia aaalSfouk@pnu.edu.sa;

[d] Chemistry Department, Faculty of Science, New Valley University, El-Kharja 72511, Egypt. dalal_husein@sci.nvu.edu.eg

[e] Biophysics Department, Faculty of Science, Cairo University. Cairo 12613, Egypt. ibrahim_mohamed@cu.edu.eg

[f] Pharmacognosy and Medicinal Plants Department, Faculty of Pharmacy (Boys), Al-Azhar University, Cairo 11884, Egypt.
[g] Biopharmaceutical Products Research Department, Genetic Engineering and Biotechnology Research Institute, City of Scientific Research and Technological Applications (SRTA-City), Alexandria, Egypt.

Content

**Method**	**Molecular Docking**
	**MD Simulations**
	**MM-GBSA**
	**DFT**
	**Essential dynamicsstudies**
	**ADMET studies**
	**Semi Synthesis**
	***In vitro* assays**
**Spectral Data**	**IR**
	**^{1}H and ^{13}C NMR**
**Table S.1. QTAIM parameters (a.u.) at bond critical points (BCPs) of T-1-NBAB**	
**Figure S.1. QTAIM analysis: molecular graph showing the bonding critical points (BCP) for T-1-NBAB**	
**Toxicity report**	

Method

- **Molecular Docking studies**

Protein Preparation: The crystal structure of VEGFR-2 [PDB ID: PDB ID: 2OH4, resolution: 2.05 Å] was obtained from Protein Data Bank (https://www.rcsb.org). At first, the crystal structure of the VEGFR-2 complexed with (sorafenib) the co-crystallized ligand was prepared by removing crystallographic water molecules. Only one chain was retained besides the co-crystallized ligand. The selected protein chain was protonated using the following setting. The used electrostatic functional form was GB/VI with a distance cut-off of 15 Å. The used value of the dielectric constant was 2 with an 80 dielectric constant of the used solvent. The used Van der Waals functional form was 800R3 with a distance cut-off of 10 Å. Then, the energy of the protein chain was minimized using Hamiltonian AM1 implanted in Molecular Operating Environment (MOE 2019 and MMFF94x (Merck molecular force field) for structural optimization. Next, the active site of the target protein was defined for ligand docking and redocking (in case of validation of docking protocol). The active site of the protein was identified as the residues that fall within the 5 Å distance from the perimeter of the co-crystallized ligand.

Ligand Preparation: 2D structures of **T-1-NBAB** and the standard compound, sorafenib, were drawn using ChemBioDraw Ultra 14.0 and saved in MDL-SD file format. The 3D structures of the ligands were protonated, and the structures were optimized by energy minimization using MM2 force-field and 10000 iteration steps of 2 fs. The conformationally optimized ligands were used for docking studies.

Docking Setup and Validation of Docking Protocol: The protein-ligand docking studies were carried out using MOE version 2019. Validation of the docking protocol was carried out by redocking the co-crystallized reference ligand (sorafenib) against the isolated pocket of VEGFR-2. The docking protocol was validated by comparing the heavy atoms RMSD value of the re-docked ligand pose with the corresponding co-crystallized reference ligand structure.

The docking setup for **T-1-NBAB** was established according to the protocol followed in the validation step. For each docking run, 30 docked solutions were generated using ASE for scoring function and rigid receptor for refinement. The pose with ideal binding mode was selected for further investigations. The docking results were visualized using Discovery Studio (DS) 4.0. Analysis of the docking results was carried out by comparing the interactions and docking score obtained for the docked ligands with that of the re-docked reference molecule (sorafenib).

- **Molecular Dynamic Simulation**

The stability of the VEGFR-2_**T-1-NBAB**complex, the strength of interactions and the differences between the apo and holo structures were evaluated by running a 100-ns classical unbiased MD simulation in GROMACS 2021 (1). The CHARMM-GUI web server's solution builder module was used to prepare the input files (2–5). First, each system was uploaded as a PDB file, then the transferable intermolecular potential 3 points (TIP3P) water model was used to solvate the complex in a cubic box with 1 nm padding, and finally, the box was neutralized with Na^{+}, and Cl^{-} ions at a concentration of 0.154 M to approximate physiological salt content. The parameters for the VEGFR-2 protein's amino acids, the TIP3P water model, and the neutralizing ions were obtained using the modified Chemistry at Harvard Macromolecular Mechanics force field (CHARMM36m). Furthermore, the T-1-NBABcompound was parameterized using the CHARMM general force field (CGenFF) tool in CHARMM-GUI.

The dynamics were carried out using GROMACS 2021, with periodic boundary conditions (PBC) applied to the system in all three spatial dimensions. The potential energy of the solvated and neutralized system was reduced before the equilibration run to prevent atom collisions. Then, the pressure and temperature were modified. The minimization process was initiated with a maximum force on any atom set at 100 $KJ.mol^{-1}.nm^{-1}$ as a convergence criterion. Canonical (NVT) ensemble equilibration was performed first, followed by isothermal-isobaric (NPT) ensemble equilibration. The temperature and pressure were set to 310 K and 1 atm using the V-rescale algorithm and the Berendsen barostat, respectively (6). With the Nose-Hoover thermostat maintaining the temperature steady at 310 K, the production run was started for 100 ns in an NVT ensemble (7). H-bond atoms' bond lengths were restricted in each step using the LINear Constraint Solver (LINCS) (7). Electrostatics were calculated using a Particle Mesh Ewald (PME) method at a threshold of 1.2 nm (8). To integrate the Newtonian equations of motion, a leap-frog approach

was used, with a time step of 1 femtosecond for equilibration and 2 femtoseconds for production. During the production run, one frame was collected every 0.1 ns, for a total of 1000 frames. Before analyzing the trajectory, the PBC was removed from the system using GROMACS' trjconv tool and the production run was analyzed using VMD TK scripts (9). Separate Root Mean Square Deviation (RMSD) values were calculated for the VEGFR-2 protein and the **T-1-NBAB** molecule. In addition, the distance between the ligand and protein centers of mass, the root mean square fluctuation (RMSF), solvent accessible surface area (SASA), the radius of gyration (RoG), and number of hydrogen bonds were calculated. Next, we clustered the trajectory using TTClust to make the data more manageable and obtained a representative frame for each cluster (10). Before identifying the optimal number of clusters using the elbow approach, backbone alignment was done to remove translational and rotational motion. The protein-ligand interaction profiler (PLIP) was used to count and identify interactions in each representative frame (11).

Essential dynamics (Principal Component Analysis):

PCA of the mass-weighted covariance matrix (C) of a selected group of atoms reveals correlated mobility along MD trajectories. In this case, PCA was used to observe the movement of alpha carbons in amino acids. Glu826:Leu1161 (12). The equilibrium stage's final frame was employed as the alignment reference frame in single-trajectory analysis. For the purpose of assessing the combined trajectories, however, the apo system's final frame after equilibration was utilized as the reference structure. By diagonalizing the C matrix, PCA can determine which eigenvectors and eigenvalues are most suited to capture atomic motions. The eigenvalue of a system is biggest for its first principal component, and decreases for consecutive PCs, suggesting less motion. gmx covar was used to perform the diagonalization of the C matrix in GROMACS, and gmx anaeig was used for the analysis.

When determining the size of the essential subspace, we considered 1) the cumulative sum of the eigenvalues with additional eigenvectors. 2) the location of the largest decrease in the slope of the line between the eigenvalues and eigenvector index number (scree plot). 3) Since it is well-established that non-random eigenvectors do not follow a Gaussian distribution, the distribution of the eigenvectors was also considered.

From the C matrix, we calculated the cosine content (ci) of each eigenvector of the C matrix, which can take on values between 0 (no cosine) and 1 (perfect cosine). The equation of cosine content is as follows:

$$c_i = \frac{2}{T} (\int \cos (i\pi t \, p_i(t) \, dt)^2 (\int p_i^2 (t) \, dt)^{-1}$$

Where T is the time of the simulation. Abnormally large ci values, which represent random motion, are related to insufficient sampling. When the cosine content of the first few PCs is near 1, the behavior of proteins on a large scale is analogous to diffusion. Accordingly, the first 10 PCs were used to calculate their cosine content (13–15).

Aligning the combined apo-protein and complex trajectories to the apo-protein configuration acquired after equilibration, constructing a new C matrix for the combined trajectories, and then projecting each trajectory onto the new C matrix allowed us to directly compare the frames in the reduced essential subspace. We were able to evaluate the degree of similarity between the two trajectories by projecting each onto the first three eigenvectors using different eigenvector pairs.

Binding free energy calculation using MM-GBSA:

With the use of the gmx_MMPBSA program, we were able to evaluate the binding strength using the Molecular Mechanics Generalized Born Surface Area (MM-GBSA) approach. Amino acids within 1 nm of the ligand were employed in a decomposition study to measure their contribution to the binding energy (16,17). Both the ionic strength and the solvation method (igb) were adjusted

to 0.154 M and 5, respectively. All other parameters were kept at their default settings except for the dielectric constants, which were adjusted to 1.0 for the inside and 80.0 for the outside. The MM-GBSA procedure is shown in Equation 1.

$$\Delta G = < G_{complex} - (G_{receptor} + G_{ligand}) > \quad \text{Equation 1}$$

Where < > represents the average of the enclosed free energies of complex, receptor, and ligand over the frames used in the calculation. In our approach, we used the whole trajectory (a total of 1000 frames). Different energy terms can be calculated according to Equations 2 to 6 as follows:

$$\Delta G_{binding} = \Delta H - T\Delta S \quad \text{Equation 2}$$

$$\Delta H = \Delta E_{gas} + \Delta E_{sol} \quad \text{Equation 3}$$

$$\Delta E_{gas} = \Delta E_{ele} + \Delta E_{vdW} \quad \text{Equation 4}$$

$$\Delta E_{solv} = E_{GB} + E_{SA} \quad \text{Equation 5}$$

$$E_{SA} = \gamma.SASA \quad \text{Equation 6}$$

Where:

ΔH is the enthalpy which can be calculated from gas-phase energy (E_{gas}) and solvation-free energy (E_{sol}). -TΔS is the entropy contribution to the free binding energy. E_{gas} is composed of electrostatic and van der Waals terms; E_{ele}, E_{vdW}, respectively. E_{sol} can be calculated from the polar solvation energy (E_{GB}) and nonpolar solvation energy (E_{SA}) which is estimated from the solvent-accessible surface area (18,19).

- **Density Function Theory (DFT) calculations**

The Gaussian 09 program was used to perform the quantum chemistry calculations using the DFT method. GaussianView5 was used to display all of the data files. The density function theory (DFT) at 6-311G++(d,p) basis set/B3LYP approach was utilized to optimize organic chemical structure of the compound under investigation and Chem3D 15.0 software was used to create the original chemical structures. Both the Total Electron Density (TED) and the Electrostatic Surface (ESP) maps were examined at the same theoretical level.

GaussSum3.0 software was used to compute and evaluate the total density of state (TDOS) for the optimized log file.

Equations of Koopmans' theory: The chemical potential (μ), ma**xi**mal charge acceptance (ΔN_{max}), global hardness (η), energy change (ΔE), electronegativity (χ), the global softness (σ), electrophilicity index (ω), ionization potential (IP) and electron affinity (EA)

$IP = -E_{HOMO}$

$EA = -E_{LUMO}$

$\mu =$
$(IP+EA)/2$

$\eta = (IP-EA)$

$\chi = -\eta$

$\omega = \mu^2/(2$
$\eta)$ $\sigma = 1/$
η

$\Delta N = -(\mu/\eta)$

$\Delta E = -\omega$

$E_{gap} = E_{LUMO} - E_{HOMO}$

- **Preparation of T-1-NBAB for ADMET and toxicity studies:**

In this protocol, the general-purpose panel was utilized with the activation of the Prepare ligand option. The change ionization was switched on the true option using the Rule based as an ionization method. In Rule based task, we used the carboxylate as an acid ionization. Additionally, the primary, secondary, and tertiary amines were selected as Base ionization. The ionization enumeration option was switched on the one protomer. Under the filter smart option, we selected all options. The false option was selected for tasks Generate tautomers, generate isomers, Fix bad valencies, and parallel processing. The generate coordinates task was switched on the 3D option. Finally, the duplicate structure task was activated on the remove option.

ADMET descriptors (absorption, distribution, metabolism, excretion and toxicity) of **T-1-NBAB** were determined using Discovery studio 4.0. **Sorafenib** was used as a reference molecule. At first,

the CHARMM force field was applied then the tested compounds were prepared and minimized according to the preparation of small molecule protocol. The ADMET descriptors that applied including models for

1. Human intestinal absorption,
2. Aqueous solubility,
3. Blood brain barrier penetration,
4. Plasma protein binding,
5. Cytochrome P450 2D6 inhibition, and
6. Hepatotoxicity.

Running of ADMET protocol

In this protocol, after compounds preparation, the small molecules panel was utilized with the activation of the ADMET descriptors option. Then, we selected the prepared compounds as the input ligands. Further, all the ADMET parameters (aqueous solubility, Blood brain barrier, intestinal absorption, CYP2D6, and plasma protein binding) were selected. Then, the output of the running protocol was visualized to give the ADMET chart.

- **Toxicity studies**

The toxicity parameters of **T-1-NBAB** were calculated using Discovery studio 4.0. Sorafenib was used as a reference molecule. Then different parameters were calculated from the toxicity prediction (extensible) protocol (TOPKAT) that evaluated the examined compounds' performance in experimental assays and animal models. TOPKAT computed and validated assessments of the toxic and environmental effects of the examined chemicals solely from their molecular structure. TOPKAT employs robust and cross-validated Quantitative Structure Toxicity Relationship (QSTR) models for assessing various measures of toxicity and utilizing the patented Optimal Predictive Space validation method to assist in interpreting the results.

The predicted models are

1. FDA rat carcinogenicity test,
2. Carcinogenic potentiality TD_{50} (the median toxic dose of a substance in which toxicity occurs in 50% of a species),
3. Maximum tolerated dose (MTD) in rats,
4. Oral LD_{50} in rats (the amount that kills 50% of test animals),
5. Chronic LOAEL (Lowest-observed-adverse-effect level) in rats,

6. Ocular irritancy and
7. Skin irritancy

Running of Toxicity protocol

In this protocol, after compounds preparation, the small molecules panel was utilized with the activation of the toxicity prediction (extensible) option. Then, we selected **T-1-NBAB** and sorafenib as the input ligands. Further, the different toxicity models were selected from the model panel. The similarity search task was activated to be true. The detailed report task was switched on as a PDF file. Then, the output of the running protocol was visualized to give the toxicity PDF report.

- **Chemistry**

General

The melting point were carried out by open capillary method on a Gallen kamp Melting point apparatus. The infrared spectra were recorded on pye Unicam SP 1000 IR spectrophotometer using potassium bromide disc technique. Proton magnetic resonance ^{1}HNMR spectra were recorded on a Bruker 400 Megahertz-nuclear magnetic resonance (400 MHZ-NMR) spectrophotometer. Carbon-13 (C13) nuclear magnetic resonance (^{13}CNMR) spectra were recorded on a Bruker 100 Megahertz-nuclear magnetic resonance (100 MHZ-NMR) spectrophotometer. Tetramethylsilane (TMS) was used as internal standard and chemical shifts were measured in δ scale one part per million (ppm). The reactions were monitored by thin-layer chromatography (TLC) using TLC sheets precoated with UV fluorescent silica gel Merck 60 F254 plates and were visualized using ultraviolet (UV) lamp and different solvents as mobile phases.

General procedure for the semi-synthesis of T-1-NBAB

N-Benzyl-4-(2-chloroacetamido)benzamide **4** (0.001 mol) was added to a solution of the potassium salt of 3,7-dimethyl-3,7-dihydro-1*H*-purine-2,6-dione **6** (0.001 mol) in dry DMF (10 mL), and the mixture was heated in a water bath for 8 hours. After being poured onto 200 mL of ice water, the reaction mixture was stirred for 30 minutes. To obtain the desired **T-1-NBAB**, the precipitate was filtered, water washed, and crystallized from ethanol.

***N*-Benzyl-4-(2-(3,7-dimethyl-2,6-dioxo-2,3,6,7-tetrahydro-1*H*-purin-1-yl)acetamido)benzamide**

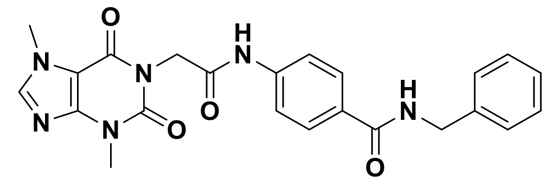

White crystal (yield, 80 %); m. p. = 224-225 °C; IR (KBr) *v* cm^{-1}: 3196 (NH), 1704, 1665 (C=O); ^{1}H NMR (400 MHz, DMSO-d_6) δ 10.53 (s, 1H), 8.96 (t, *J* = 6.1 Hz, 1H), 8.07 (s, 1H), 7.88 (d, *J* = 8.5 Hz, 2H), 7.66 (d, *J* = 8.4 Hz, 2H), 7.33 (d, *J* = 4.4 Hz, 5H), 7.24 (dt, *J* = 9.1, 4.4 Hz, 1H), 4.72 (s, 2H), 4.48 (d, *J* = 6.0 Hz, 2H), 3.90 (s, 3H), 3.45 (s, 3H); ^{13}C NMR (101 MHz, DMSO-d_6) δ 166.62, 166.15, 154.64, 151.36, 148.99, 143.74, 141.81, 140.23, 129.46, 128.74 (2C), 128.69 (2C), 127.70 (2C), 127.18, 118.76 (2C), 107.05, 43.95, 43.05, 33.68, 29.93. Anal. Calcd. for $C_{23}H_{22}N_6O_4$ (446.47); found (446.29): C, 61.88; H, 4.97; N, 18.82; Found: C, 62.04; H, 5.09; N, 19.04 %.

- **Biological evaluations**

Mammalian cell lines culture

MCF7 and T47D cell lines were cultured on DMEM media. The cultured media were supplemented with 200 mM L-glutamine, 10.0% fetal bovine serum (Lonza), and 1.0% penicillin/streptomycin. Cells were seeded into 25.0 cm tissue culture flasks and incubated at 37°C in a 5.0% CO_2 incubator for 24 h or till confluency.

Safety assay

The safety profiles of the tested compounds were checked on one non-cancerous cell line (vero) to determine the treatments concentrations that do not depict toxic effects against the tested cells. A portion of 100.0 µl of 6×10^4 cell/ml cells was seeded into each well of a 96-well plate and then the plates were incubated at 37°C in a humidified 5.0% CO_2 incubator for 24 h. At the end of incubation period, the exhausted medium was replaced with 100.0 µl of different concentrations of the designated treatment (prepared in RPMI medium starting from 1.0 mM). The inoculated plates were incubated at the same growth conditions for another 24 h. At the end of incubation, cellular viability was assessed using MTS assay kit (Promega) according to the manual instruction

In-vitro anticancer activity

Anticancer activities of the tested compounds against MCF7 and T47D cell lines were quantified using MTS assay kit (Promega) as described by the Manufacturer.

Selectivity index (SI)

The selectivity index values of the tested compounds on cancer cells were calculated as described by Koch et al., with slight modifications; SI=IC50nc/IC50cc, where IC50nc: the IC50 value of the tested compound on normal cells and IC50cc: IC50 of the tested compound on cancer cell line**.**

In vitro EGFR kinase assay

The synthesized compound was estimated for their *in vitro* inhibition on human VEGFR-2 in **MCF- 7** cell line; using ELISA kit. Firstly, a plate was used for the assay had been coated by an antibody specific for human VEGFR-2 enzyme, Sorafenib was nominated as a standard VEGFR-2 inhibitor. Both standard and sample were added to the wells and incubated overnight at 4 ◦C, then washed. The biotinylated antibody was supplemented and further incubated for 1 h at room temperature. The unreacted, liberated antibody was then washed; followed by addition of HRP-conjugated streptavidin and incubated for 45 min at room temperature. Wells were washed and a TMB substrate solution was added and kept at room temperature for 30 min. Finally, the stop solution was added, and the intensity of the color produced was measured at 450 nm. Concentration- inhibition response curve was established by GraphPad Prism 5.0. The IC_{50} value was calculated as the concentration at which 50% of the cells could survive in comparison to sorafenib.

Flow cytometry analysis for apoptosis

Flow cytometry cell apoptosis analysis was used to investigate the apoptotic effect of the synthesized compound. A549 cells were treated with compound **T-1-NBAB** for 72h, collected by trypsin, centrifuged, washed two successive times with PBS, suspended in 500 µl binding buffer, and double stained with 5 µl Anne**xi**n V-FITC and 5 µl PI in the dark at room temperature for 15 min. The stained cells were measured using Epics XL-MCL™ Flow Cytometer and analyzed using Flowing software.

Wound healing and migration assay

The wound healing assay allows the researcher to study cell migration and cell interactions. In

some cases also single cell migration can be analyzed and this assay can be imaged

Protocol

1. Cells were seeded in 6 multi-well plates and cultured until confluent. It is important that all the cultures are confluent at the start of the experiment.

2. (yellow) pipette tip was used to make a straight scratch, simulating a wound. Often we make a scratch keeping the pipette tip under an angle of around 30 degrees to keep the scratch width limited. This allows imaging of both wound edges using the 10x objective.

The following equations are applied to calculate the healing and migration ratio

Rat of migration (RM) = (wi – wf) / t

Wi =average of intial wound width um
Wf= average of final wound width um
T = time span of the assay in hours

Percentage of wound clouser % ={ ($A_{t=0}$ – $A_{t=\Delta t}$) / $A_{t=0}$ } 100

$A_{t=0}$ = intial wound area
$A_{t=\Delta t}$ = wound area after n hours

Area difference % = (Ai – Af)

Ai = intial area
Af = final area

References:

1. Abraham MJ, Murtola T, Schulz R, Páll S, Smith JC, Hess B, et al. GROMACS: High performance molecular simulations through multi-level parallelism from laptops to supercomputers. SoftwareX. 2015;1:19–25.
2. Brooks BR, Brooks III CL, Mackerell Jr AD, Nilsson L, Petrella RJ, Roux B, et al. CHARMM: the biomolecular simulation program. J Comput Chem. 2009;30(10):1545–614.
3. Jo S, Cheng X, Islam SM, Huang L, Rui H, Zhu A, et al. Chapter Eight - CHARMM-GUI PDB Manipulator for Advanced Modeling and Simulations of Proteins Containing Nonstandard Residues. In: Karabencheva-Christova T, editor. Biomolecular Modelling and Simulations. Academic Press; 2014. p. 235–65. (Advances in Protein Chemistry and Structural Biology; vol. 96).
4. Jo S, Kim T, Iyer VG, Im W. CHARMM-GUI: A web-based graphical user interface for CHARMM. J Comput Chem. 2008;29(11):1859–65.
5. Lee J, Cheng X, Swails JM, Yeom MS, Eastman PK, Lemkul JA, et al. CHARMM-GUI input generator for NAMD, GROMACS, AMBER, OpenMM, and CHARMM/OpenMM simulations using the CHARMM36 additive force field. J Chem Theory Comput. 2016;12(1):405–13.
6. Bussi G, Donadio D, Parrinello M. Canonical sampling through velocity rescaling. J Chem Phys. 2007;126(1):14101.
7. Hess B, Bekker H, Berendsen HJC, Fraaije JGEM. LINCS: a linear constraint solver for molecular simulations. J Comput Chem. 1997;18(12):1463–72.
8. Essmann U, Perera L, Berkowitz ML, Darden T, Lee H, Pedersen LG. A smooth particle mesh Ewald method. J Chem Phys. 1995;103(19):8577–93.
9. Humphrey W, Dalke A, Schulten K. VMD: visual molecular dynamics. J Mol Graph. 1996;14(1):33–8.
10. Tubiana T, Carvaillo JC, Boulard Y, Bressanelli S. TTClust: a versatile molecular simulation trajectory clustering program with graphical summaries. J Chem Inf Model. 2018;58(11):2178–82.
11. Salentin S, Schreiber S, Haupt VJ, Adasme MF, Schroeder M. PLIP: fully automated protein–ligand interaction profiler. Nucleic Acids Res. 2015 Jul 1;43(W1):W443–7.
12. Amadei A, Linssen ABM, Berendsen HJC. Essential dynamics of proteins. Proteins: Structure, Function, and Bioinformatics. 1993;17(4):412–25.
13. Papaleo E, Mereghetti P, Fantucci P, Grandori R, de Gioia L. Free-energy landscape, principal component analysis, and structural clustering to identify representative conformations from molecular dynamics simulations: the myoglobin case. J Mol Graph Model. 2009;27(8):889–99.
14. Maisuradze GG, Leitner DM. Free energy landscape of a biomolecule in dihedral principal component space: Sampling convergence and correspondence between structures and minima. Proteins: Structure, Function, and Bioinformatics. 2007;67(3):569–78.
15. Hess B. Similarities between principal components of protein dynamics and random diffusion. Phys Rev E. 2000;62(6):8438.
16. Valdés-Tresanco MS, Valdés-Tresanco ME, Valiente PA, Moreno E. gmx_MMPBSA: A New Tool to Perform End-State Free Energy Calculations with GROMACS. J Chem Theory Comput. 2021;17(10):6281–91.
17. Miller III BR, McGee Jr TD, Swails JM, Homeyer N, Gohlke H, Roitberg AE. MMPBSA. py: an efficient program for end-state free energy calculations. J Chem Theory Comput. 2012;8(9):3314–21.
18. Xue W, Yang F, Wang P, Zheng G, Chen Y, Yao X, et al. What contributes to serotonin--norepinephrine reuptake inhibitors' dual-targeting mechanism? The key role of transmembrane domain 6 in human serotonin and norepinephrine transporters revealed by molecular dynamics simulation. ACS Chem Neurosci. 2018;9(5):1128–40.
19. Tuccinardi T. What is the current value of MM/PBSA and MM/GBSA methods in drug discovery? Vol. 16, Expert opinion on drug discovery. Taylor \& Francis; 2021. p. 1233–7.

Spectral data

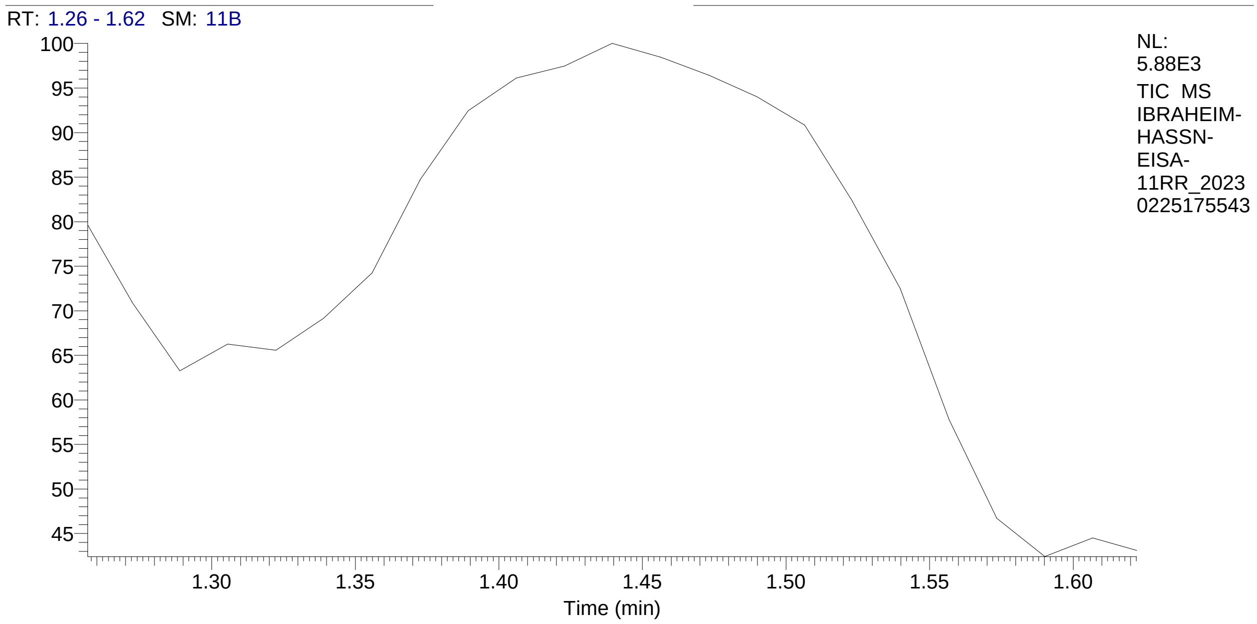

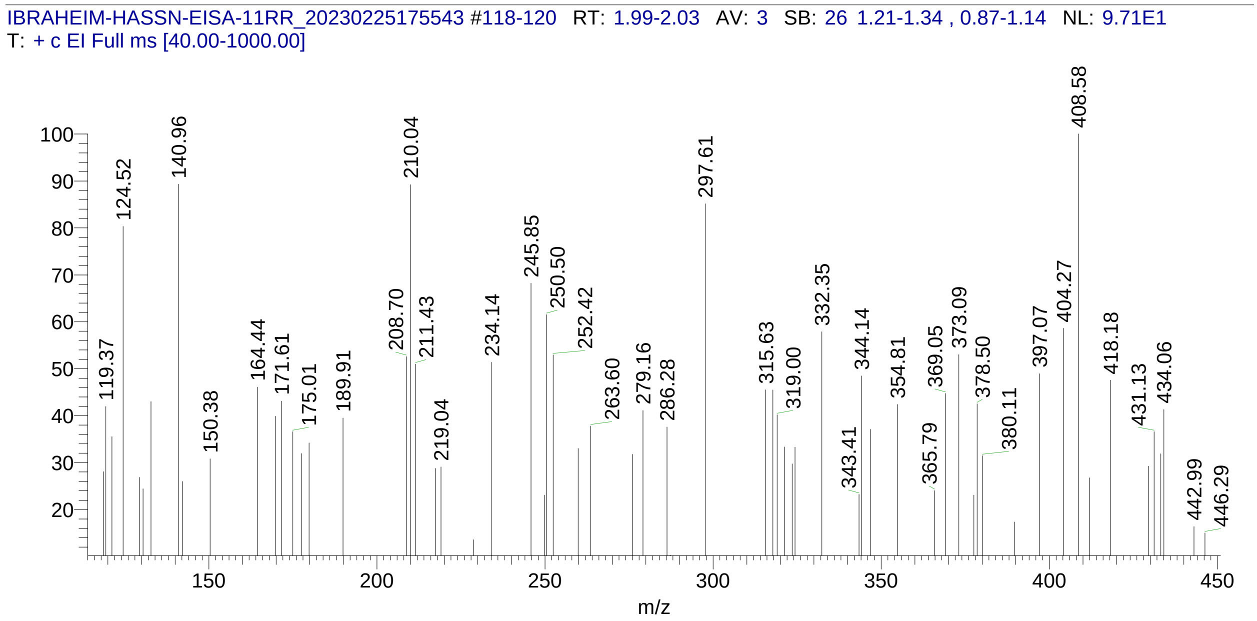

m/z	Intensity	Relative
118.64	27.2	28.07
119.37	40.7	41.94
121.21	34.5	35.51
124.52	78.0	80.31
129.42	26.0	26.83
130.44	23.7	24.42

132.76	41.7	42.99
140.96	86.7	89.29
142.20	25.2	25.96
150.38	29.9	30.80
164.44	44.7	46.06
169.92	38.7	39.88
171.61	41.8	43.09
175.01	35.5	36.58
177.66	31.0	31.91
179.81	33.2	34.16
189.91	38.3	39.46
208.70	51.0	52.56
210.04	86.6	89.19
211.43	49.5	50.98
217.50	27.9	28.75
219.04	28.2	29.07
228.79	13.2	13.57
234.14	49.9	51.37
245.85	66.2	68.22
249.89	22.4	23.05
250.50	59.7	61.54
252.42	51.4	52.93
259.83	32.0	33.01
263.60	36.7	37.77
276.06	30.8	31.76
279.16	39.9	41.07
286.28	36.5	37.56

287.19	9.9	10.20
297.61	82.6	85.13
315.63	44.2	45.51
317.73	44.1	45.47
319.00	39.0	40.16
321.30	32.3	33.30
323.48	28.8	29.72
324.32	32.3	33.27
332.35	56.2	57.86
343.41	22.5	23.21
344.14	47.0	48.44
346.78	36.0	37.10
354.81	41.2	42.40
365.79	23.3	24.04
369.05	43.4	44.73
373.09	51.4	53.00
377.57	22.4	23.05
378.50	41.2	42.50
380.11	30.5	31.42
389.70	16.8	17.32
397.07	47.5	48.95
404.27	56.9	58.61
408.58	97.1	100.00
411.90	26.0	26.78
418.18	46.2	47.56
429.46	28.4	29.21
431.13	35.5	36.58

433.13	31.0	31.89
434.06	40.1	41.30
442.99	15.9	16.35

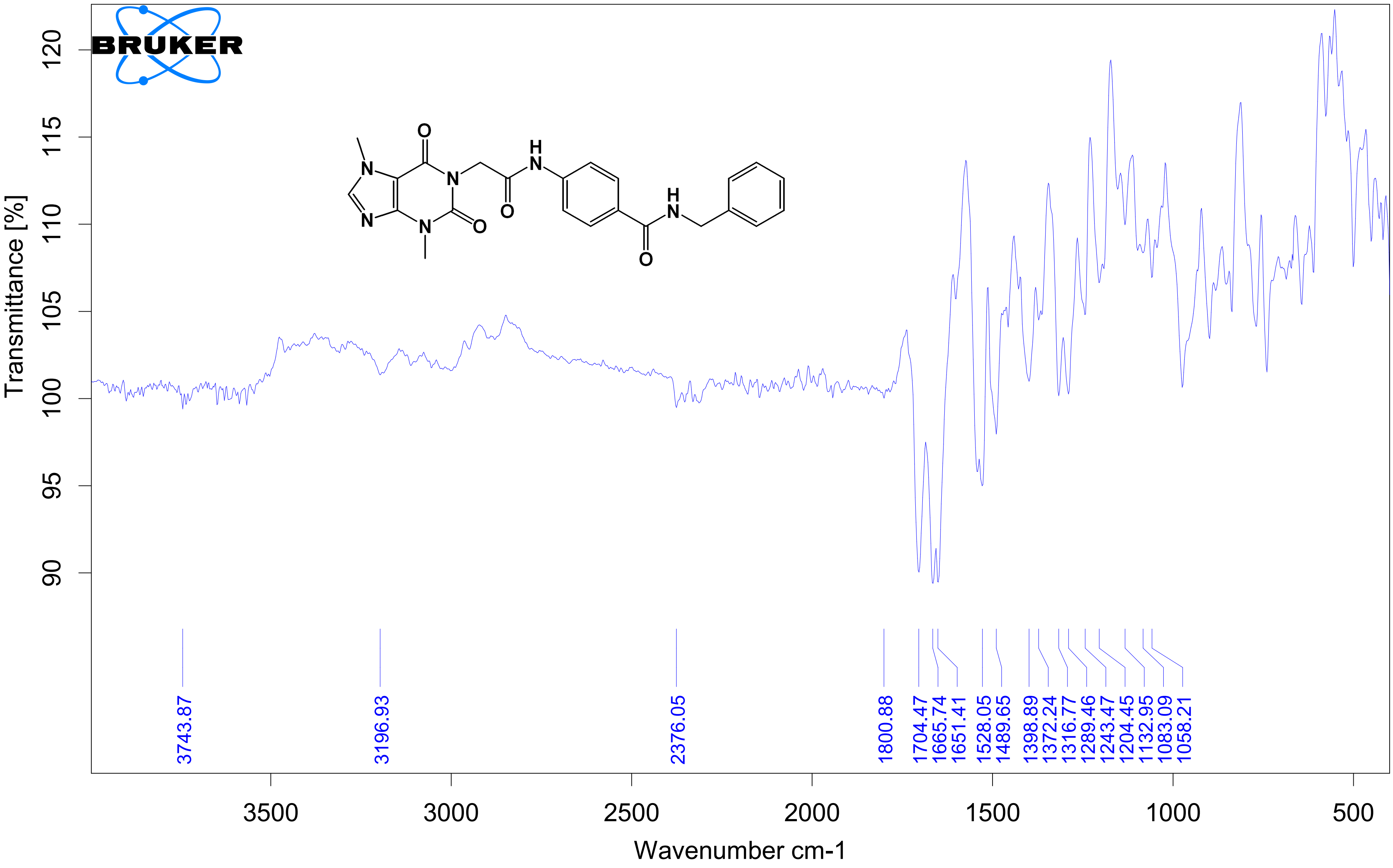

C:\Users\Public\Documents\Bruker\OPUS_8.1.29\DATA\MEAS\XN-3.3	XN-3	Instrument type and / or accessory	2/28/2023

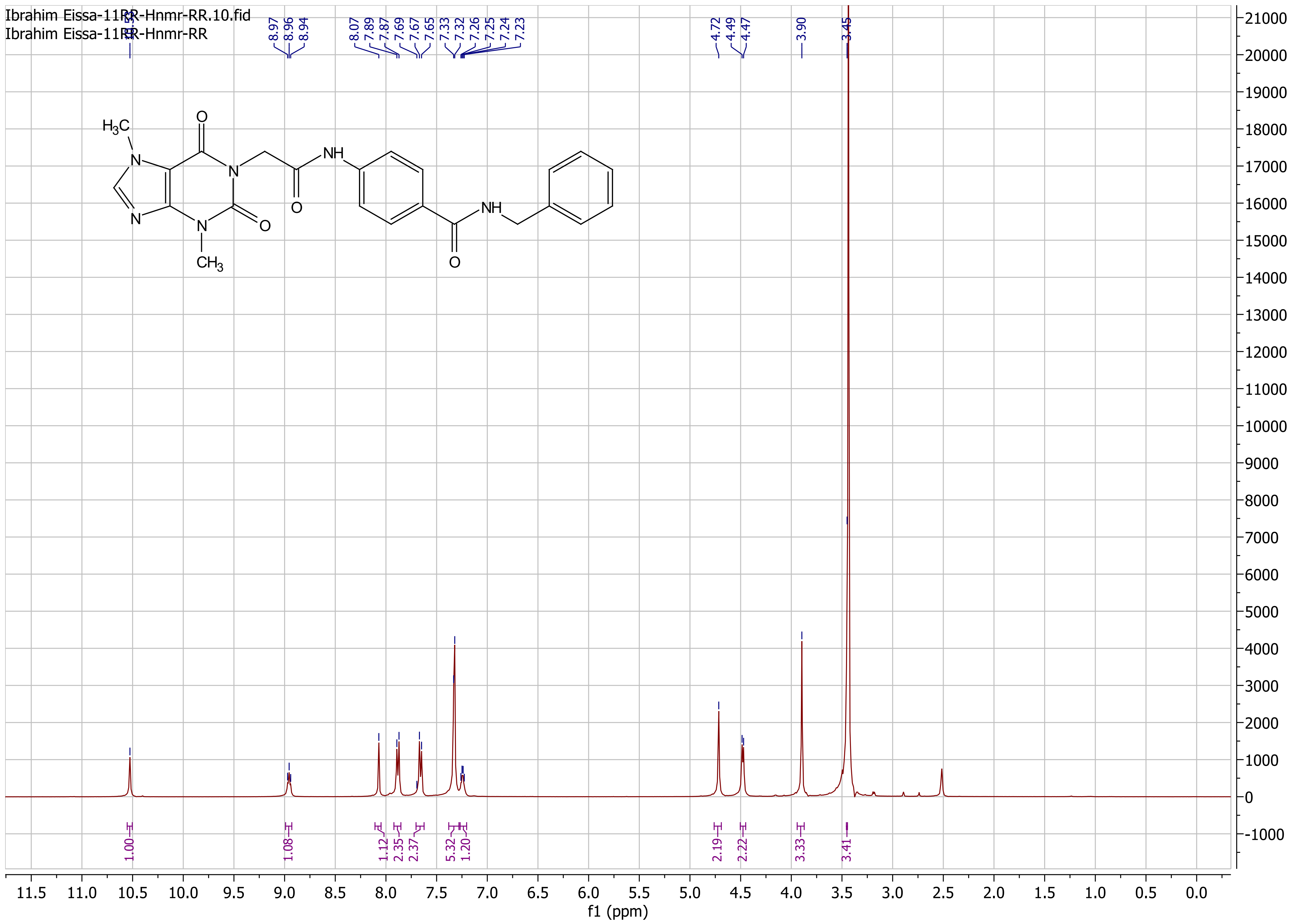

Ibrahim Eissa-11RR-Hnmr-RR.10.fid
Ibrahim Eissa-11RR-Hnmr-RR
8.97 8.96 8.94
8.07 7.89 7.87 7.69 7.67 7.65 7.33 7.32 7.26 7.25 7.24 7.23
4.72 4.49 4.47
3.90
3.45
1.00 1.08 1.12 2.35 2.37 5.32 1.20 2.19 2.22 3.33 3.41
f1 (ppm)

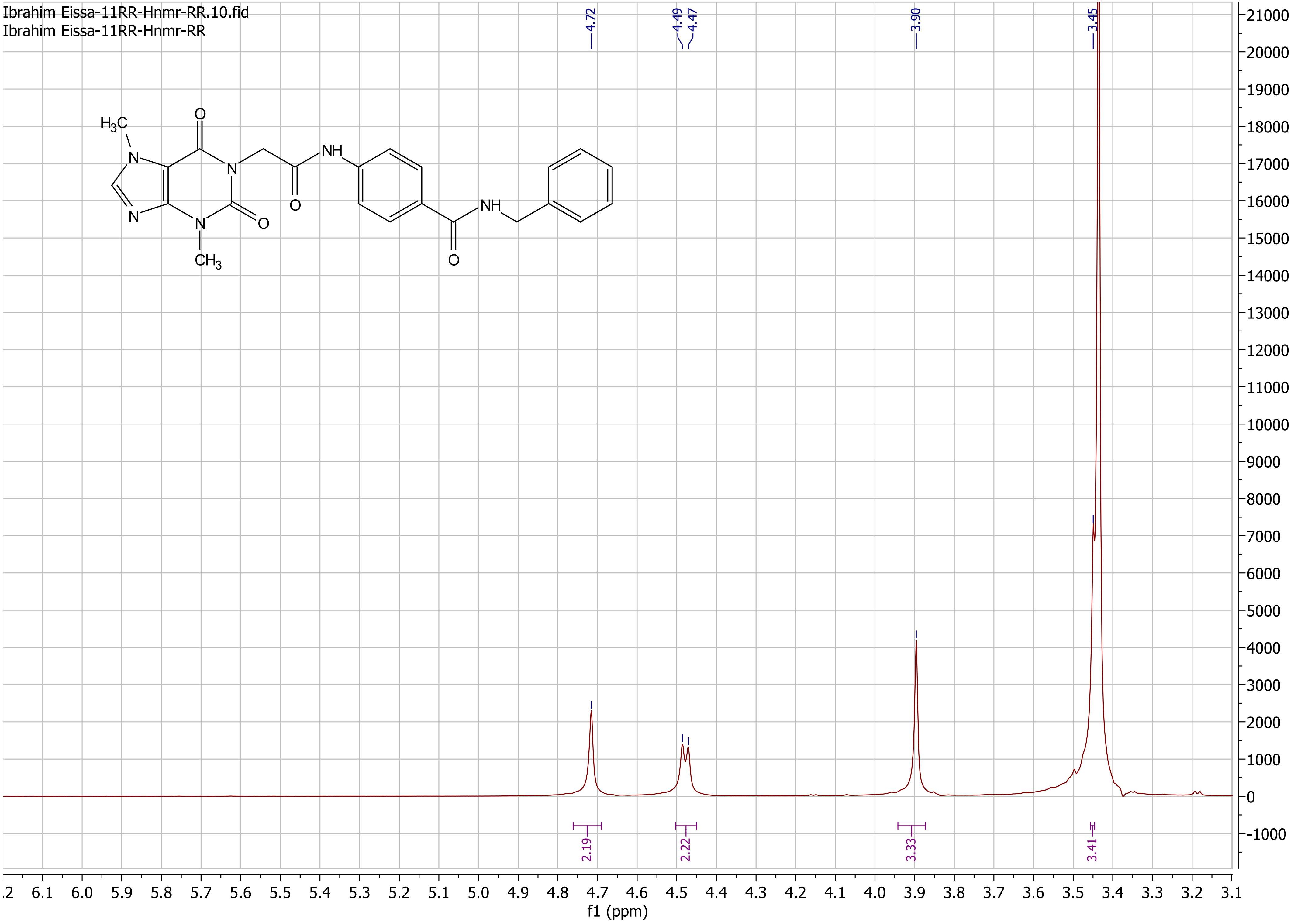
Ibrahim Eissa-11RR-Hnmr-RR.10.fid
Ibrahim Eissa-11RR-Hnmr-RR
4.72
4.49
4.47
3.90
3.45
2.19
2.22
3.33
3.41
f1 (ppm)

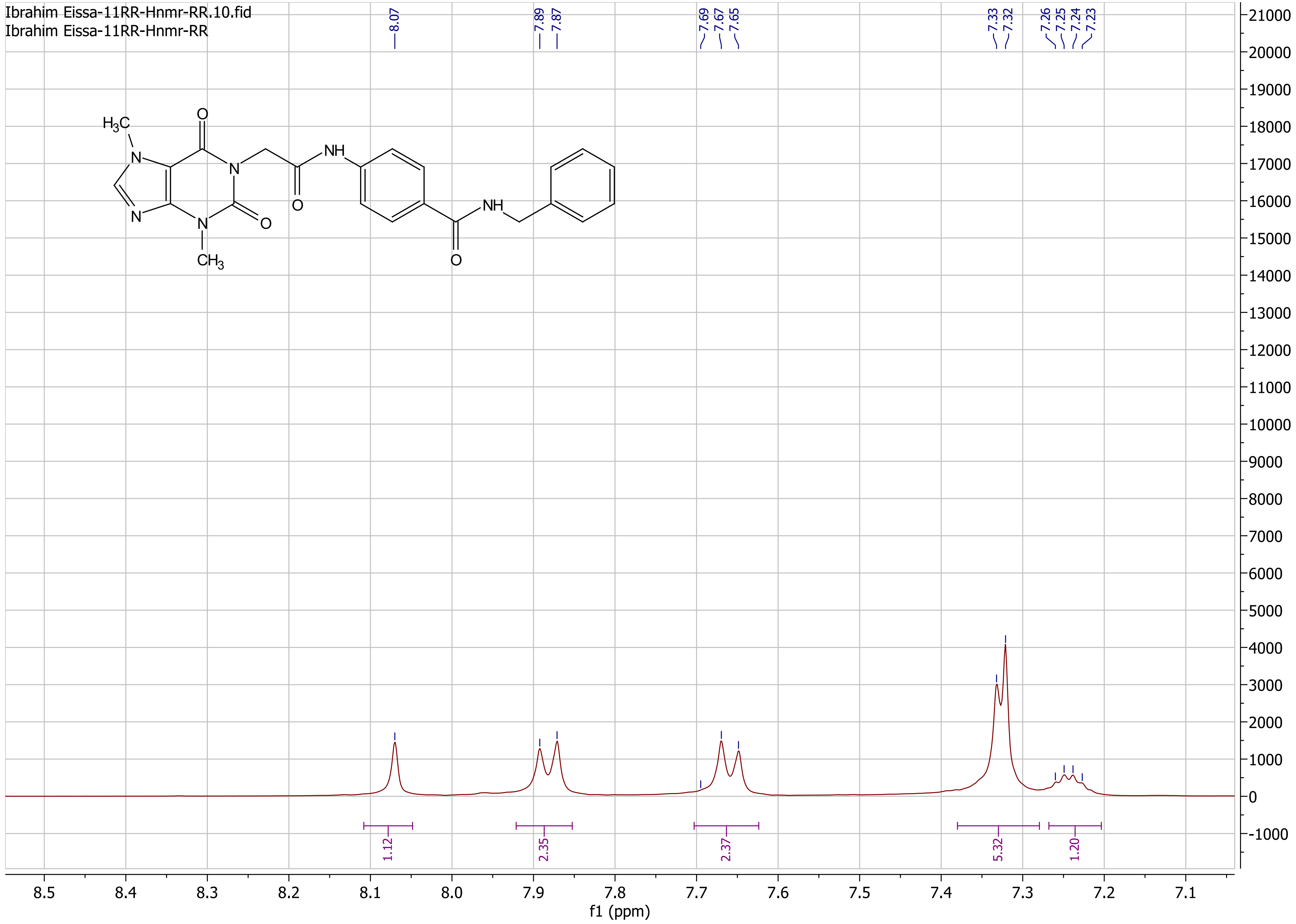
Ibrahim Eissa-11RR-Hnmr-RR.10.fid
Ibrahim Eissa-11RR-Hnmr-RR
8.07
7.89
7.87
7.69
7.67
7.65
7.33
7.32
7.26
7.25
7.24
7.23
1.12
2.35
2.37
5.32
1.20
f1 (ppm)

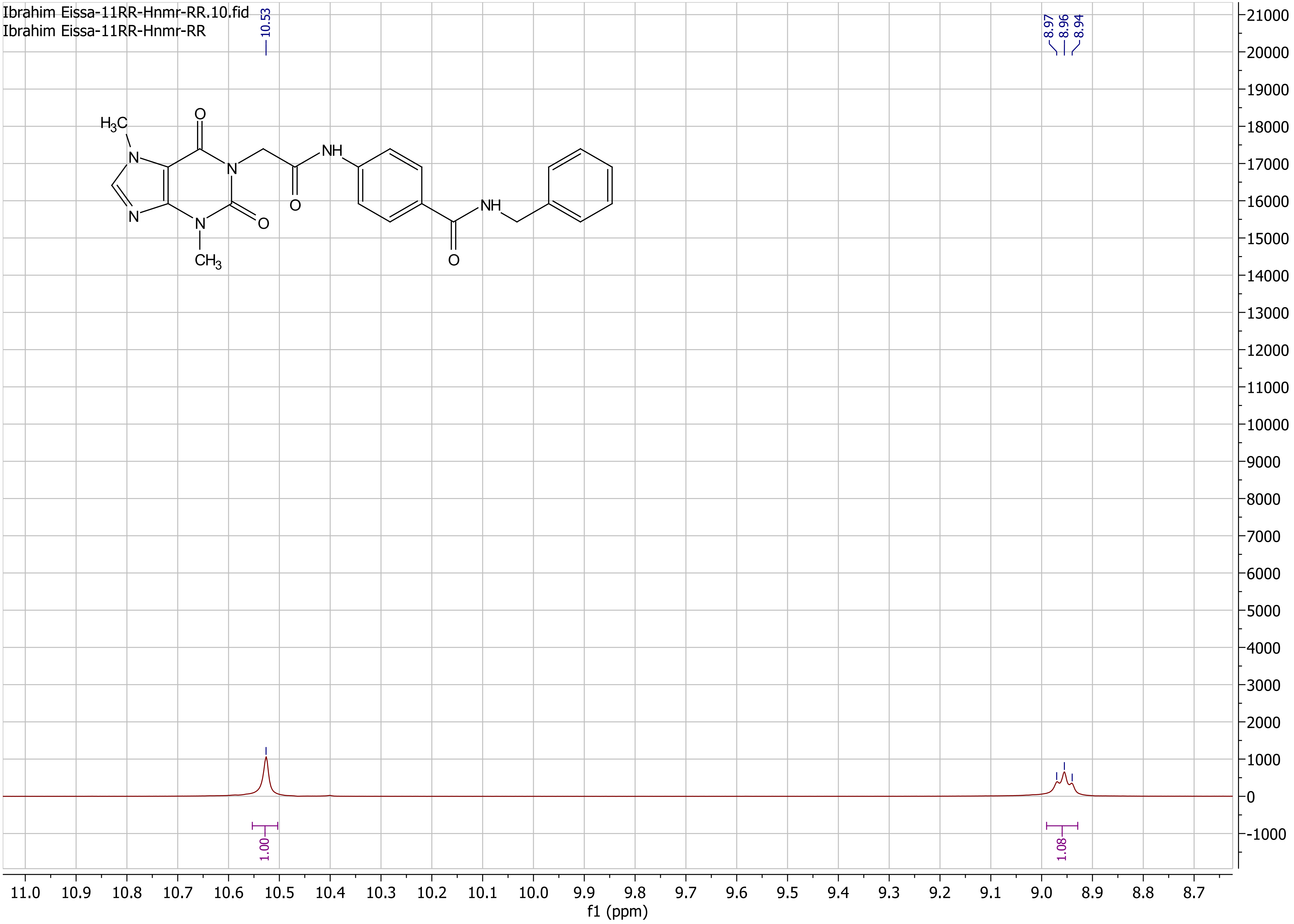
Ibrahim Eissa-11RR-Hnmr-RR.10.fid
Ibrahim Eissa-11RR-Hnmr-RR
10.53
8.97
8.96
8.94
1.00
1.08
f1 (ppm)

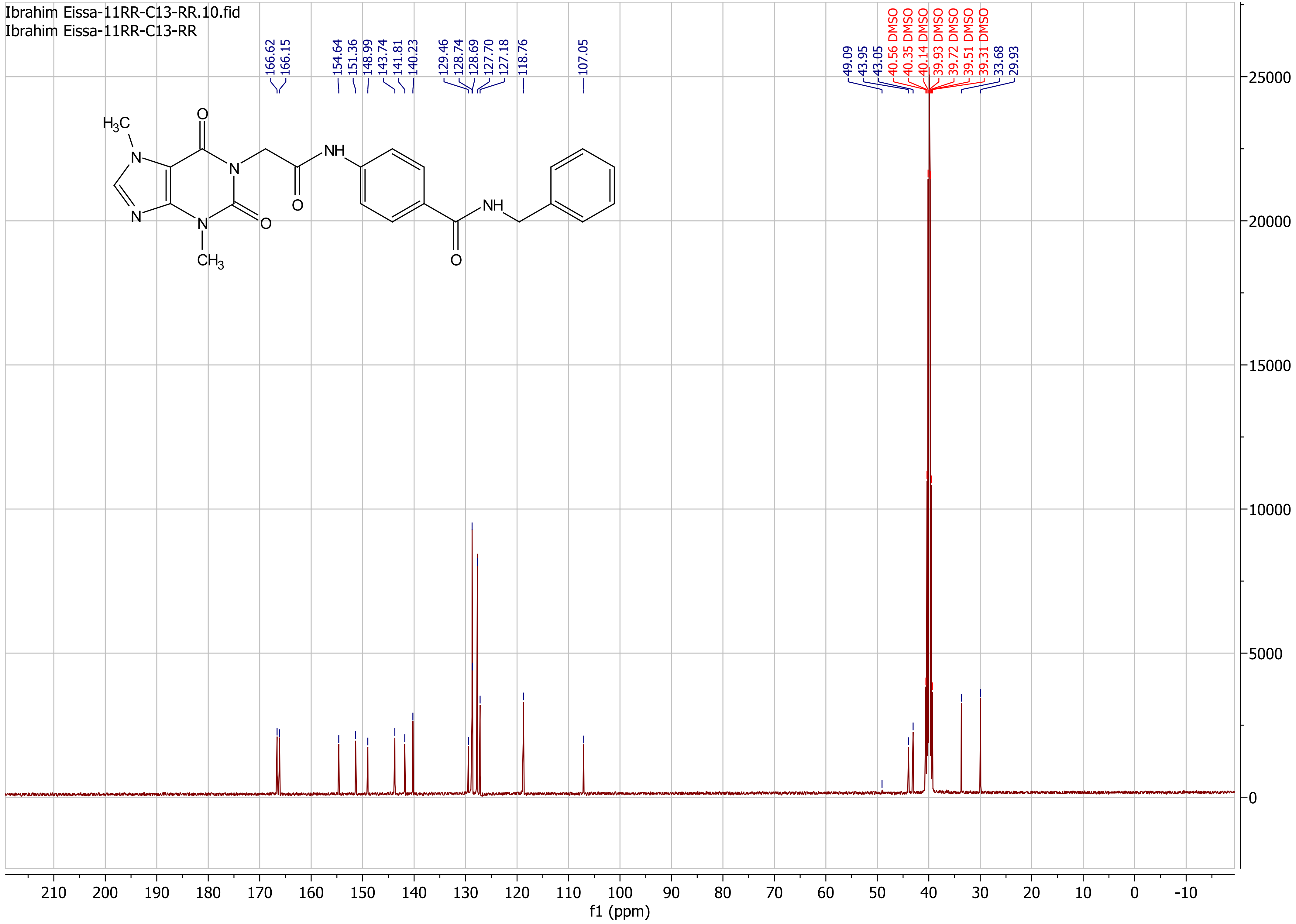
Ibrahim Eissa-11RR-C13-RR.10.fid
Ibrahim Eissa-11RR-C13-RR
166.62
166.15
154.64
151.36
148.99
143.74
141.81
140.23
129.46
128.74
128.69
127.70
127.18
118.76
107.05
49.09
43.95
43.05
40.56 DMSO
40.35 DMSO
40.14 DMSO
39.93 DMSO
39.72 DMSO
39.51 DMSO
39.31 DMSO
33.68
29.93
f1 (ppm)

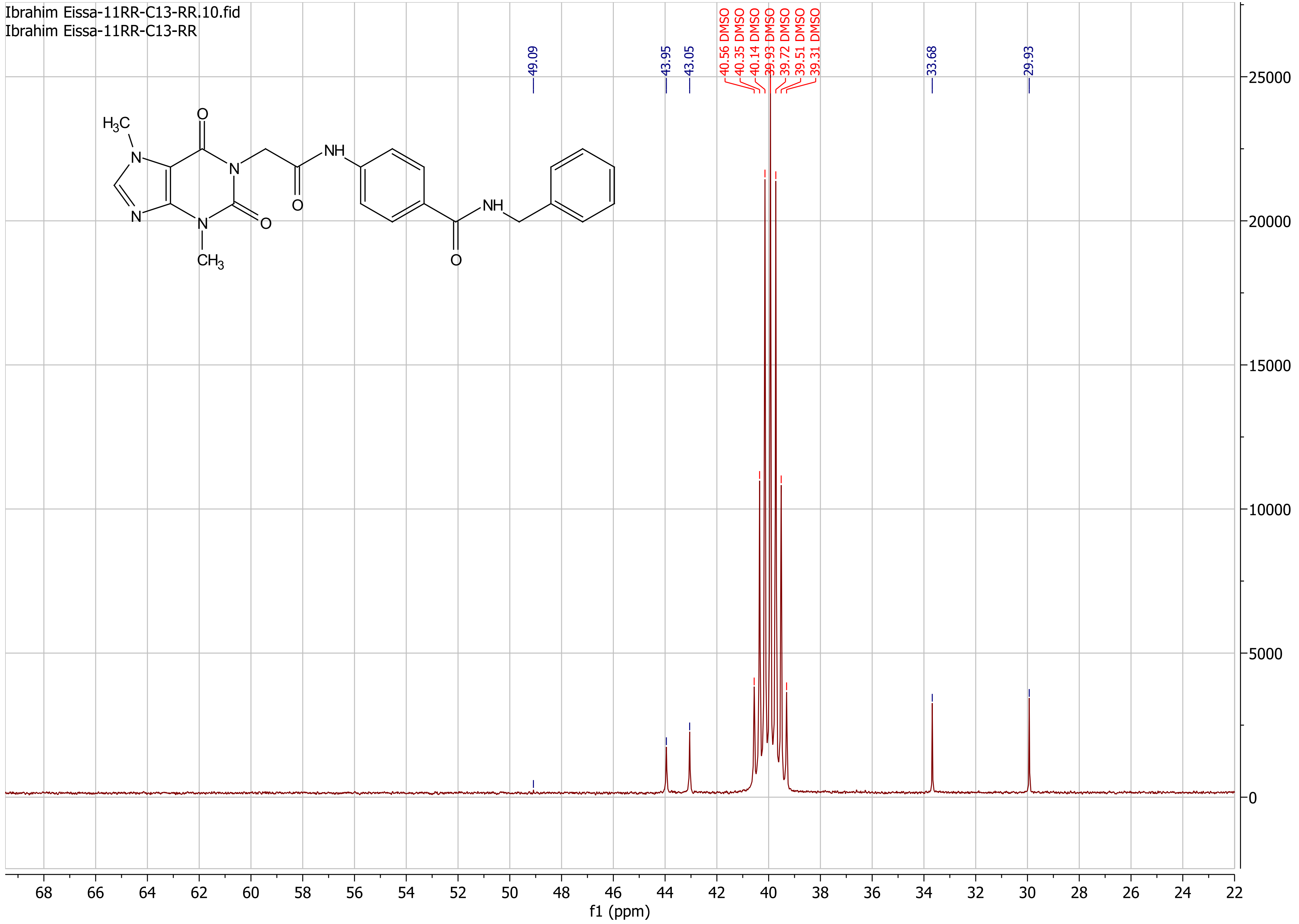
Ibrahim Eissa-11RR-C13-RR.10.fid
Ibrahim Eissa-11RR-C13-RR
49.09
43.95
43.05
40.56 DMSO
40.35 DMSO
40.14 DMSO
39.93 DMSO
39.72 DMSO
39.51 DMSO
39.31 DMSO
33.68
29.93
H3C
N
O
N
NH
O
N
N
O
CH3
NH
O
25000
20000
15000
10000
5000
0
68
66
64
62
60
58
56
54
52
50
48
46
44
42
40
38
36
34
32
30
28
26
24
22
f1 (ppm)

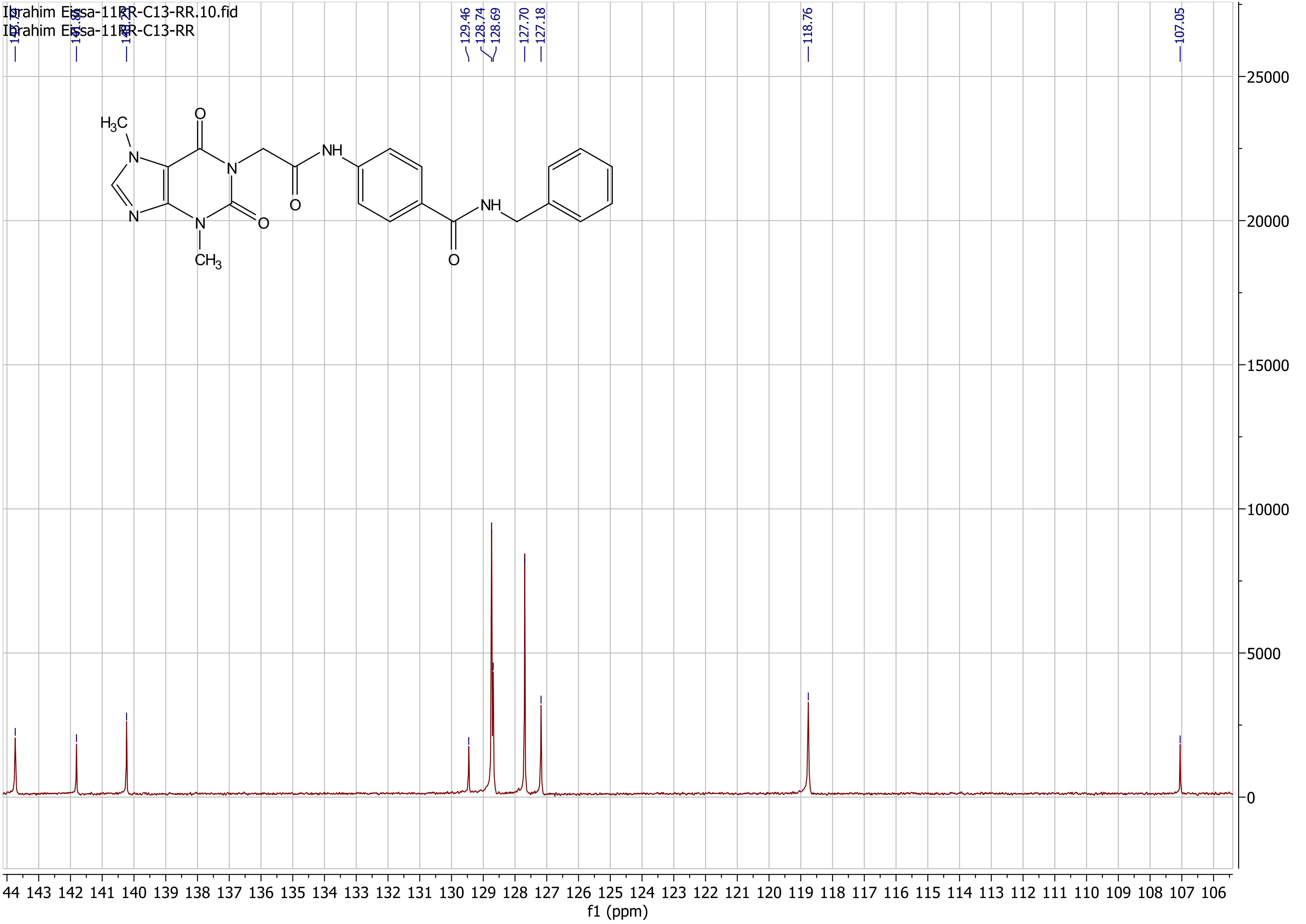
Ibrahim Eissa-11RR-C13-RR.10.fid
Ibrahim Eissa-11RR-C13-RR
129.46
128.74
128.69
127.70
127.18
118.76
107.05
H3C
N
N
N
N
O
O
NH
O
NH
O
CH3
25000
20000
15000
10000
5000
0
44 143 142 141 140 139 138 137 136 135 134 133 132 131 130 129 128 127 126 125 124 123 122 121 120 119 118 117 116 115 114 113 112 111 110 109 108 107 106
f1 (ppm)

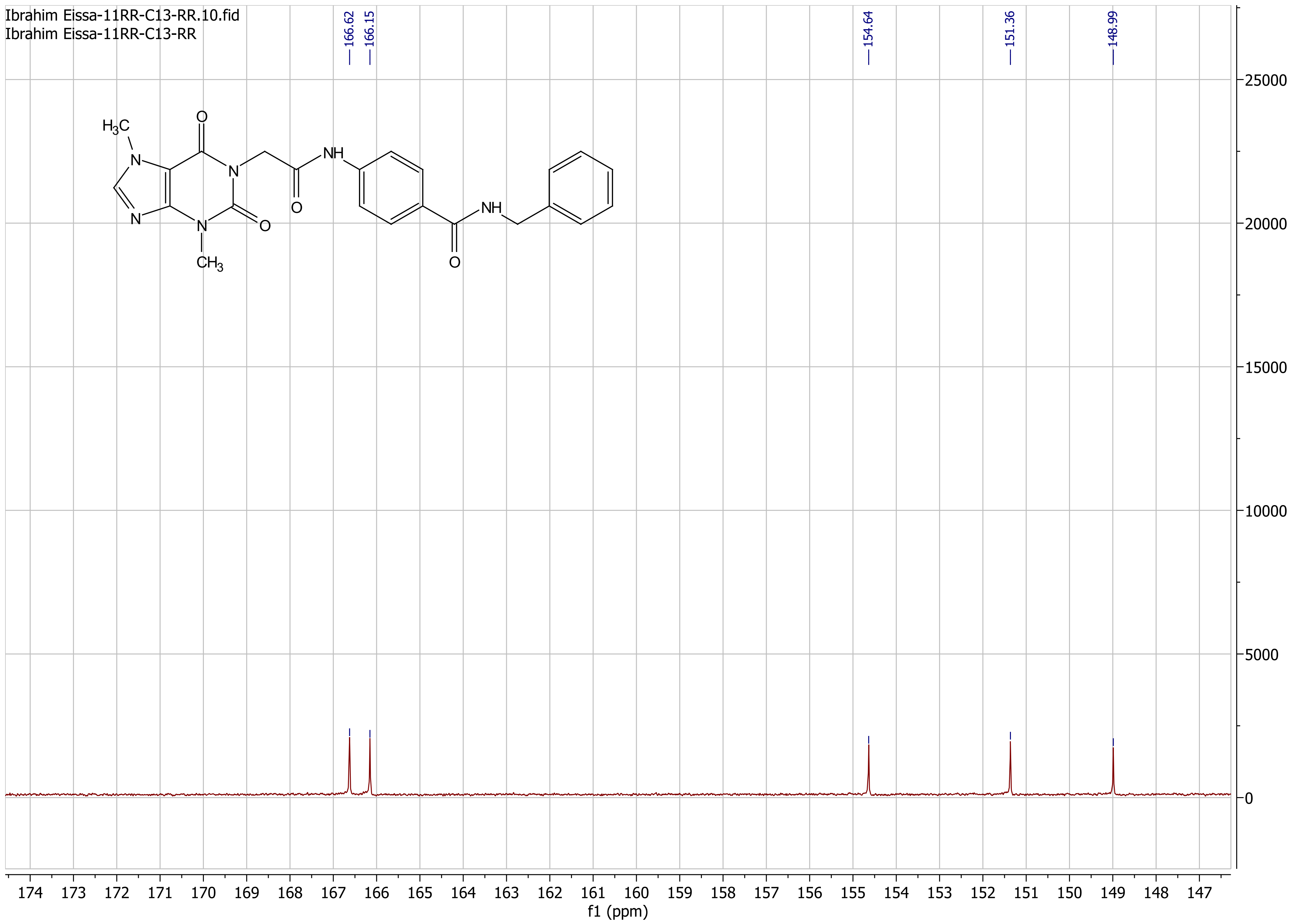

Ibrahim Eissa-11RR-C13-RR.10.fid
Ibrahim Eissa-11RR-C13-RR
166.62
166.15
154.64
151.36
148.99
25000
20000
15000
10000
5000
0
174 173 172 171 170 169 168 167 166 165 164 163 162 161 160 159 158 157 156 155 154 153 152 151 150 149 148 147
f1 (ppm)

Table S1. The QTAIM parameters (a.u.) at bond critical points (BCPs) of **T-1-NBAB**

BCP #	Atoms	(ρ)	(∇2ρ)	K(r)	G(r)	V(r)	H(r)
	1						
1	N1 - C2	0.352502	-1.06857	0.497118	0.229974	-0.72709	-0.49712
2	C2 - N3	0.318258	-0.73347	0.457905	0.274537	-0.73244	-0.45791
3	N1 - C5	0.335587	-1.00561	0.433965	0.182563	-0.61653	-0.43397
4	C5 - C6	0.32155	-0.90715	0.339163	0.112376	-0.45154	-0.33916
5	N4 - C5	0.307991	-0.87284	0.413154	0.194945	-0.6081	-0.41315
6	N3 - C6	0.294333	-0.69086	0.397483	0.224769	-0.62225	-0.39748
7	C6 - C7	0.2936	-0.79741	0.281677	0.082325	-0.364	-0.28168
8	C7 - N8	0.279722	-0.74691	0.328472	0.141744	-0.47022	-0.32847
9	N4 - C9	0.307609	-0.89567	0.391645	0.167728	-0.55937	-0.39165
10	N14 - H36	0.342557	-1.66365	0.470344	0.054431	-0.52478	-0.47034
11	N8 - C9	0.287688	-0.78716	0.334362	0.137571	-0.47193	-0.33436
12	C7 - O10	0.406059	-0.33209	0.682067	0.599046	-1.28111	-0.68207
13	C9 - O11	0.41344	-0.35262	0.701718	0.613564	-1.31528	-0.70172
14	N8 - C12	0.249249	-0.60247	0.289303	0.138685	-0.42799	-0.2893
15	C12 - C13	0.24663	-0.5707	0.197446	0.054771	-0.25222	-0.19745
16	N8 - H36	0.016282	0.067149	-0.00267	0.014118	-0.01145	0.00267
17	N14 - C15	0.284469	-0.76833	0.368401	0.176318	-0.54472	-0.3684
18	C12 - H34	0.284633	-0.98694	0.282392	0.035657	-0.31805	-0.28239
19	C13 - N14	0.31066	-0.86225	0.423002	0.207439	-0.63044	-0.423
20	C13 - O16	0.409063	-0.25462	0.689543	0.625887	-1.31543	-0.68954
21	O16 - H46	0.017506	0.064124	-0.00217	0.013857	-0.01168	0.002173
22	N4 - C17	0.245557	-0.57337	0.292624	0.149283	-0.44191	-0.29262
23	N3 - C18	0.249585	-0.59598	0.302016	0.15302	-0.45504	-0.30202
24	C15 - C19	0.305081	-0.842	0.307787	0.097285	-0.40507	-0.30779
25	C19 - C20	0.313267	-0.88167	0.325107	0.10469	-0.4298	-0.32511
26	C20 - H44	0.284954	-0.99431	0.283974	0.035396	-0.31937	-0.28397
27	C21 - C22	0.305027	-0.83414	0.307754	0.099219	-0.40697	-0.30775
28	C20 - C21	0.30575	-0.84543	0.308815	0.097458	-0.40627	-0.30882
29	C15 - C23	0.305212	-0.84532	0.310936	0.099605	-0.41054	-0.31094
30	C23 - H46	0.28836	-1.0171	0.289612	0.035337	-0.32495	-0.28961
31	C22 - C23	0.309085	-0.85935	0.316382	0.101544	-0.41793	-0.31638
32	C24 - N25	0.306262	-0.8566	0.411405	0.197257	-0.60866	-0.41141
33	C21 - C24	0.260265	-0.63678	0.219994	0.0608	-0.28079	-0.21999
34	C24 - O26	0.404153	-0.29762	0.677597	0.603191	-1.28079	-0.6776
35	O26 - H52	0.017555	0.064326	-0.00215	0.013931	-0.01178	0.002151
36	C31 - C32	0.308172	-0.8564	0.314326	0.100225	-0.41455	-0.31433
37	N25 - C27	0.281812	-0.75628	0.36232	0.173249	-0.53557	-0.36232
38	C27 - C32	0.304445	-0.83975	0.309625	0.099688	-0.40931	-0.30963
39	C30 - C31	0.309524	-0.86613	0.316922	0.100389	-0.41731	-0.31692
40	C31 - H51	0.28186	-0.97105	0.280925	0.038164	-0.31909	-0.28093
41	C27 - C28	0.305077	-0.83878	0.307587	0.097892	-0.40548	-0.30759
42	C29 - C30	0.308287	-0.85996	0.314688	0.099699	-0.41439	-0.31469

BCP #	Atoms	(ρ)	(∇2ρ)	K(r)	G(r)	V(r)	H(r)
43	N25 - H47	0.343733	-1.65232	0.46856	0.05548	-0.52404	-0.46856
44	C28 - C29	0.310199	-0.86528	0.318838	0.102517	-0.42136	-0.31884
45	C30 - H50	0.281235	-0.96491	0.280346	0.039119	-0.31947	-0.28035
46	C32 - H52	0.288207	-1.01504	0.289444	0.035686	-0.32513	-0.28944
47	C2 - H33	0.288383	-1.02565	0.288499	0.032088	-0.32059	-0.2885
48	C12 - H35	0.283911	-0.98203	0.281339	0.035833	-0.31717	-0.28134
49	C19 - H43	0.278959	-0.94765	0.277161	0.04025	-0.31741	-0.27716
50	C17 - H37	0.280464	-0.95658	0.276664	0.03752	-0.31418	-0.27666
51	C17 - H38	0.281795	-0.96642	0.278712	0.037108	-0.31582	-0.27871
52	C17 - H39	0.284489	-0.98637	0.28296	0.036367	-0.31933	-0.28296
53	C18 - H40	0.280775	-0.96055	0.278317	0.038179	-0.3165	-0.27832
54	C18 - H41	0.282	-0.96878	0.278759	0.036566	-0.31533	-0.27876
55	C18 - H42	0.282274	-0.97094	0.279144	0.036409	-0.31555	-0.27914
56	C22 - H47	0.012453	0.054665	-0.0027	0.010966	-0.00827	0.0027
57	C22 - H45	0.281202	-0.96358	0.280544	0.039648	-0.32019	-0.28054
58	C28 - H48	0.278282	-0.94228	0.276518	0.040948	-0.31747	-0.27652
59	C29 - H49	0.281556	-0.96875	0.280667	0.038479	-0.31915	-0.28067

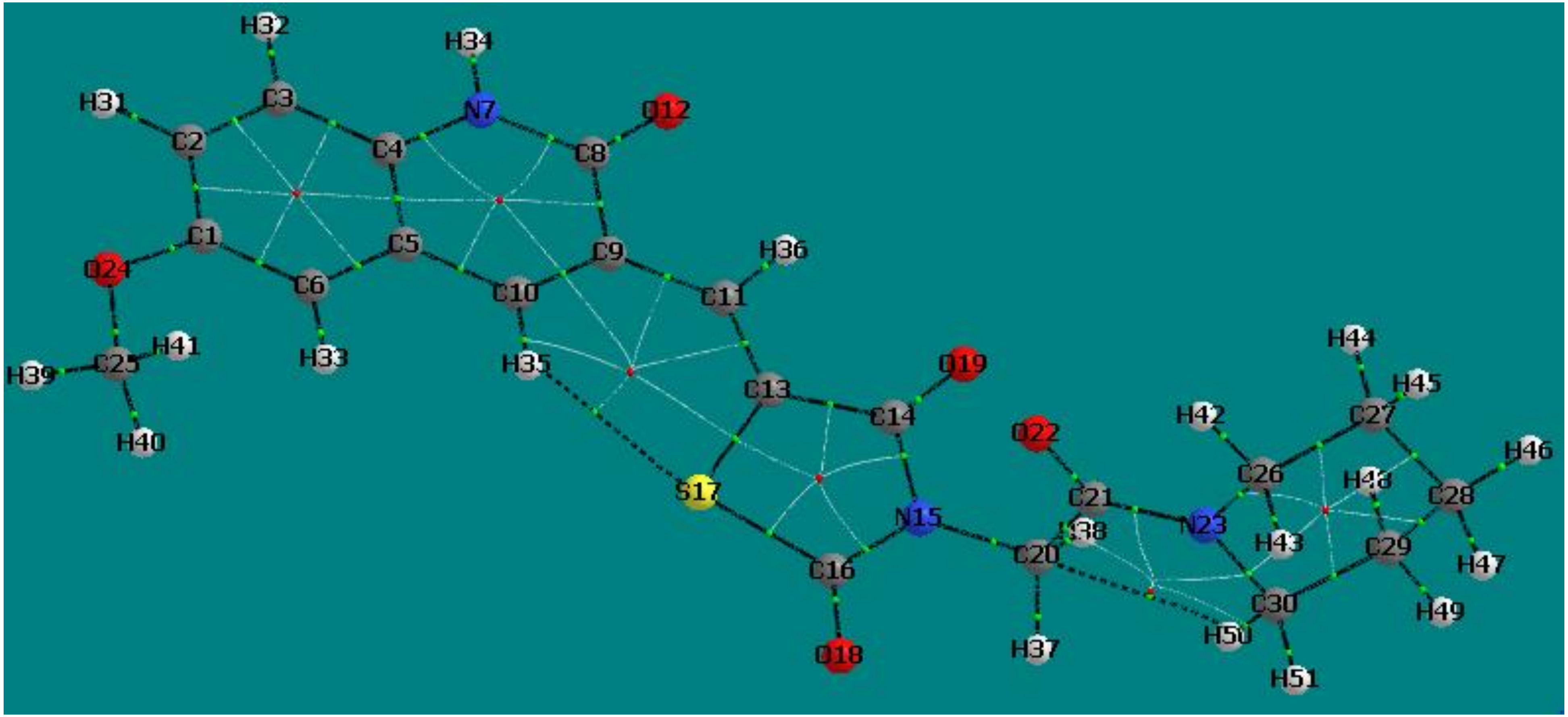

Figure S1. QTAIM analysis: molecular graph showing the bonding critical points (BCP) for **T-1-NBAB**

Toxicity Report

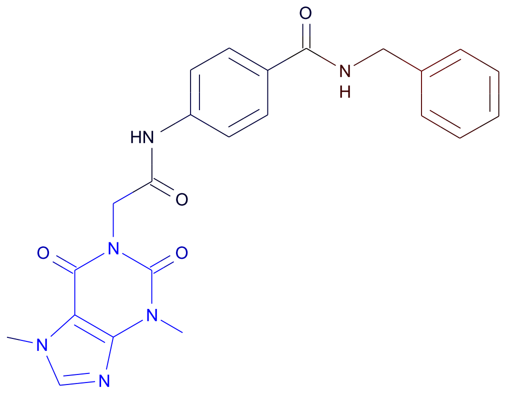

$C_{23}H_{22}N_6O_4$
Molecular Weight: 446.45858
ALogP: 1.328
Rotatable Bonds: 6
Acceptors: 5
Donors: 2

Model Prediction

Prediction: Non-Mutagen
Probability: 0.357
Enrichment: 0.64
Bayesian Score: -10.6
Mahalanobis Distance: 12.2
Mahalanobis Distance p-value: 0.000296

Prediction: Positive if the Bayesian score is above the estimated best cutoff value from minimizing the false positive and false negative rate.
Probability: The esimated probability that the sample is in the positive category. This assumes that the Bayesian score follows a normal distribution and is different from the prediction using a cutoff.
Enrichment: An estimate of enrichment, that is, the increased likelihood (versus random) of this sample being in the category.
Bayesian Score: The standard Laplacian-modified Bayesian score.
Mahalanobis Distance: The Mahalanobis distance (MD) is the distance to the center of the training data. The larger the MD, the less trustworthy the prediction.
Mahalanobis Distance p-value: The p-value gives the fraction of training data with an MD greater than or equal to the one for the given sample, assuming normally distributed data. The smaller the p-value, the less trustworthy the prediciton. For highly non-normal X properties (e.g., fingerprints), the MD p-value is wildly inaccurate.

Structural Similar Compounds

Name	67450-45-7	Delavirdine	132-98-9
Structure	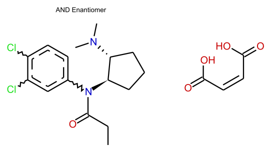	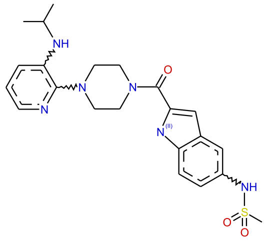	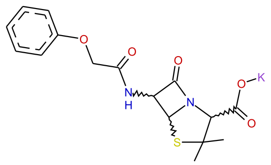
Actual Endpoint	Non-Mutagen	Non-Mutagen	Non-Mutagen
Predicted Endpoint	Non-Mutagen	Non-Mutagen	Non-Mutagen
Distance	0.548	0.614	0.622
Reference	Kazius et. al., J. Med. Chem. (2005) 48, 312-320	Contrera, J.F., Matthews, E.J., Kruhlak, N.L., and Benz, R.D., Regulatory Toxicology and Pharmacology 2005, 313-323.	Helma, C., Cramer, T., Kramer, S., and De Raedt, L., J. Chem. Inf. Comput. Sci., 2004, pp. 1402-1411

Model Applicability

Unknown features are fingerprint features in the query molecule, but not found or appearing too infrequently in the training set.

1. All properties and OPS components are within expected ranges.

Feature Contribution

Top features for positive contribution

Fingerprint	Bit/Smiles	Feature Structure	Score	Mutagen in training set
SCFP_12	1575781215	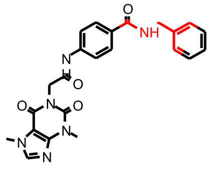 [*]C(=[*])NC[c](:[cH]:[*]):[cH]:[*]	0.517	16 out of 16

SCFP_12	-1211234921	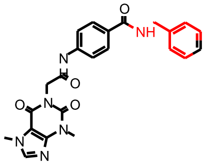 [*]NC[c]1:[cH]:[cH]:[*]:[cH]:[cH]:1	0.514	15 out of 15
SCFP_12	-1356986522	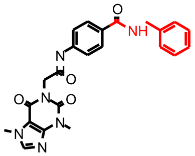 [*]C(=[*])NC[c]1:[cH] :[cH]:[cH]:[cH]:[cH] :1	0.499	11 out of 11
**Top Features for negative contribution**				
**Fingerprint**	**Bit/Smiles**	**Feature Structure**	**Score**	**Mutagen in training set**
SCFP_12	1205795299	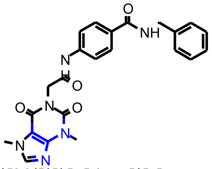 [*]N([*])[c]1:n:[*]:[*]:[c]:1[*]	-1.22	2 out of 16
SCFP_12	1731225349	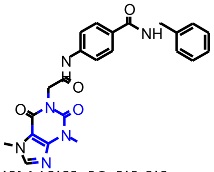 [*]N1[*][c]2:[*]:[*]: n:[c]:2N(C)C1=O	-1.19	0 out of 4
SCFP_12	1445006032	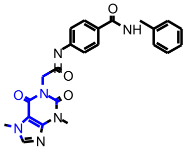 [*]CN1C(=[*])[*][c]2: [*]:[*]:n([*]):[c]:2 C1=O	-1.19	0 out of 4

Sorafenib

TOPKAT_Ames_Mutagenicity

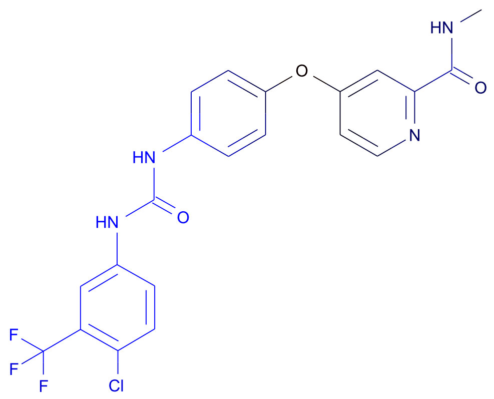

$C_{21}H_{16}ClF_3N_4O_3$
Molecular Weight: 464.82494
ALogP: 4.175
Rotatable Bonds: 6
Acceptors: 4
Donors: 3

Structural Similar Compounds

Name	GLYBURIDE	38914-96-4	93957-54-1
Structure	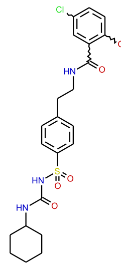	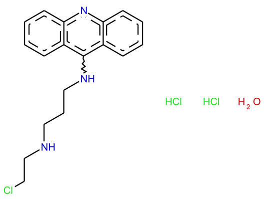	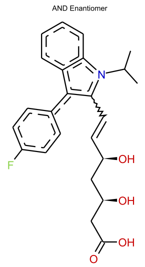
Actual Endpoint	Non-Mutagen	Mutagen	Non-Mutagen
Predicted Endpoint	Non-Mutagen	Mutagen	Non-Mutagen
Distance	0.590	0.592	0.600
Reference	PDR 1994	Kazius et. al., J. Med. Chem. (2005) 48, 312-320	US Environmental Protection Agency at http://www.epa.gov/NCCT/dsstox/sdf_isscan_external.html

Model Prediction

Prediction: Non-Mutagen
Probability: 0.0531
Enrichment: 0.0951
Bayesian Score: -19.7
Mahalanobis Distance: 13.1
Mahalanobis Distance p-value: 2.73e-006

Prediction: Positive if the Bayesian score is above the estimated best cutoff value from minimizing the false positive and false negative rate.
Probability: The esimated probability that the sample is in the positive category. This assumes that the Bayesian score follows a normal distribution and is different from the prediction using a cutoff.
Enrichment: An estimate of enrichment, that is, the increased likelihood (versus random) of this sample being in the category.
Bayesian Score: The standard Laplacian-modified Bayesian score.
Mahalanobis Distance: The Mahalanobis distance (MD) is the distance to the center of the training data. The larger the MD, the less trustworthy the prediction.
Mahalanobis Distance p-value: The p-value gives the fraction of training data with an MD greater than or equal to the one for the given sample, assuming normally distributed data. The smaller the p-value, the less trustworthy the prediciton. For highly non-normal X properties (e.g., fingerprints), the MD p-value is wildly inaccurate.

Model Applicability

Unknown features are fingerprint features in the query molecule, but not found or appearing too infrequently in the training set.

1. All properties and OPS components are within expected ranges.

Feature Contribution

Top features for positive contribution

Fingerprint	Bit/Smiles	Feature Structure	Score	Mutagen in training set
SCFP_12	-347281112	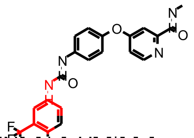 [*]N[c]1:[cH]:[*]:[c]([*]):[c](:[cH]:1)C([*])([*])[*]	0.337	18 out of 22

SCFP_12	1208843554	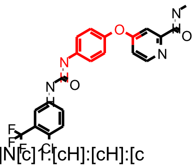 [*]N[c]1:[cH]:[cH]:[c](O[c](:[*]):[*]):[c H]:[cH]:1	0.337	6 out of 7
SCFP_12	-1943080297	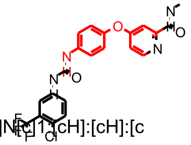 [*]N[c]1:[cH]:[cH]:[c](O[c]2:[cH]:[cH]:[*]:[c]([*]):[cH]:2):[cH]:[cH]:1	0.304	5 out of 6

Top Features for negative contribution

Fingerprint	Bit/Smiles	Feature Structure	Score	Mutagen in training set
SCFP_12	816802409	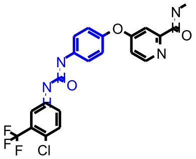 [*]NC(=O)N[c]1:[cH]:[cH]:[c]([*]):[cH]:[c H]:1	-1.82	0 out of 9
SCFP_12	-300280774	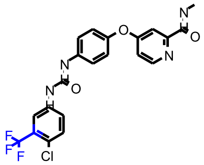 [*]:[c](:[*])C(F)(F)F	-1.51	3 out of 30
SCFP_12	-1903175541	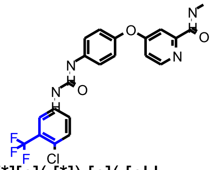 [*][c](:[*]):[c](:[cH]:[*])C(F)(F)F	-1.51	3 out of 30

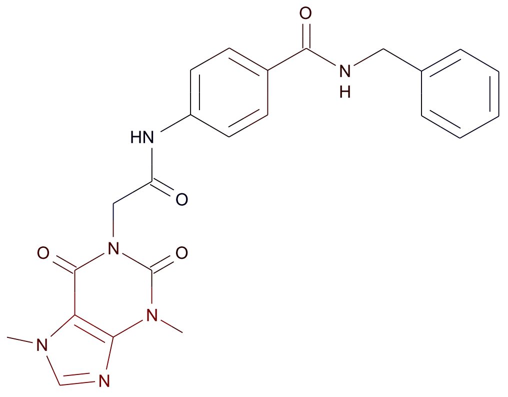

$C_{23}H_{22}N_6O_4$

Molecular Weight: 446.45858

ALogP: 1.328

Rotatable Bonds: 6

Acceptors: 5

Donors: 2

Model Prediction

Prediction: Toxic

Probability: 0.642

Enrichment: 1.22

Bayesian Score: 2.43

Mahalanobis Distance: 8.63

Mahalanobis Distance p-value: 0.311

Prediction: Positive if the Bayesian score is above the estimated best cutoff value from minimizing the false positive and false negative rate.
Probability: The esimated probability that the sample is in the positive category. This assumes that the Bayesian score follows a normal distribution and is different from the prediction using a cutoff.
Enrichment: An estimate of enrichment, that is, the increased likelihood (versus random) of this sample being in the category.
Bayesian Score: The standard Laplacian-modified Bayesian score.
Mahalanobis Distance: The Mahalanobis distance (MD) is the distance to the center of the training data. The larger the MD, the less trustworthy the prediction.
Mahalanobis Distance p-value: The p-value gives the fraction of training data with an MD greater than or equal to the one for the given sample, assuming normally distributed data. The smaller the p-value, the less trustworthy the prediciton. For highly non-normal X properties (e.g., fingerprints), the MD p-value is wildly inaccurate.

Structural Similar Compounds

Name	Clebopride Malate	Citreoviridin	Sultopride .HCl (Free base form)
Structure	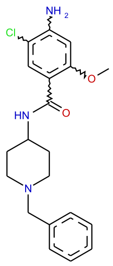	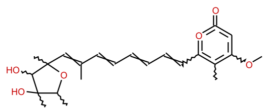	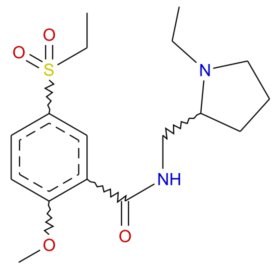
Actual Endpoint	Non-Toxic	Toxic	Toxic
Predicted Endpoint	Non-Toxic	Toxic	Toxic
Distance	0.637	0.644	0.651
Reference	Kiso to Rinsho 16:5649-5660; 1982	Food Chem Toxicol 24(12):1315-20; 1986	Oyo Yakuri-Pharmacometrics 28:663-674; 1984

Model Applicability

Unknown features are fingerprint features in the query molecule, but not found or appearing too infrequently in the training set.

1. All properties and OPS components are within expected ranges.

Feature Contribution

Top features for positive contribution				
Fingerprint	Bit/Smiles	Feature Structure	Score	Toxic in training set
SCFP_6	282594097	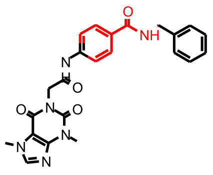 [*]NC(=O)[c]1:[cH]:[cH]:[*]:[cH]:[cH]:1	0.441	3 out of 3

SCFP_6	1257084377	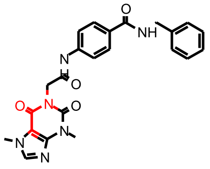 [*]N([*])C(=O)[c](:[*]):[*]	0.362	14 out of 18
SCFP_6	-1181430618	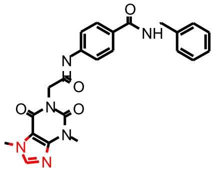 [*]n1:[*]:[*]:n:[cH]:1	0.298	6 out of 8

Top Features for negative contribution

Fingerprint	Bit/Smiles	Feature Structure	Score	Toxic in training set
SCFP_6	136358998	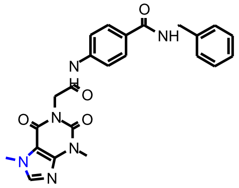 [*]:n(:[*])C	-0.55	2 out of 8
SCFP_6	399659969	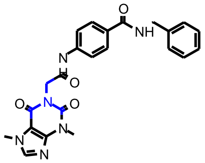 [*]CN(C(=[*])[*])C(=[*])[*]	-0.526	3 out of 11
SCFP_6	2097618059	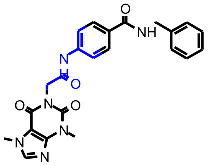 [*]CC(=O)N[c](:[cH]:[*]):[cH]:[*]	-0.422	0 out of 1

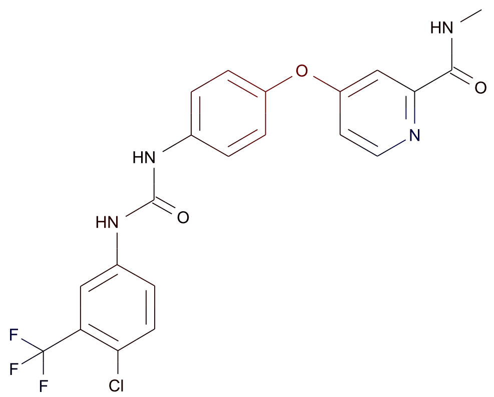

$C_{21}H_{16}ClF_3N_4O_3$
Molecular Weight: 464.82494
ALogP: 4.175
Rotatable Bonds: 6
Acceptors: 4
Donors: 3

Model Prediction

Prediction: Toxic
Probability: 0.592
Enrichment: 1.13
Bayesian Score: 1.15
Mahalanobis Distance: 12.6
Mahalanobis Distance p-value: 2.07e-006

Prediction: Positive if the Bayesian score is above the estimated best cutoff value from minimizing the false positive and false negative rate.
Probability: The esimated probability that the sample is in the positive category. This assumes that the Bayesian score follows a normal distribution and is different from the prediction using a cutoff.
Enrichment: An estimate of enrichment, that is, the increased likelihood (versus random) of this sample being in the category.
Bayesian Score: The standard Laplacian-modified Bayesian score.
Mahalanobis Distance: The Mahalanobis distance (MD) is the distance to the center of the training data. The larger the MD, the less trustworthy the prediction.
Mahalanobis Distance p-value: The p-value gives the fraction of training data with an MD greater than or equal to the one for the given sample, assuming normally distributed data. The smaller the p-value, the less trustworthy the prediciton. For highly non-normal X properties (e.g., fingerprints), the MD p-value is wildly inaccurate.

Structural Similar Compounds

Name	Chenodiol	Amsacrine	Ochratoxin a
Structure	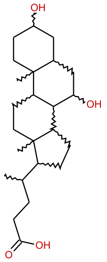	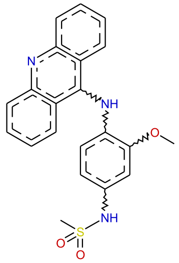	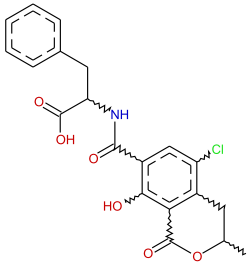
Actual Endpoint	Toxic	Toxic	Toxic
Predicted Endpoint	Toxic	Toxic	Toxic
Distance	0.631	0.637	0.644
Reference	Arch Int Pharm 246:149-158; 1980	Fundam Appl Toxicol 7(2):214-20; 1986	Toxicol Appl Pharmacol 37(2):331-8; 1976

Model Applicability

Unknown features are fingerprint features in the query molecule, but not found or appearing too infrequently in the training set.

1. All properties and OPS components are within expected ranges.

Feature Contribution

Top features for positive contribution

Fingerprint	Bit/Smiles	Feature Structure	Score	Toxic in training set
SCFP_6	1559190850	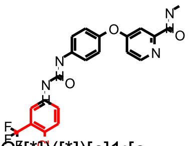 [*]C([*])([*])[c]1:[cH]:[*]:[cH]:[cH]:[c]:1Cl	0.441	3 out of 3

SCFP_6	-488587948	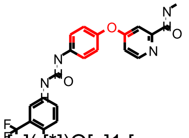 [*]:[c](:[*])O[c]1:[cH]:[cH]:[*]:[cH]:[cH]:1	0.381	2 out of 2
SCFP_6	-975241316	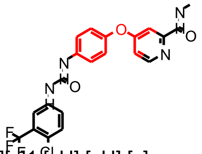 [*][c]1:[cH]:[cH]:[c](O[c](:[cH]:[*]):[cH]:[*]):[cH]:[cH]:1	0.381	2 out of 2

Top Features for negative contribution

Fingerprint	Bit/Smiles	Feature Structure	Score	Toxic in training set
SCFP_6	-1794974220	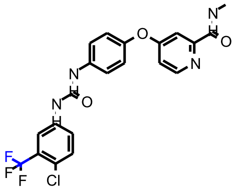 [*]C([*])([*])F	-0.55	2 out of 8
SCFP_6	-937094999	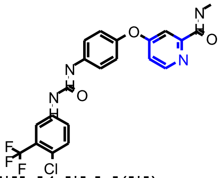 [*][c]1:[*]:[c]([*]):n:[cH]:[cH]:1	-0.358	3 out of 9
SCFP_6	-496201075	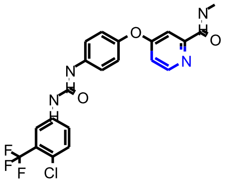 [*]:[cH]:[cH]:n:[*]	-0.289	8 out of 21

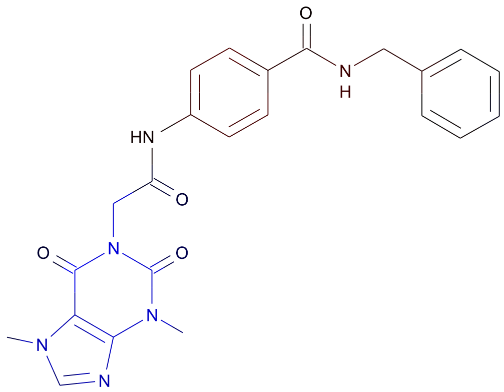

$C_{23}H_{22}N_6O_4$

Molecular Weight: 446.45858

ALogP: 1.328

Rotatable Bonds: 6

Acceptors: 5

Donors: 2

Model Prediction

Prediction: Non-Carcinogen

Probability: 0.206

Enrichment: 0.641

Bayesian Score: -5.5

Mahalanobis Distance: 13.6

Mahalanobis Distance p-value: 6.24e-005

Prediction: Positive if the Bayesian score is above the estimated best cutoff value from minimizing the false positive and false negative rate.
Probability: The esimated probability that the sample is in the positive category. This assumes that the Bayesian score follows a normal distribution and is different from the prediction using a cutoff.
Enrichment: An estimate of enrichment, that is, the increased likelihood (versus random) of this sample being in the category.
Bayesian Score: The standard Laplacian-modified Bayesian score.
Mahalanobis Distance: The Mahalanobis distance (MD) is the distance to the center of the training data. The larger the MD, the less trustworthy the prediction.
Mahalanobis Distance p-value: The p-value gives the fraction of training data with an MD greater than or equal to the one for the given sample, assuming normally distributed data. The smaller the p-value, the less trustworthy the prediciton. For highly non-normal X properties (e.g., fingerprints), the MD p-value is wildly inaccurate.

Structural Similar Compounds

Name	Bicalutamide	Glipizide	Penicillin
Structure	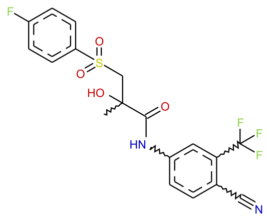	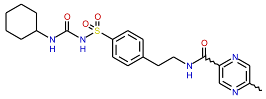	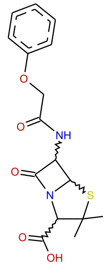
Actual Endpoint	Non-Carcinogen	Non-Carcinogen	Non-Carcinogen
Predicted Endpoint	Non-Carcinogen	Non-Carcinogen	Non-Carcinogen
Distance	0.610	0.646	0.670
Reference	US FDA (Centre for Drug Eval.& Res./Off. Testing & Res.) Sept. 1997	US FDA (Centre for Drug Eval.& Res./Off. Testing & Res.) Sept. 1997	US FDA (Centre for Drug Eval.& Res./Off. Testing & Res.) Sept. 1997

Model Applicability

Unknown features are fingerprint features in the query molecule, but not found or appearing too infrequently in the training set.

1. All properties and OPS components are within expected ranges.

Feature Contribution

Top features for positive contribution

Fingerprint	Bit/Smiles	Feature Structure	Score	Carcinogen in training set
ECFP_6	769925792	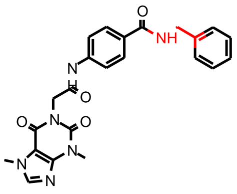 [*]NC[c](:[*]):[*]	0.617	2 out of 2

ECFP_6	738938915	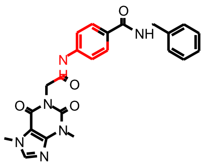<br>[*]C(=[*])N[c]1:[cH]:[cH]:[*]:[cH]:[cH]:1	0.617	2 out of 2
ECFP_6	-223149939	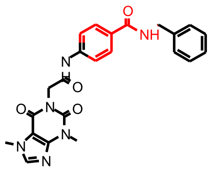<br>[*]NC(=O)[c]1:[cH]:[cH]:[*]:[cH]:[cH]:1	0.442	2 out of 3

Top Features for negative contribution

Fingerprint	Bit/Smiles	Feature Structure	Score	Carcinogen in training set
ECFP_6	-661097313	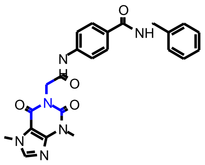<br>[*]CN(C(=[*])[*])C(=[*])[*]	-1.55	0 out of 12
ECFP_6	1731843802	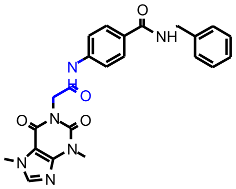<br>[*]CC(=O)N[*]	-0.657	0 out of 3
ECFP_6	-1677427181	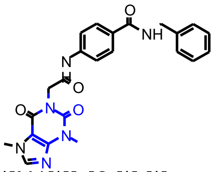<br>[*]N1[*][c]2:[*]:[*]:n:[c]:2N(C)C1=O	-0.482	0 out of 2

Sorafenib

TOPKAT_Mouse_Female_FDA_None_vs_Carcinogen

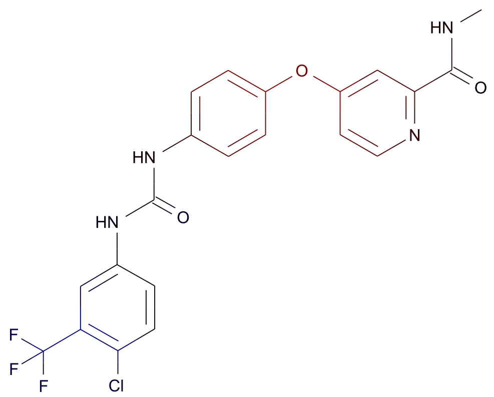

$C_{21}H_{16}ClF_3N_4O_3$
Molecular Weight: 464.82494
ALogP: 4.175
Rotatable Bonds: 6
Acceptors: 4
Donors: 3

Model Prediction

Prediction: Carcinogen
Probability: 0.257
Enrichment: 0.801
Bayesian Score: -0.321
Mahalanobis Distance: 14.9
Mahalanobis Distance p-value: 4.21e-007

Prediction: Positive if the Bayesian score is above the estimated best cutoff value from minimizing the false positive and false negative rate.
Probability: The esimated probability that the sample is in the positive category. This assumes that the Bayesian score follows a normal distribution and is different from the prediction using a cutoff.
Enrichment: An estimate of enrichment, that is, the increased likelihood (versus random) of this sample being in the category.
Bayesian Score: The standard Laplacian-modified Bayesian score.
Mahalanobis Distance: The Mahalanobis distance (MD) is the distance to the center of the training data. The larger the MD, the less trustworthy the prediction.
Mahalanobis Distance p-value: The p-value gives the fraction of training data with an MD greater than or equal to the one for the given sample, assuming normally distributed data. The smaller the p-value, the less trustworthy the prediciton. For highly non-normal X properties (e.g., fingerprints), the MD p-value is wildly inaccurate.

Structural Similar Compounds

Name	Glimepride	Glyburide	Fluvastatin
Structure	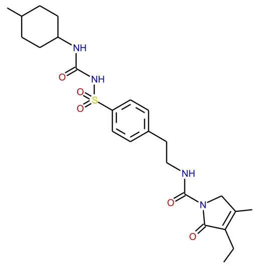	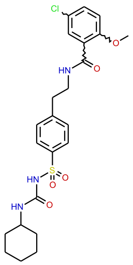	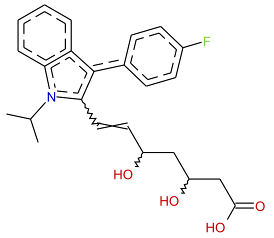
Actual Endpoint	Carcinogen	Non-Carcinogen	Non-Carcinogen
Predicted Endpoint	Carcinogen	Non-Carcinogen	Non-Carcinogen
Distance	0.605	0.615	0.625
Reference	US FDA (Centre for Drug Eval.& Res./Off. Testing & Res.) Sept. 1997	US FDA (Centre for Drug Eval.& Res./Off. Testing & Res.) Sept. 1997	US FDA (Centre for Drug Eval.& Res./Off. Testing & Res.) Sept. 1997

Model Applicability

Unknown features are fingerprint features in the query molecule, but not found or appearing too infrequently in the training set.

1. OPS PC20 out of range. Value: -3.3309. Training min, max, SD, explained variance: -3.1862, 4.4571, 1.28, 0.0167.

Feature Contribution

Top features for positive contribution				
**Fingerprint**	**Bit/Smiles**	**Feature Structure**	**Score**	**Carcinogen in training set**
ECFP_6	738938915	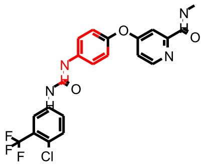 [*]C(=[*])N[c]1:[cH]:[cH]:[*]:[cH]:[cH]:1	0.617	2 out of 2

ECFP_6	1338334141	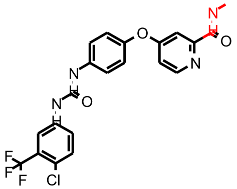 [*]C(=[*])NC	0.442	2 out of 3
ECFP_6	-335167981	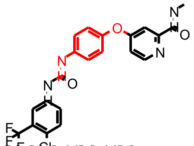 [*]O[c]1:[cH]:[cH]:[c](NC(=[*])[*]):[cH]:[cH]:1	0.424	1 out of 1
**Top Features for negative contribution**				
**Fingerprint**	**Bit/Smiles**	**Feature Structure**	**Score**	**Carcinogen in training set**
ECFP_6	1335691903	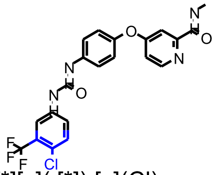 [*][c](:[*]):[c](Cl):[cH]:[*]	-0.669	3 out of 22
ECFP_6	1336678434	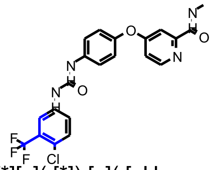 [*][c](:[*]):[c](:[cH]:[*])C([*])([*])[*]	-0.657	0 out of 3
ECFP_6	-1952889961	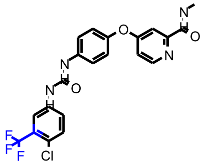 [*]:[c](:[*])C(F)(F)F	-0.657	0 out of 3

Sorafenib

TOPKAT_Mouse_Female_FDA_Single_vs_Multiple

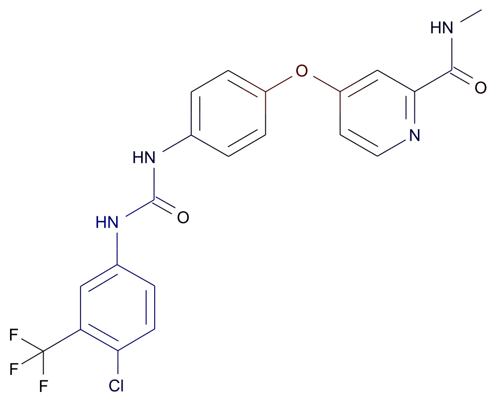

$C_{21}H_{16}ClF_3N_4O_3$
Molecular Weight: 464.82494
ALogP: 4.175
Rotatable Bonds: 6
Acceptors: 4
Donors: 3

Model Prediction

Prediction: Single-Carcinogen
Probability: 0.283
Enrichment: 0.691
Bayesian Score: -3.89
Mahalanobis Distance: 11.1
Mahalanobis Distance p-value: 0.00221

Prediction: Positive if the Bayesian score is above the estimated best cutoff value from minimizing the false positive and false negative rate.
Probability: The esimated probability that the sample is in the positive category. This assumes that the Bayesian score follows a normal distribution and is different from the prediction using a cutoff.
Enrichment: An estimate of enrichment, that is, the increased likelihood (versus random) of this sample being in the category.
Bayesian Score: The standard Laplacian-modified Bayesian score.
Mahalanobis Distance: The Mahalanobis distance (MD) is the distance to the center of the training data. The larger the MD, the less trustworthy the prediction.
Mahalanobis Distance p-value: The p-value gives the fraction of training data with an MD greater than or equal to the one for the given sample, assuming normally distributed data. The smaller the p-value, the less trustworthy the prediciton. For highly non-normal X properties (e.g., fingerprints), the MD p-value is wildly inaccurate.

Structural Similar Compounds

Name	Glimepride	Labetalol	Lansoprazole
Structure	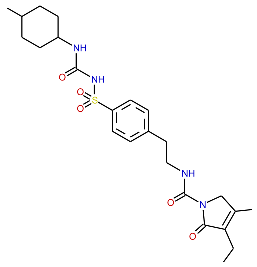	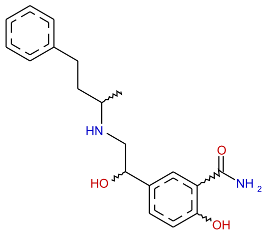	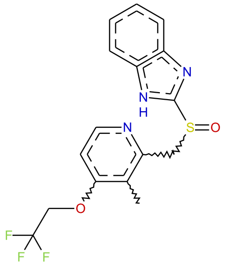
Actual Endpoint	Single-Carcinogen	Single-Carcinogen	Single-Carcinogen
Predicted Endpoint	Single-Carcinogen	Single-Carcinogen	Single-Carcinogen
Distance	0.599	0.808	0.820
Reference	US FDA (Centre for Drug Eval.& Res./Off. Testing & Res.) Sept. 1997	US FDA (Centre for Drug Eval.& Res./Off. Testing & Res.) Sept. 1997	US FDA (Centre for Drug Eval.& Res./Off. Testing & Res.) Sept. 1997

Model Applicability

Unknown features are fingerprint features in the query molecule, but not found or appearing too infrequently in the training set.

1. All properties and OPS components are within expected ranges.
2. Unknown ECFP_2 feature: 1336678434: [*][c](:[*]):[c](C([*])([*])[*]):c:[*]
3. Unknown ECFP_2 feature: -1952889961: [*]:[c](:[*])C(F)(F)F

Feature Contribution

Top features for positive contribution

Fingerprint	Bit/Smiles	Feature Structure	Score	Multiple-Carcinogen in training set
ECFP_4	-834094296	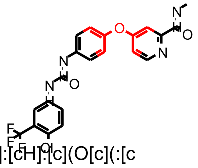 [*]:[cH]:[c](O[c](:[cH]:[*]):[cH]:[*]):[cH]:[*]	0.351	1 out of 1

ECFP_4	1407472008	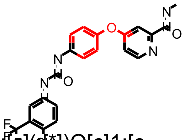<br>[*]:[c](:[*])O[c]1:[c<br>H]:[cH]:[*]:[cH]:[cH<br>]:1	0.351	1 out of 1
ECFP_4	143734695	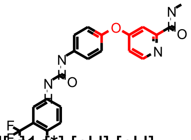<br>[*][c]1:[*]:[cH]:[cH]<br>:[c](O[c](:[*]):[*])<br>:[cH]:1	0.351	1 out of 1

Top Features for negative contribution

Fingerprint	Bit/Smiles	Feature Structure	Score	Multiple-Carcinogen in training set
ECFP_4	888054369	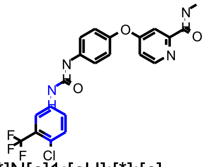<br>[*]N[c]1:[cH]:[*]:[c]<br>([*]):[cH]:[cH]:1	-0.8	0 out of 3
ECFP_4	1335691903	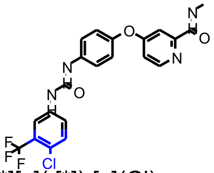<br>[*][c](:[*]):[c](Cl):<br>[cH]:[*]	-0.8	0 out of 3
ECFP_4	1338334141	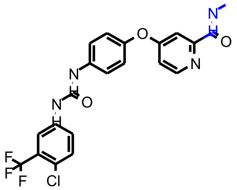<br>[*]C(=[*])NC	-0.597	0 out of 2

11RR

TOPKAT_Mouse_Male_FDA_None_vs_Carcinogen

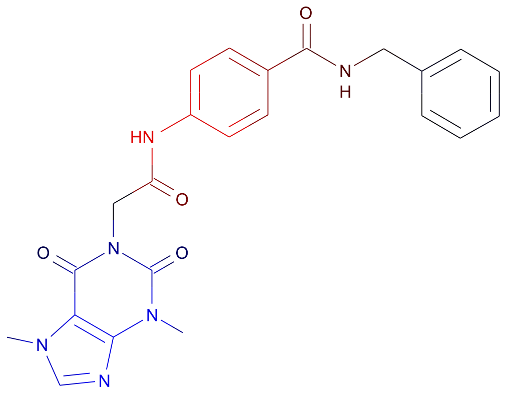

$C_{23}H_{22}N_6O_4$

Molecular Weight: 446.45858

ALogP: 1.328

Rotatable Bonds: 6

Acceptors: 5

Donors: 2

Model Prediction

Prediction: Carcinogen

Probability: 0.268

Enrichment: 0.909

Bayesian Score: -1.51

Mahalanobis Distance: 13.9

Mahalanobis Distance p-value: 1.07e-005

Prediction: Positive if the Bayesian score is above the estimated best cutoff value from minimizing the false positive and false negative rate.
Probability: The esimated probability that the sample is in the positive category. This assumes that the Bayesian score follows a normal distribution and is different from the prediction using a cutoff.
Enrichment: An estimate of enrichment, that is, the increased likelihood (versus random) of this sample being in the category.
Bayesian Score: The standard Laplacian-modified Bayesian score.
Mahalanobis Distance: The Mahalanobis distance (MD) is the distance to the center of the training data. The larger the MD, the less trustworthy the prediction.
Mahalanobis Distance p-value: The p-value gives the fraction of training data with an MD greater than or equal to the one for the given sample, assuming normally distributed data. The smaller the p-value, the less trustworthy the prediciton. For highly non-normal X properties (e.g., fingerprints), the MD p-value is wildly inaccurate.

Structural Similar Compounds

Name	Bicalutamide	Glipizide	Penicillin
Structure	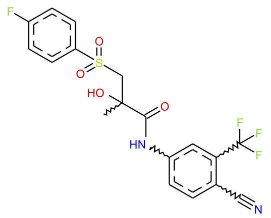	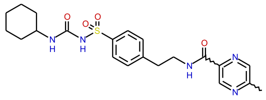	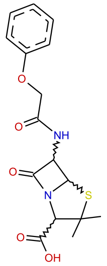
Actual Endpoint	Carcinogen	Non-Carcinogen	Non-Carcinogen
Predicted Endpoint	Carcinogen	Non-Carcinogen	Non-Carcinogen
Distance	0.586	0.641	0.679
Reference	US FDA (Centre for Drug Eval.& Res./Off. Testing & Res.) Sept. 1997	US FDA (Centre for Drug Eval.& Res./Off. Testing & Res.) Sept. 1997	US FDA (Centre for Drug Eval.& Res./Off. Testing & Res.) Sept. 1997

Model Applicability

Unknown features are fingerprint features in the query molecule, but not found or appearing too infrequently in the training set.

1. All properties and OPS components are within expected ranges.

Feature Contribution

Top features for positive contribution

Fingerprint	Bit/Smiles	Feature Structure	Score	Carcinogen in training set
FCFP_6	-581879738	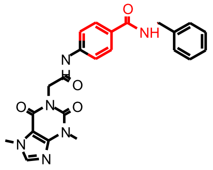 [*]NC(=O)[c]1:[cH]:[cH]:[*]:[cH]:[cH]:1	0.77	4 out of 5

FCFP_6	382195549	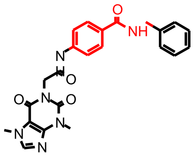 [*]CNC(=O)[c]1:[cH]:[cH]:[c]([*]):[cH]:[cH]:1	0.676	2 out of 2
FCFP_6	-451043714	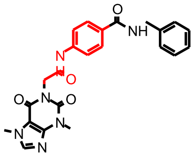 [*]CC(=O)N[c]1:[cH]:[cH]:[c]([*]):[cH]:[cH]:1	0.676	2 out of 2

Top Features for negative contribution

Fingerprint	Bit/Smiles	Feature Structure	Score	Carcinogen in training set
FCFP_6	-124685461	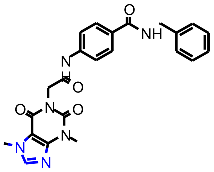 [*]n1:[*]:[*]:n:[cH]:1	-0.731	1 out of 12
FCFP_6	-1553874037	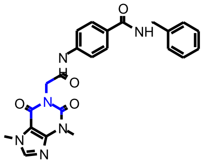 [*]CN(C(=[*])[*])C(=[*])[*]	-0.45	5 out of 32
FCFP_6	889850438	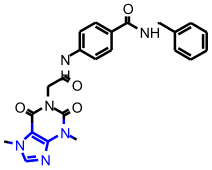 [*]N([*])[c]1:n:[cH]:n([*]):[c]:1[*]	-0.423	0 out of 2

Sorafenib

TOPKAT_Mouse_Male_FDA_None_vs_Carcinogen

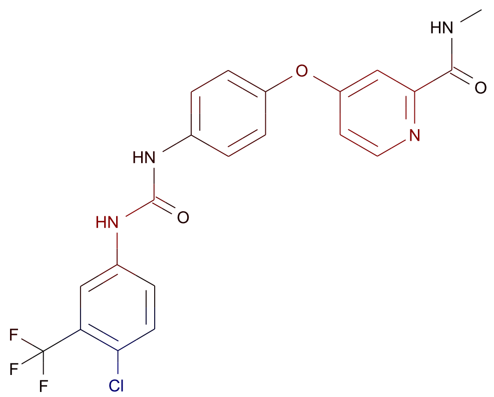

$C_{21}H_{16}ClF_3N_4O_3$
Molecular Weight: 464.82494
ALogP: 4.175
Rotatable Bonds: 6
Acceptors: 4
Donors: 3

Structural Similar Compounds

Name	Glyburide	Glimepride	Fluvastatin
Structure	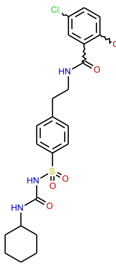	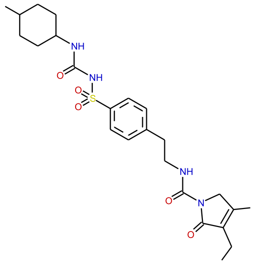	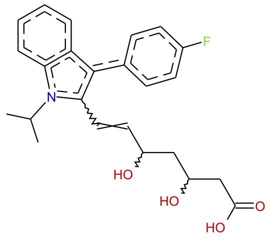
Actual Endpoint	Non-Carcinogen	Carcinogen	Non-Carcinogen
Predicted Endpoint	Non-Carcinogen	Carcinogen	Non-Carcinogen
Distance	0.594	0.599	0.603
Reference	US FDA (Centre for Drug Eval.& Res./Off. Testing & Res.) Sept. 1997	US FDA (Centre for Drug Eval.& Res./Off. Testing & Res.) Sept. 1997	US FDA (Centre for Drug Eval.& Res./Off. Testing & Res.) Sept. 1997

Model Prediction

Prediction: Carcinogen
Probability: 0.444
Enrichment: 1.51
Bayesian Score: 4.21
Mahalanobis Distance: 20.3
Mahalanobis Distance p-value: 1.28e-019

Prediction: Positive if the Bayesian score is above the estimated best cutoff value from minimizing the false positive and false negative rate.
Probability: The esimated probability that the sample is in the positive category. This assumes that the Bayesian score follows a normal distribution and is different from the prediction using a cutoff.
Enrichment: An estimate of enrichment, that is, the increased likelihood (versus random) of this sample being in the category.
Bayesian Score: The standard Laplacian-modified Bayesian score.
Mahalanobis Distance: The Mahalanobis distance (MD) is the distance to the center of the training data. The larger the MD, the less trustworthy the prediction.
Mahalanobis Distance p-value: The p-value gives the fraction of training data with an MD greater than or equal to the one for the given sample, assuming normally distributed data. The smaller the p-value, the less trustworthy the prediciton. For highly non-normal X properties (e.g., fingerprints), the MD p-value is wildly inaccurate.

Model Applicability

Unknown features are fingerprint features in the query molecule, but not found or appearing too infrequently in the training set.

1. All properties and OPS components are within expected ranges.

Feature Contribution

Top features for positive contribution

Fingerprint	Bit/Smiles	Feature Structure	Score	Carcinogen in training set
FCFP_6	71953198	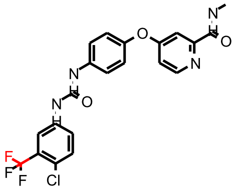 [*]C([*])([*])F	0.612	12 out of 23

FCFP_6	-1838187238	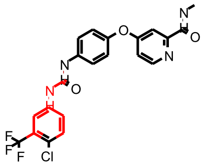<br>[*]C(=[*])N[c]1:[cH]:<br>[cH]:[*]:[cH]:[cH]:1	0.565	4 out of 7
FCFP_6	140656626	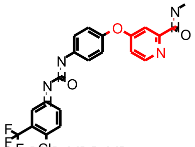<br>[*]O[c]1:[cH]:[cH]:n:<br>[c](:[cH]:1)C(=[*])[<br>*]	0.46	1 out of 1

Top Features for negative contribution

Fingerprint	Bit/Smiles	Feature Structure	Score	Carcinogen in training set
FCFP_6	2104062943	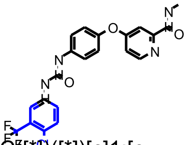<br>[*]C([*])([*])[c]1:[c<br>H]:[*]:[cH]:[cH]:[c]<br>:1Cl	-1.01	1 out of 17
FCFP_6	551850122	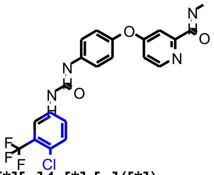<br>[*][c]1:[*]:[c]([*]):<br>[c](Cl):[cH]:[cH]:1	-0.433	8 out of 49
FCFP_6	71476542	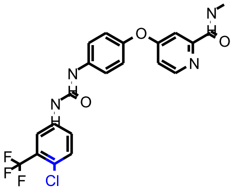<br>[*]:[c](:[*])Cl	-0.406	10 out of 59

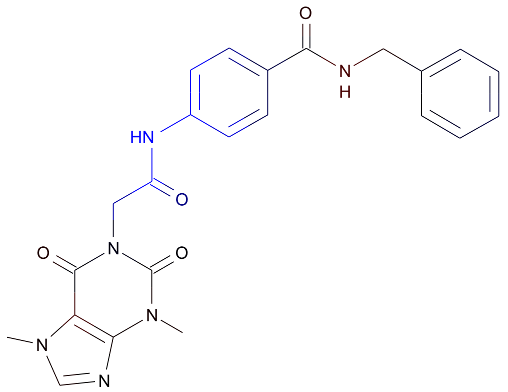

$C_{23}H_{22}N_6O_4$

Molecular Weight: 446.45858

ALogP: 1.328

Rotatable Bonds: 6

Acceptors: 5

Donors: 2

Model Prediction

Prediction: Single-Carcinogen

Probability: 0.147

Enrichment: 0.487

Bayesian Score: -10.5

Mahalanobis Distance: 14.2

Mahalanobis Distance p-value: 1e-005

Prediction: Positive if the Bayesian score is above the estimated best cutoff value from minimizing the false positive and false negative rate.
Probability: The esimated probability that the sample is in the positive category. This assumes that the Bayesian score follows a normal distribution and is different from the prediction using a cutoff.
Enrichment: An estimate of enrichment, that is, the increased likelihood (versus random) of this sample being in the category.
Bayesian Score: The standard Laplacian-modified Bayesian score.
Mahalanobis Distance: The Mahalanobis distance (MD) is the distance to the center of the training data. The larger the MD, the less trustworthy the prediction.
Mahalanobis Distance p-value: The p-value gives the fraction of training data with an MD greater than or equal to the one for the given sample, assuming normally distributed data. The smaller the p-value, the less trustworthy the prediciton. For highly non-normal X properties (e.g., fingerprints), the MD p-value is wildly inaccurate.

Structural Similar Compounds

Name	Bicalutamide	Primidolol	Cetirizine
Structure	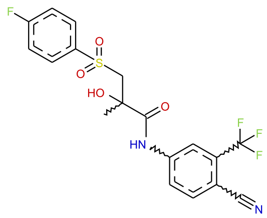	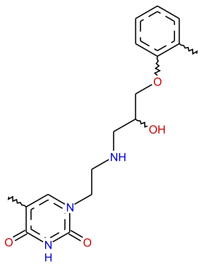	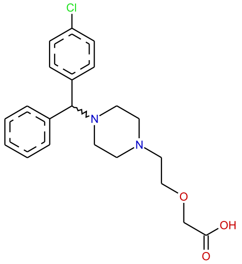
Actual Endpoint	Single-Carcinogen	Single-Carcinogen	Single-Carcinogen
Predicted Endpoint	Single-Carcinogen	Single-Carcinogen	Single-Carcinogen
Distance	0.599	0.769	0.770
Reference	US FDA (Centre for Drug Eval.& Res./Off. Testing & Res.) Sept. 1997	US FDA (Centre for Drug Eval.& Res./Off. Testing & Res.) Sept. 1997	US FDA (Centre for Drug Eval.& Res./Off. Testing & Res.) Sept. 1997

Model Applicability

Unknown features are fingerprint features in the query molecule, but not found or appearing too infrequently in the training set.

1. OPS PC2 out of range. Value: 5.0112. Training min, max, SD, explained variance: -5.2888, 4.2744, 2.566, 0.1229.

Feature Contribution

Top features for positive contribution

Fingerprint	Bit/Smiles	Feature Structure	Score	Multiple-Carcinogen in training set
FCFP_12	-1549163031	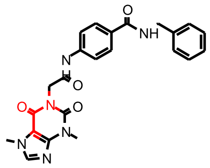 [*]N([*])C(=O)[c](:[*]):[*]	0.683	3 out of 3

FCFP_12	675769755	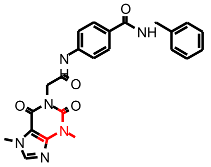 [*]C(=[*])N(C)[c](:[*]):[*]	0.573	5 out of 7
FCFP_12	907096426	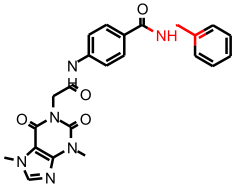 [*]NC[c](:[*]):[*]	0.4	1 out of 1

Top Features for negative contribution

Fingerprint	Bit/Smiles	Feature Structure	Score	Multiple-Carcinogen in training set
FCFP_12	1294255210	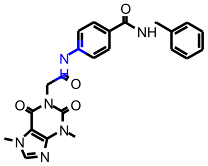 [*]C(=[*])N[c](:[*]): [*]	-1.63	0 out of 12
FCFP_12	1175665944	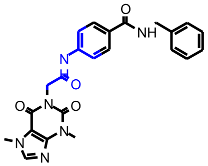 [*]CC(=O)N[c](:[cH]:[*]):[cH]:[*]	-1.22	0 out of 7
FCFP_12	590925877	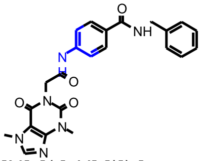 [*]N[c](:[cH]:[*]):[c H]:[*]	-0.998	1 out of 13

Sorafenib

TOPKAT_Mouse_Male_FDA_Single_vs_Multiple

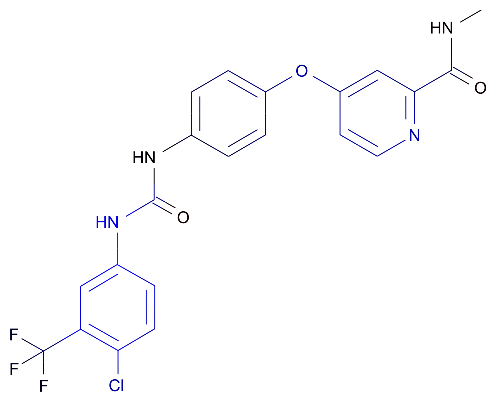

$C_{21}H_{16}ClF_3N_4O_3$
Molecular Weight: 464.82494
ALogP: 4.175
Rotatable Bonds: 6
Acceptors: 4
Donors: 3

Model Prediction

Prediction: Single-Carcinogen
Probability: 0.139
Enrichment: 0.461
Bayesian Score: -14.7
Mahalanobis Distance: 21.3
Mahalanobis Distance p-value: 4.93e-011

Prediction: Positive if the Bayesian score is above the estimated best cutoff value from minimizing the false positive and false negative rate.
Probability: The esimated probability that the sample is in the positive category. This assumes that the Bayesian score follows a normal distribution and is different from the prediction using a cutoff.
Enrichment: An estimate of enrichment, that is, the increased likelihood (versus random) of this sample being in the category.
Bayesian Score: The standard Laplacian-modified Bayesian score.
Mahalanobis Distance: The Mahalanobis distance (MD) is the distance to the center of the training data. The larger the MD, the less trustworthy the prediction.
Mahalanobis Distance p-value: The p-value gives the fraction of training data with an MD greater than or equal to the one for the given sample, assuming normally distributed data. The smaller the p-value, the less trustworthy the prediciton. For highly non-normal X properties (e.g., fingerprints), the MD p-value is wildly inaccurate.

Structural Similar Compounds

Name	Glimepride	Bicalutamide	Lansoprazole
Structure	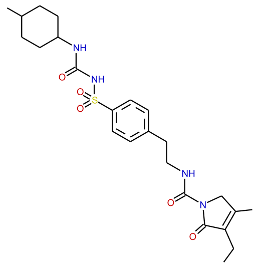	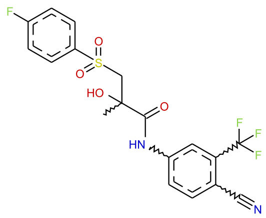	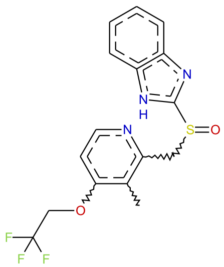
Actual Endpoint	Single-Carcinogen	Single-Carcinogen	Single-Carcinogen
Predicted Endpoint	Single-Carcinogen	Single-Carcinogen	Single-Carcinogen
Distance	0.626	0.700	0.866
Reference	US FDA (Centre for Drug Eval.& Res./Off. Testing & Res.) Sept. 1997	US FDA (Centre for Drug Eval.& Res./Off. Testing & Res.) Sept. 1997	US FDA (Centre for Drug Eval.& Res./Off. Testing & Res.) Sept. 1997

Model Applicability

Unknown features are fingerprint features in the query molecule, but not found or appearing too infrequently in the training set.

1. All properties and OPS components are within expected ranges.

Feature Contribution

Top features for positive contribution

Fingerprint	Bit/Smiles	Feature Structure	Score	Multiple-Carcinogen in training set
FCFP_12	1499521844	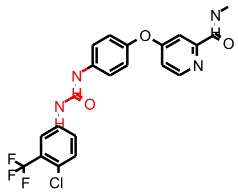 [*]NC(=O)N[*]	0.39	5 out of 9

FCFP_12	-904785030	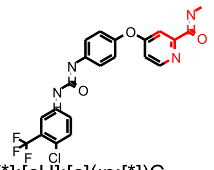 [*]:[cH]:[c](:n:[*])C(=O)NC	0.174	1 out of 2
FCFP_12	-1549103449	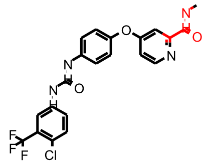 [*]NC(=O)[c](:[*]):[*]	0.168	3 out of 7

Top Features for negative contribution

**Fingerprint**	**Bit/Smiles**	**Feature Structure**	**Score**	**Multiple-Carcinogen in training set**
FCFP_12	1294255210	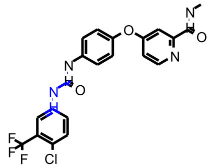 [*]C(=[*])N[c](:[*]):[*]	-1.63	0 out of 12
FCFP_12	590925877	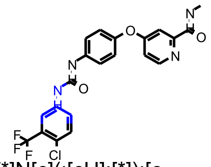 [*]N[c](:[cH]:[*]):[cH]:[*]	-0.998	1 out of 13
FCFP_12	-1462709112	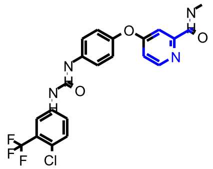 [*]C(=[*])[c]1:[cH]:[*]:[cH]:[cH]:n:1	-0.994	0 out of 5

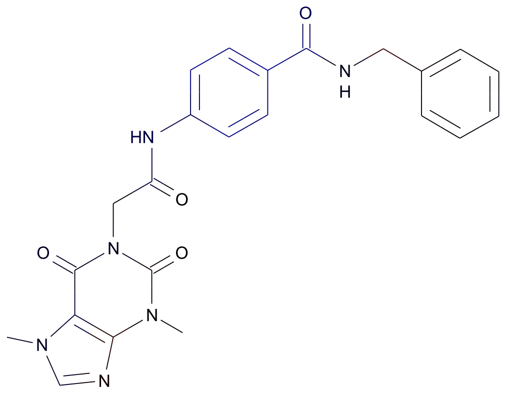

$C_{23}H_{22}N_6O_4$
Molecular Weight: 446.45858
ALogP: 1.328
Rotatable Bonds: 6
Acceptors: 5
Donors: 2

Model Prediction

Prediction: Mild

Probability: 0.678

Enrichment: 0.984

Bayesian Score: -3.89

Mahalanobis Distance: 11.1

Mahalanobis Distance p-value: 0.00347

Prediction: Positive if the Bayesian score is above the estimated best cutoff value from minimizing the false positive and false negative rate.
Probability: The esimated probability that the sample is in the positive category. This assumes that the Bayesian score follows a normal distribution and is different from the prediction using a cutoff.
Enrichment: An estimate of enrichment, that is, the increased likelihood (versus random) of this sample being in the category.
Bayesian Score: The standard Laplacian-modified Bayesian score.
Mahalanobis Distance: The Mahalanobis distance (MD) is the distance to the center of the training data. The larger the MD, the less trustworthy the prediction.
Mahalanobis Distance p-value: The p-value gives the fraction of training data with an MD greater than or equal to the one for the given sample, assuming normally distributed data. The smaller the p-value, the less trustworthy the prediciton. For highly non-normal X properties (e.g., fingerprints), the MD p-value is wildly inaccurate.

Structural Similar Compounds

Name	5-NORBORNENE-2;3-DICARBOXYLIC ACID; 1;4;5;6;7;7-HEXACHLORO-	1-AMINO-4-BENZOYLAMINO-ANTHRAQUINONE	ANTHRAQUINONE; 1-AMINO-4-HYDROXY-2-PHENOXY-
Structure	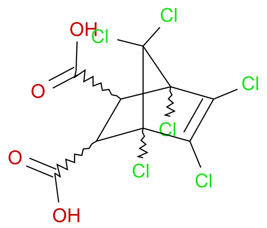	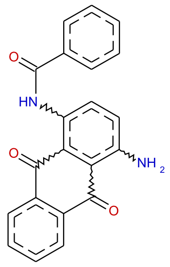	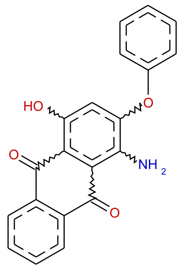
Actual Endpoint	Moderate_Severe	Mild	Mild
Predicted Endpoint	Moderate_Severe	Mild	Mild
Distance	0.701	0.723	0.775
Reference	28ZPAK-;92;72	28ZPAK-;124;72	28ZPAK 239;72

Model Applicability

Unknown features are fingerprint features in the query molecule, but not found or appearing too infrequently in the training set.

1. All properties and OPS components are within expected ranges.
2. Unknown FCFP_2 feature: -124685461: [*]n1:[*]:[*]:n:[cH]:1
3. Unknown FCFP_2 feature: 136150461: [*]:n(:[*])C

Feature Contribution

Top features for positive contribution				
**Fingerprint**	**Bit/Smiles**	**Feature Structure**	**Score**	**Moderate_Severe in training set**
FCFP_10	907096426	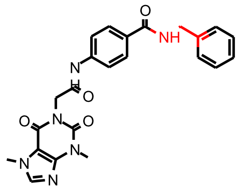 [*]NC[c](:[*]):[*]	0.332	5 out of 5

FCFP_10	427906732	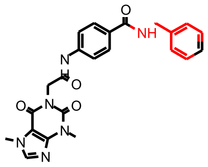 [*]NC[c]1:[cH]:[cH]:[*]:[cH]:[cH]:1	0.294	3 out of 3
FCFP_10	-1410049896	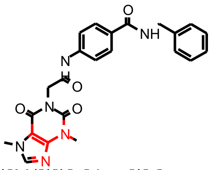 [*]N([*])[c]1:n:[*]:[*]:[c]:1[*]	0.256	2 out of 2

Top Features for negative contribution

Fingerprint	Bit/Smiles	Feature Structure	Score	Moderate_Severe in training set
FCFP_10	-581879738	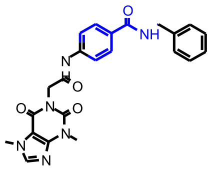 [*]NC(=O)[c]1:[cH]:[c H]:[*]:[cH]:[cH]:1	-1.29	0 out of 4
FCFP_10	-306856457	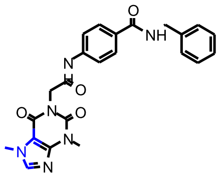 [*][c]1:[*]:[*]:[cH]: n:1C	-0.842	0 out of 2
FCFP_10	-1549163031	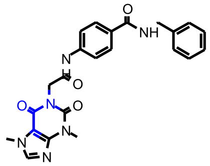 [*]N([*])C(=O)[c](:[*]):[*]	-0.657	5 out of 16

Sorafenib

TOPKAT_Ocular_Irritancy_Mild_vs_Moderate_Severe

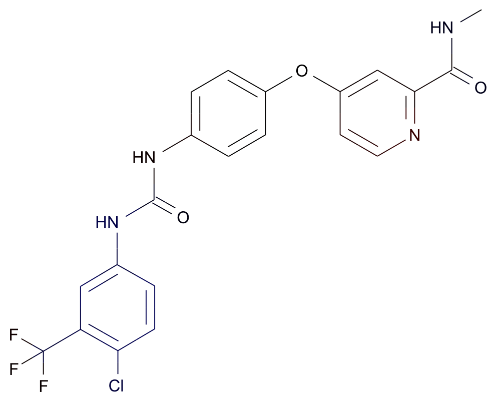

$C_{21}H_{16}ClF_3N_4O_3$
Molecular Weight: 464.82494
ALogP: 4.175
Rotatable Bonds: 6
Acceptors: 4
Donors: 3

Model Prediction

Prediction: Mild
Probability: 0.776
Enrichment: 1.13
Bayesian Score: -1.8
Mahalanobis Distance: 8.95
Mahalanobis Distance p-value: 0.537

Prediction: Positive if the Bayesian score is above the estimated best cutoff value from minimizing the false positive and false negative rate.
Probability: The esimated probability that the sample is in the positive category. This assumes that the Bayesian score follows a normal distribution and is different from the prediction using a cutoff.
Enrichment: An estimate of enrichment, that is, the increased likelihood (versus random) of this sample being in the category.
Bayesian Score: The standard Laplacian-modified Bayesian score.
Mahalanobis Distance: The Mahalanobis distance (MD) is the distance to the center of the training data. The larger the MD, the less trustworthy the prediction.
Mahalanobis Distance p-value: The p-value gives the fraction of training data with an MD greater than or equal to the one for the given sample, assuming normally distributed data. The smaller the p-value, the less trustworthy the prediciton. For highly non-normal X properties (e.g., fingerprints), the MD p-value is wildly inaccurate.

Structural Similar Compounds

Name	4;4'-DIAMINO-1;1'-DIANTHRIMIDE	5-NORBORNENE-2;3-DICARBOXYLIC ACID; 1;4;5;6;7;7-HEXACHLORO-	METHANE;TRIS(4-AMINOPHENYL)-
Structure	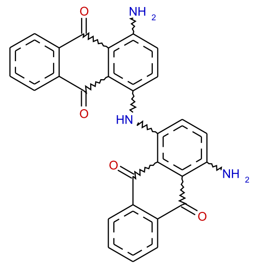	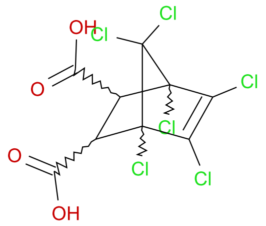	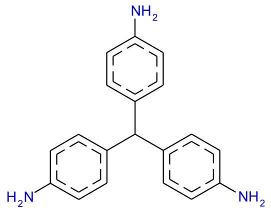
Actual Endpoint	Mild	Moderate_Severe	Moderate_Severe
Predicted Endpoint	Mild	Moderate_Severe	Moderate_Severe
Distance	0.799	0.816	0.827
Reference	28ZPAK-;125;72	28ZPAK-;92;72	28ZPAK-;73;72

Model Applicability

Unknown features are fingerprint features in the query molecule, but not found or appearing too infrequently in the training set.

1. All properties and OPS components are within expected ranges.

Feature Contribution

Top features for positive contribution

Fingerprint	Bit/Smiles	Feature Structure	Score	Moderate_Severe in training set
FCFP_10	-1695756380	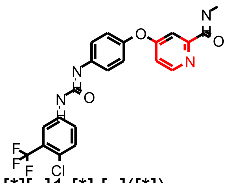 [*][c]1:[*]:[c]([*]):n:[cH]:[cH]:1	0.285	10 out of 11

FCFP_10	-124655670	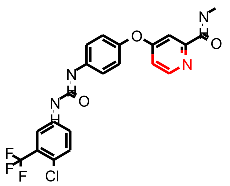 [*]:[cH]:[cH]:n:[*]	0.259	14 out of 16
FCFP_10	-885550502	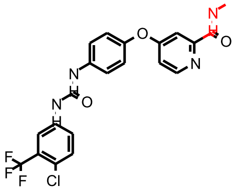 [*]CNC(=[*])[*]	0.239	54 out of 64

Top Features for negative contribution

**Fingerprint**	**Bit/Smiles**	**Feature Structure**	**Score**	**Moderate_Severe in training set**
FCFP_10	2104062943	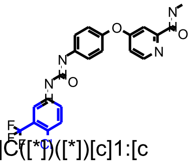 [*]C([*])([*])[c]1:[c<br>H]:[*]:[cH]:[cH]:[c]<br>:1Cl	-0.745	7 out of 24
FCFP_10	-174293376	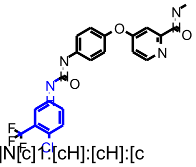 [*]N[c]1:[cH]:[cH]:[c<br>](Cl):[c](:[cH]:1)C(<br>[*])([*])[*]	-0.507	0 out of 1
FCFP_10	-1549103449	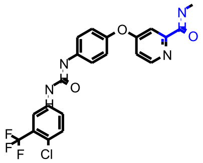 [*]NC(=O)[c](:[*]):[*<br>]	-0.504	2 out of 6

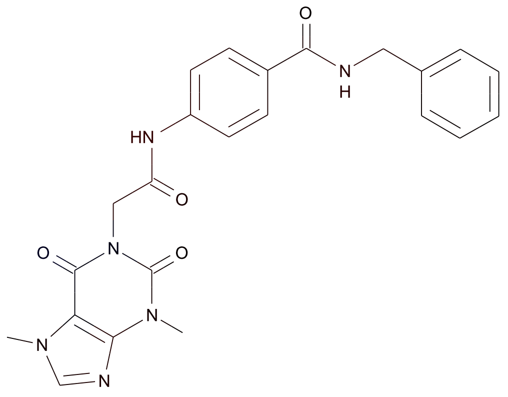

$C_{23}H_{22}N_6O_4$
Molecular Weight: 446.45858
ALogP: 1.328
Rotatable Bonds: 6
Acceptors: 5
Donors: 2

Structural Similar Compounds

Name	5-NORBORNENE-2;3-DICARBOXYLIC ACID; 1;4;5;6;7;7-HEXACHLORO-	1-AMINO-4-BENZOYLAMINO-ANTHRAQUINONE	ANTHRAQUINONE; 1-AMINO-4-HYDROXY-2-PHENOXY-
Structure	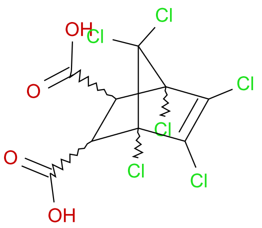	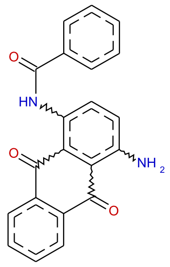	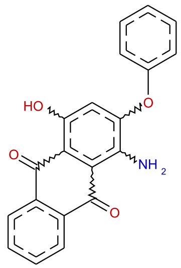
Actual Endpoint	Irritant	Irritant	Irritant
Predicted Endpoint	Irritant	Irritant	Irritant
Distance	0.684	0.706	0.750
Reference	28ZPAK-;92;72	28ZPAK-;124;72	28ZPAK 239;72

Model Prediction

Prediction: Irritant
Probability: 1
Enrichment: 1.18
Bayesian Score: 2.14
Mahalanobis Distance: 8.84
Mahalanobis Distance p-value: 0.601

Prediction: Positive if the Bayesian score is above the estimated best cutoff value from minimizing the false positive and false negative rate.
Probability: The esimated probability that the sample is in the positive category. This assumes that the Bayesian score follows a normal distribution and is different from the prediction using a cutoff.
Enrichment: An estimate of enrichment, that is, the increased likelihood (versus random) of this sample being in the category.
Bayesian Score: The standard Laplacian-modified Bayesian score.
Mahalanobis Distance: The Mahalanobis distance (MD) is the distance to the center of the training data. The larger the MD, the less trustworthy the prediction.
Mahalanobis Distance p-value: The p-value gives the fraction of training data with an MD greater than or equal to the one for the given sample, assuming normally distributed data. The smaller the p-value, the less trustworthy the prediciton. For highly non-normal X properties (e.g., fingerprints), the MD p-value is wildly inaccurate.

Model Applicability

Unknown features are fingerprint features in the query molecule, but not found or appearing too infrequently in the training set.

1. All properties and OPS components are within expected ranges.
2. Unknown FCFP_2 feature: -124685461: [*]n1:[*]:[*]:n:[cH]:1
3. Unknown FCFP_2 feature: 136150461: [*]:n(:[*])C

Feature Contribution

Top features for positive contribution

Fingerprint	Bit/Smiles	Feature Structure	Score	Irritant in training set
FCFP_12	1747237384	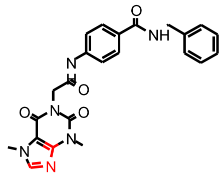 [*][c]1:[*]:[*]:[cH]:n:1	0.208	44 out of 44

FCFP_12	1175665944	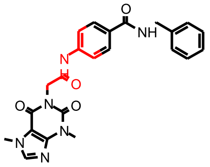<br>[*]CC(=O)N[c](:[cH]:[<br>*]):[cH]:[*]	0.198	14 out of 14
FCFP_12	-1539132615	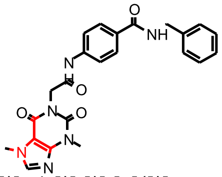<br>[*]n1:[*]:[*]:[c]([*]<br>):[c]:1C(=[*])[*]	0.197	13 out of 13
**Top Features for negative contribution**				
**Fingerprint**	**Bit/Smiles**	**Feature Structure**	**Score**	**Irritant in training set**
FCFP_12	-1549163031	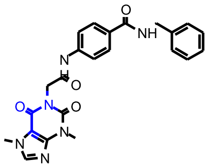<br>[*]N([*])C(=O)[c](:[*<br>]):[*]	-0.623	16 out of 38
FCFP_12	-1698724694	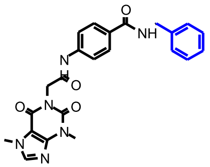<br>[*]C[c]1:[cH]:[cH]:[c<br>H]:[cH]:[cH]:1	-0.0964	107 out of 146
FCFP_12	1	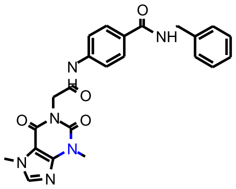<br>[*]N([*])[*]	0	872 out of 1051

Sorafenib

TOPKAT_Ocular_Irritancy_None_vs_Irritant

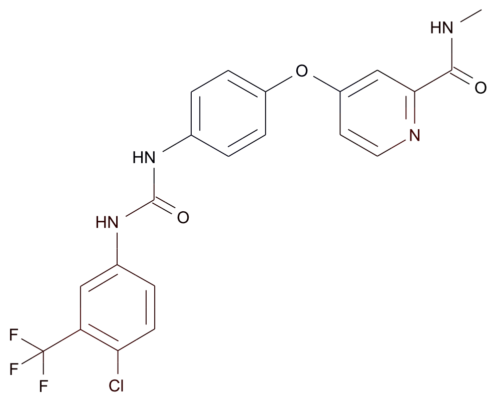

$C_{21}H_{16}ClF_3N_4O_3$

Molecular Weight: 464.82494

ALogP: 4.175

Rotatable Bonds: 6

Acceptors: 4

Donors: 3

Model Prediction

Prediction: Irritant

Probability: 1

Enrichment: 1.18

Bayesian Score: 3.04

Mahalanobis Distance: 6.28

Mahalanobis Distance p-value: 1

Prediction: Positive if the Bayesian score is above the estimated best cutoff value from minimizing the false positive and false negative rate.
Probability: The esimated probability that the sample is in the positive category. This assumes that the Bayesian score follows a normal distribution and is different from the prediction using a cutoff.
Enrichment: An estimate of enrichment, that is, the increased likelihood (versus random) of this sample being in the category.
Bayesian Score: The standard Laplacian-modified Bayesian score.
Mahalanobis Distance: The Mahalanobis distance (MD) is the distance to the center of the training data. The larger the MD, the less trustworthy the prediction.
Mahalanobis Distance p-value: The p-value gives the fraction of training data with an MD greater than or equal to the one for the given sample, assuming normally distributed data. The smaller the p-value, the less trustworthy the prediciton. For highly non-normal X properties (e.g., fingerprints), the MD p-value is wildly inaccurate.

Structural Similar Compounds

Name	BENZANILIDE;2';2'''-DITHIOBIS-	4;4'-DIAMINO-1;1'-DIANTHRIMIDE	5-NORBORNENE-2;3-DICARBOXYLIC ACID; 1;4;5;6;7;7-HEXACHLORO-
Structure	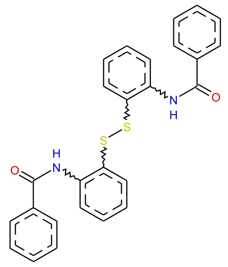	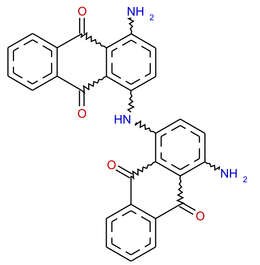	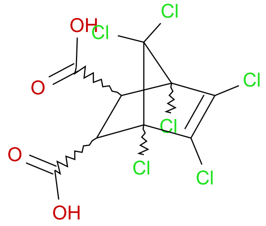
Actual Endpoint	Non-Irritant	Irritant	Irritant
Predicted Endpoint	Non-Irritant	Irritant	Irritant
Distance	0.743	0.791	0.801
Reference	28ZPAK-;173;72	28ZPAK-;125;72	28ZPAK-;92;72

Model Applicability

Unknown features are fingerprint features in the query molecule, but not found or appearing too infrequently in the training set.

1. All properties and OPS components are within expected ranges.

Feature Contribution

Top features for positive contribution				
**Fingerprint**	**Bit/Smiles**	**Feature Structure**	**Score**	**Irritant in training set**
FCFP_12	1747237384	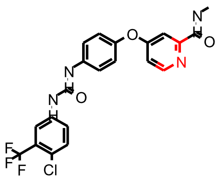 [*][c]1:[*]:[*]:[cH]:n:1	0.208	44 out of 44

FCFP_12	-124655670	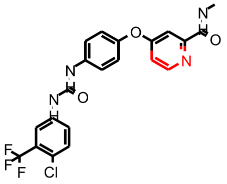 [*]:[cH]:[cH]:n:[*]	0.2	16 out of 16
FCFP_12	-1539132615	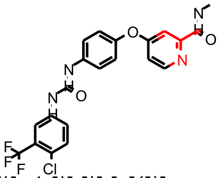 [*]n1:[*]:[*]:[c]([*]):[c]:1C(=[*])[*]	0.197	13 out of 13

Top Features for negative contribution

Fingerprint	Bit/Smiles	Feature Structure	Score	Irritant in training set
FCFP_12	-747629521	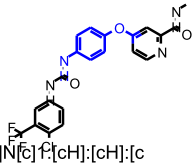 [*]N[c]1:[cH]:[cH]:[c](O[c](:[*]):[*]):[cH]:[cH]:1	-0.268	1 out of 2
FCFP_12	702861189	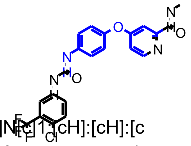 [*]N[c]1:[cH]:[cH]:[c](O[c]2:[cH]:[cH]:[*]:[c]([*]):[cH]:2):[cH]:[cH]:1	-0.268	1 out of 2
FCFP_12	859018953	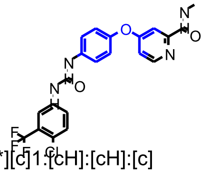 [*][c]1:[cH]:[cH]:[c](O[c](:[cH]:[*]):[cH]:[*]):[cH]:[cH]:1	0	7 out of 9

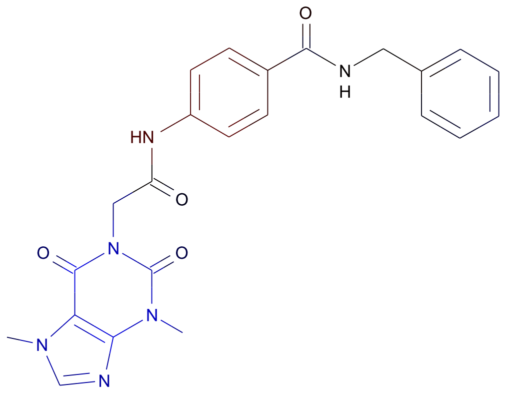

$C_{23}H_{22}N_6O_4$
Molecular Weight: 446.45858
ALogP: 1.328
Rotatable Bonds: 6
Acceptors: 5
Donors: 2

Model Prediction

Prediction: Non-Carcinogen
Probability: 0.218
Enrichment: 0.677
Bayesian Score: -5.29
Mahalanobis Distance: 13.5
Mahalanobis Distance p-value: 2.25e-005

Prediction: Positive if the Bayesian score is above the estimated best cutoff value from minimizing the false positive and false negative rate.
Probability: The esimated probability that the sample is in the positive category. This assumes that the Bayesian score follows a normal distribution and is different from the prediction using a cutoff.
Enrichment: An estimate of enrichment, that is, the increased likelihood (versus random) of this sample being in the category.
Bayesian Score: The standard Laplacian-modified Bayesian score.
Mahalanobis Distance: The Mahalanobis distance (MD) is the distance to the center of the training data. The larger the MD, the less trustworthy the prediction.
Mahalanobis Distance p-value: The p-value gives the fraction of training data with an MD greater than or equal to the one for the given sample, assuming normally distributed data. The smaller the p-value, the less trustworthy the prediciton. For highly non-normal X properties (e.g., fingerprints), the MD p-value is wildly inaccurate.

Structural Similar Compounds

Name	Bicalutamide	Glipizide	Penicillin
Structure	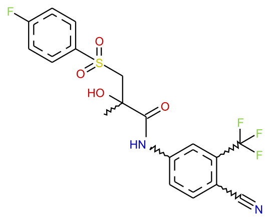	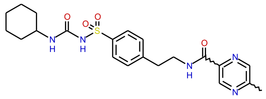	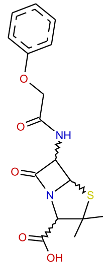
Actual Endpoint	Carcinogen	Non-Carcinogen	Non-Carcinogen
Predicted Endpoint	Carcinogen	Non-Carcinogen	Non-Carcinogen
Distance	0.628	0.667	0.694
Reference	US FDA (Centre for Drug Eval.& Res./Off. Testing & Res.) Sept. 1997	US FDA (Centre for Drug Eval.& Res./Off. Testing & Res.) Sept. 1997	US FDA (Centre for Drug Eval.& Res./Off. Testing & Res.) Sept. 1997

Model Applicability

Unknown features are fingerprint features in the query molecule, but not found or appearing too infrequently in the training set.

1. All properties and OPS components are within expected ranges.

Feature Contribution

Top features for positive contribution

Fingerprint	Bit/Smiles	Feature Structure	Score	Carcinogen in training set
ECFP_12	-223149939	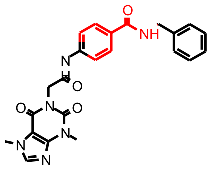 [*]NC(=O)[c]1:[cH]:[cH]:[*]:[cH]:[cH]:1	0.613	2 out of 2

ECFP_12	-177077903	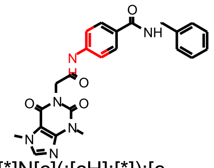 [*]N[c](:[cH]:[*]):[cH]:[*]	0.529	6 out of 10
ECFP_12	-1236483485	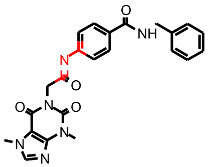 [*]C(=[*])N[c](:[*]):[*]	0.46	9 out of 17
**Top Features for negative contribution**				
**Fingerprint**	**Bit/Smiles**	**Feature Structure**	**Score**	**Carcinogen in training set**
ECFP_12	497523368	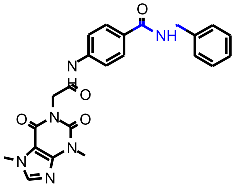 [*]CNC(=[*])[*]	-0.989	1 out of 14
ECFP_12	1571214559	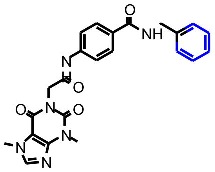 [*]1:[cH]:[cH]:[cH]:[cH]:[cH]:1	-0.56	11 out of 64
ECFP_12	-281505363	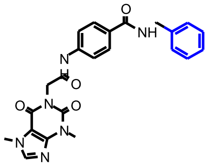 [*][c]1:[cH]:[cH]:[cH]:[cH]:[cH]:1	-0.56	11 out of 64

Sorafenib

TOPKAT_Rat_Female_FDA_None_vs_Carcinogen

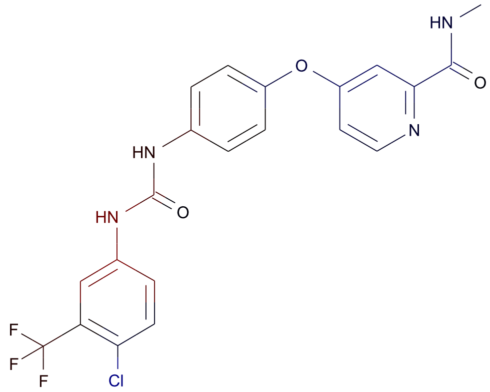

$C_{21}H_{16}ClF_3N_4O_3$

Molecular Weight: 464.82494

ALogP: 4.175

Rotatable Bonds: 6

Acceptors: 4

Donors: 3

Model Prediction

Prediction: Non-Carcinogen

Probability: 0.236

Enrichment: 0.734

Bayesian Score: -3.76

Mahalanobis Distance: 12.2

Mahalanobis Distance p-value: 0.00229

Prediction: Positive if the Bayesian score is above the estimated best cutoff value from minimizing the false positive and false negative rate.
Probability: The esimated probability that the sample is in the positive category. This assumes that the Bayesian score follows a normal distribution and is different from the prediction using a cutoff.
Enrichment: An estimate of enrichment, that is, the increased likelihood (versus random) of this sample being in the category.
Bayesian Score: The standard Laplacian-modified Bayesian score.
Mahalanobis Distance: The Mahalanobis distance (MD) is the distance to the center of the training data. The larger the MD, the less trustworthy the prediction.
Mahalanobis Distance p-value: The p-value gives the fraction of training data with an MD greater than or equal to the one for the given sample, assuming normally distributed data. The smaller the p-value, the less trustworthy the prediciton. For highly non-normal X properties (e.g., fingerprints), the MD p-value is wildly inaccurate.

Structural Similar Compounds

Name	Glimepride	Glyburide	Fluvastatin
Structure	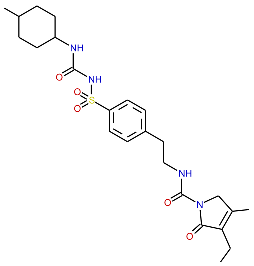	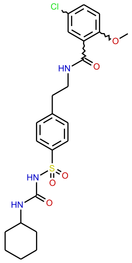	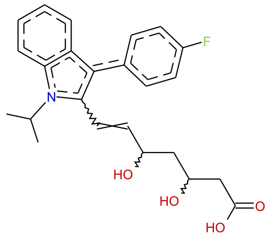
Actual Endpoint	Non-Carcinogen	Non-Carcinogen	Non-Carcinogen
Predicted Endpoint	Non-Carcinogen	Non-Carcinogen	Non-Carcinogen
Distance	0.620	0.635	0.635
Reference	US FDA (Centre for Drug Eval.& Res./Off. Testing & Res.) Sept. 1997	US FDA (Centre for Drug Eval.& Res./Off. Testing & Res.) Sept. 1997	US FDA (Centre for Drug Eval.& Res./Off. Testing & Res.) Sept. 1997

Model Applicability

Unknown features are fingerprint features in the query molecule, but not found or appearing too infrequently in the training set.

1. All properties and OPS components are within expected ranges.

Feature Contribution

Top features for positive contribution

Fingerprint	Bit/Smiles	Feature Structure	Score	Carcinogen in training set
ECFP_12	-970385855	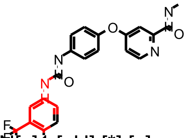 [*]N[c]1:[cH]:[*]:[c]([*]):[c](:[cH]:1)C([*])([*])[*]	0.613	2 out of 2

ECFP_12	-177077903	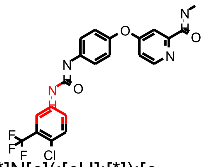 [*]N[c](:[cH]:[*]):[cH]:[*]	0.529	6 out of 10
ECFP_12	-1236483485	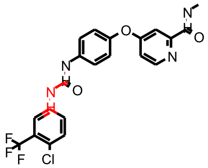 [*]C(=[*])N[c](:[*]):[*]	0.46	9 out of 17

Top Features for negative contribution

Fingerprint	Bit/Smiles	Feature Structure	Score	Carcinogen in training set
ECFP_12	1335691903	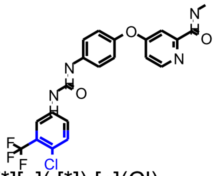 [*][c](:[*]):[c](Cl):[cH]:[*]	-1.11	2 out of 26
ECFP_12	99947387	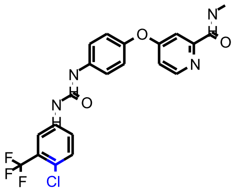 [*]:[c](:[*])Cl	-0.817	8 out of 62
ECFP_12	1413420509	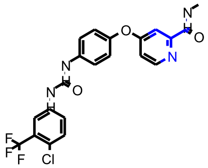 [*]C(=[*])[c](:[cH]:[*]):n:[*]	-0.661	0 out of 3

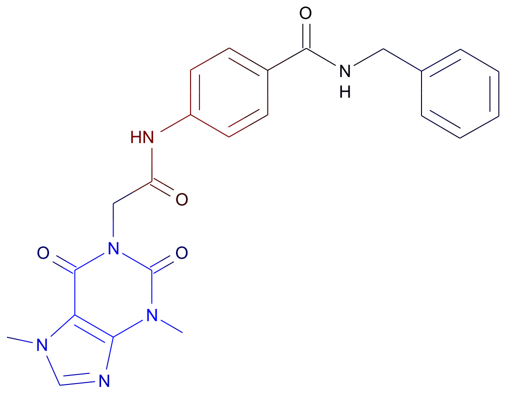

$C_{23}H_{22}N_6O_4$

Molecular Weight: 446.45858

ALogP: 1.328

Rotatable Bonds: 6

Acceptors: 5

Donors: 2

Model Prediction

Prediction: Non-Carcinogen

Probability: 0.219

Enrichment: 0.654

Bayesian Score: -6.14

Mahalanobis Distance: 16

Mahalanobis Distance p-value: 6.34e-009

Prediction: Positive if the Bayesian score is above the estimated best cutoff value from minimizing the false positive and false negative rate.
Probability: The esimated probability that the sample is in the positive category. This assumes that the Bayesian score follows a normal distribution and is different from the prediction using a cutoff.
Enrichment: An estimate of enrichment, that is, the increased likelihood (versus random) of this sample being in the category.
Bayesian Score: The standard Laplacian-modified Bayesian score.
Mahalanobis Distance: The Mahalanobis distance (MD) is the distance to the center of the training data. The larger the MD, the less trustworthy the prediction.
Mahalanobis Distance p-value: The p-value gives the fraction of training data with an MD greater than or equal to the one for the given sample, assuming normally distributed data. The smaller the p-value, the less trustworthy the prediciton. For highly non-normal X properties (e.g., fingerprints), the MD p-value is wildly inaccurate.

Structural Similar Compounds

Name	Bicalutamide	Glipizide	Acetohexamide
Structure	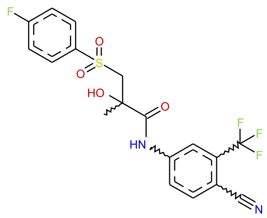	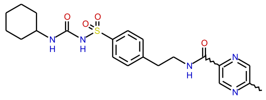	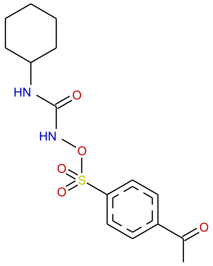
Actual Endpoint	Carcinogen	Non-Carcinogen	Non-Carcinogen
Predicted Endpoint	Carcinogen	Non-Carcinogen	Non-Carcinogen
Distance	0.598	0.637	0.684
Reference	US FDA (Centre for Drug Eval.& Res./Off. Testing & Res.) Sept. 1997	US FDA (Centre for Drug Eval.& Res./Off. Testing & Res.) Sept. 1997	US FDA (Centre for Drug Eval.& Res./Off. Testing & Res.) Sept. 1997

Model Applicability

Unknown features are fingerprint features in the query molecule, but not found or appearing too infrequently in the training set.

1. All properties and OPS components are within expected ranges.

Feature Contribution

Top features for positive contribution

Fingerprint	Bit/Smiles	Feature Structure	Score	Carcinogen in training set
SCFP_6	-347048986	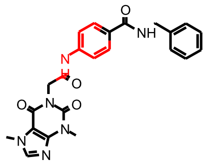 [*]C(=[*])N[c]1:[cH]:[cH]:[*]:[cH]:[cH]:1	0.615	5 out of 7

SCFP_6	814408713	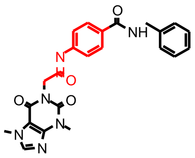<br>[*]CC(=O)N[c]1:[cH]:[cH]:[c]([*]):[cH]:[cH]:1	0.603	2 out of 2
SCFP_6	2097618059	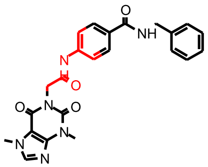<br>[*]CC(=O)N[c](:[cH]:[*]):[cH]:[*]	0.437	7 out of 13

Top Features for negative contribution

Fingerprint	Bit/Smiles	Feature Structure	Score	Carcinogen in training set
SCFP_6	399659969	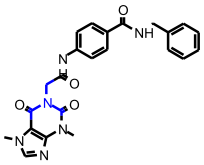<br>[*]CN(C(=[*])[*])C(=[*])[*]	-0.578	1 out of 8
SCFP_6	1653911926	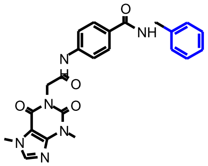<br>[*][c]1:[cH]:[cH]:[cH]:[cH]:[cH]:1	-0.504	12 out of 64
SCFP_6	1731225349	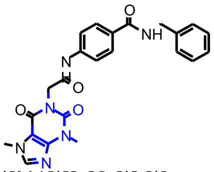<br>[*]N1[*][c]2:[*]:[*]:n:[c]:2N(C)C1=O	-0.496	0 out of 2

Sorafenib

TOPKAT_Rat_Male_FDA_None_vs_Carcinogen

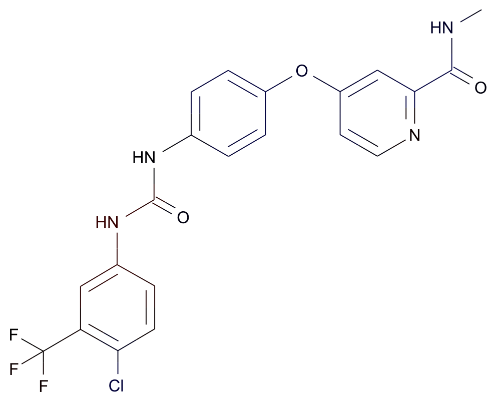

$C_{21}H_{16}ClF_3N_4O_3$

Molecular Weight: 464.82494

ALogP: 4.175

Rotatable Bonds: 6

Acceptors: 4

Donors: 3

Model Prediction

Prediction: Non-Carcinogen

Probability: 0.293

Enrichment: 0.878

Bayesian Score: -2.4

Mahalanobis Distance: 17.6

Mahalanobis Distance p-value: 1.1e-012

Prediction: Positive if the Bayesian score is above the estimated best cutoff value from minimizing the false positive and false negative rate.
Probability: The esimated probability that the sample is in the positive category. This assumes that the Bayesian score follows a normal distribution and is different from the prediction using a cutoff.
Enrichment: An estimate of enrichment, that is, the increased likelihood (versus random) of this sample being in the category.
Bayesian Score: The standard Laplacian-modified Bayesian score.
Mahalanobis Distance: The Mahalanobis distance (MD) is the distance to the center of the training data. The larger the MD, the less trustworthy the prediction.
Mahalanobis Distance p-value: The p-value gives the fraction of training data with an MD greater than or equal to the one for the given sample, assuming normally distributed data. The smaller the p-value, the less trustworthy the prediciton. For highly non-normal X properties (e.g., fingerprints), the MD p-value is wildly inaccurate.

Structural Similar Compounds

Name	Glyburide	Glimepride	Fluvastatin
Structure	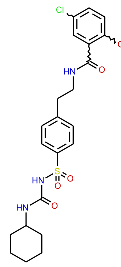	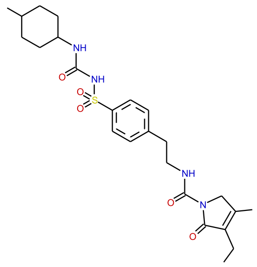	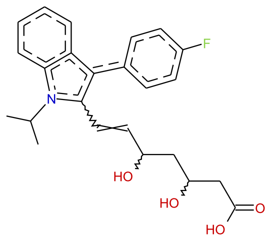
Actual Endpoint	Non-Carcinogen	Non-Carcinogen	Carcinogen
Predicted Endpoint	Non-Carcinogen	Non-Carcinogen	Carcinogen
Distance	0.593	0.600	0.615
Reference	US FDA (Centre for Drug Eval.& Res./Off. Testing & Res.) Sept. 1997	US FDA (Centre for Drug Eval.& Res./Off. Testing & Res.) Sept. 1997	US FDA (Centre for Drug Eval.& Res./Off. Testing & Res.) Sept. 1997

Model Applicability

Unknown features are fingerprint features in the query molecule, but not found or appearing too infrequently in the training set.

1. All properties and OPS components are within expected ranges.

Feature Contribution

Top features for positive contribution				
**Fingerprint**	**Bit/Smiles**	**Feature Structure**	**Score**	**Carcinogen in training set**
SCFP_6	-347048986	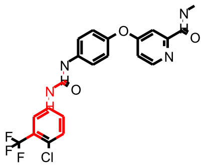 [*]C(=[*])N[c]1:[cH]:[cH]:[*]:[cH]:[cH]:1	0.615	5 out of 7

SCFP_6	-754059116	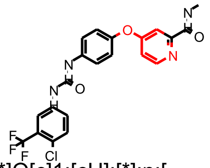 [*]O[c]1:[cH]:[*]:n:[cH]:[cH]:1	0.415	1 out of 1
SCFP_6	-531283893	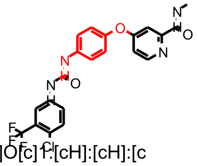 [*]O[c]1:[cH]:[cH]:[c](NC(=[*])[*]):[cH]:[cH]:1	0.273	2 out of 4

Top Features for negative contribution

Fingerprint	Bit/Smiles	Feature Structure	Score	Carcinogen in training set
SCFP_6	-827073191	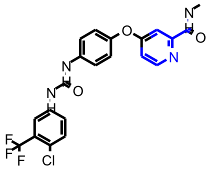 [*]C(=[*])[c]1:[cH]:[*]:[cH]:[cH]:n:1	-0.674	0 out of 3
SCFP_6	-488587948	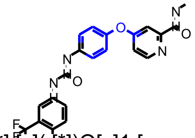 [*]:[c](:[*])O[c]1:[cH]:[cH]:[*]:[cH]:[cH]:1	-0.496	0 out of 2
SCFP_6	-975241316	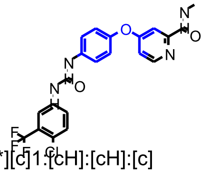 [*]:[c]1:[cH]:[cH]:[c](O[c](:[cH]:[*]):[cH]:[*]):[cH]:[cH]:1	-0.496	0 out of 2

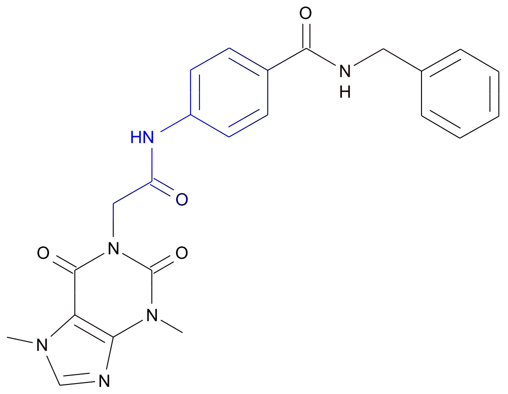

$C_{23}H_{22}N_6O_4$

Molecular Weight: 446.45858

ALogP: 1.328

Rotatable Bonds: 6

Acceptors: 5

Donors: 2

Model Prediction

Prediction: Non-Irritant

Probability: 0.487

Enrichment: 0.528

Bayesian Score: -4.59

Mahalanobis Distance: 11.4

Mahalanobis Distance p-value: 0.000528

Prediction: Positive if the Bayesian score is above the estimated best cutoff value from minimizing the false positive and false negative rate.
Probability: The esimated probability that the sample is in the positive category. This assumes that the Bayesian score follows a normal distribution and is different from the prediction using a cutoff.
Enrichment: An estimate of enrichment, that is, the increased likelihood (versus random) of this sample being in the category.
Bayesian Score: The standard Laplacian-modified Bayesian score.
Mahalanobis Distance: The Mahalanobis distance (MD) is the distance to the center of the training data. The larger the MD, the less trustworthy the prediction.
Mahalanobis Distance p-value: The p-value gives the fraction of training data with an MD greater than or equal to the one for the given sample, assuming normally distributed data. The smaller the p-value, the less trustworthy the prediciton. For highly non-normal X properties (e.g., fingerprints), the MD p-value is wildly inaccurate.

Structural Similar Compounds

Name	2-Anthracenesulfonic acid, 1-amino-9,10-dihydro-9,10-dioxo-4-(2,4,6 -trimethylanilino)-, monosodium salt	Pregna-1,4-diene-3,20-dione, 21-(acetyloxy)-11-hydroxy-6-methyl-17- (1-oxopropoxy)-, (6-alpha,11-beta)-	5-Norbornene-2,3-dicarboxylic acid, 1,4,5,6,7,7-hexachloro-
Structure	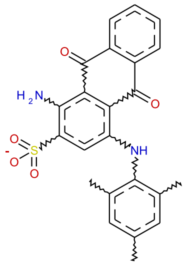	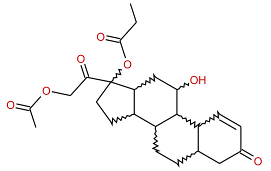	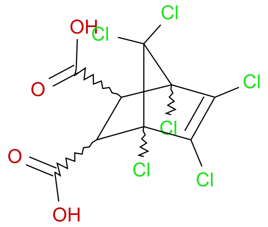
Actual Endpoint	Irritant	Irritant	Irritant
Predicted Endpoint	Non-Irritant	Irritant	Irritant
Distance	0.758	0.785	0.830
Reference	85JCAE "Prehled Prumyslove Toxikologie; Organicke Latky," Marhold, J., Prague , Czechoslovakia, Avicenum, 1986 Volume(issue)/page/year: -,1327,1986	YACHDS Yakuri to Chiryo. Pharmacology and Therapeutics. (Raifu Saiensu Shup pan K.K., 2-5-13, Yaesu, Chuo-ku, Tokyo 104, Japan) V.1- 1972- Volume(issue) /page/year: 19,3103,1991	85JCAE "Prehled Prumyslove Toxikologie; Organicke Latky," Marhold, J., Prague , Czechoslovakia, Avicenum, 1986 Volume(issue)/page/year: -,581,1986

Model Applicability

Unknown features are fingerprint features in the query molecule, but not found or appearing too infrequently in the training set.

1. All properties and OPS components are within expected ranges.
2. Unknown FCFP_2 feature: 136150461: [*]:n(:[*])C

Feature Contribution

Top features for positive contribution

Fingerprint	Bit/Smiles	Feature Structure	Score	Irritant in training set

FCFP_12	-1986158408	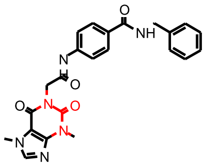 [*]N([*])C(=O)N([*])[*]	0.0821	13 out of 13
FCFP_12	-1539132615	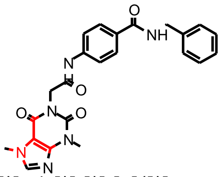 [*]n1:[*]:[*]:[c]([*]):[c]:1C(=[*])[*]	0.0795	9 out of 9
FCFP_12	907096426	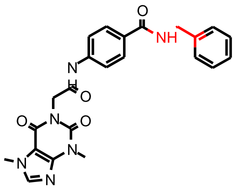 [*]NC[c](:[*]):[*]	0.0772	7 out of 7
**Top Features for negative contribution**				
**Fingerprint**	**Bit/Smiles**	**Feature Structure**	**Score**	**Irritant in training set**
FCFP_12	1175665944	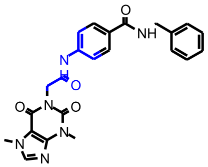 [*]CC(=O)N[c](:[cH]:[*]):[cH]:[*]	-1.02	2 out of 8
FCFP_12	-1838187238	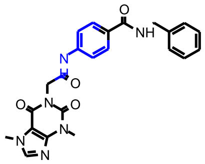 [*]C(=[*])N[c]1:[cH]: [cH]:[*]:[cH]:[cH]:1	-0.692	5 out of 12

FCFP_12	-451043714	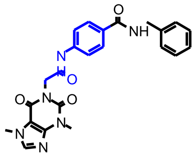 [*]CC(=O)N[c]1:[cH]:[cH]:[c]([*]):[cH]:[cH]:1	-0.65	0 out of 1

Sorafenib

TOPKAT_Skin_Irritancy_None_vs_Irritant

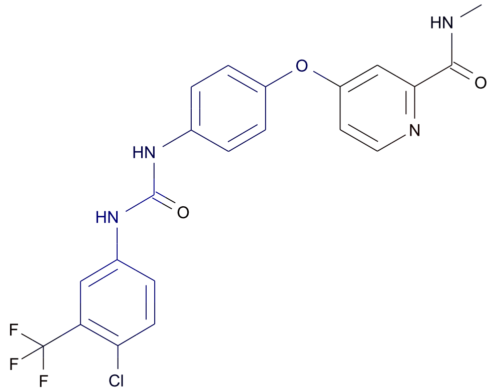

$C_{21}H_{16}ClF_3N_4O_3$

Molecular Weight: 464.82494

ALogP: 4.175

Rotatable Bonds: 6

Acceptors: 4

Donors: 3

Model Prediction

Prediction: Non-Irritant

Probability: 0.264

Enrichment: 0.287

Bayesian Score: -5.23

Mahalanobis Distance: 8.27

Mahalanobis Distance p-value: 0.791

Prediction: Positive if the Bayesian score is above the estimated best cutoff value from minimizing the false positive and false negative rate.
Probability: The esimated probability that the sample is in the positive category. This assumes that the Bayesian score follows a normal distribution and is different from the prediction using a cutoff.
Enrichment: An estimate of enrichment, that is, the increased likelihood (versus random) of this sample being in the category.
Bayesian Score: The standard Laplacian-modified Bayesian score.
Mahalanobis Distance: The Mahalanobis distance (MD) is the distance to the center of the training data. The larger the MD, the less trustworthy the prediction.
Mahalanobis Distance p-value: The p-value gives the fraction of training data with an MD greater than or equal to the one for the given sample, assuming normally distributed data. The smaller the p-value, the less trustworthy the prediciton. For highly non-normal X properties (e.g., fingerprints), the MD p-value is wildly inaccurate.

Structural Similar Compounds

Name	5-Norbornene-2,3-dicarboxylic acid, 1,4,5,6,7,7-hexachloro-	Benzenesulfonic acid, 2,2'-(4,4'-biphenylylenedivinylene)di-, disod ium salt	Sulfide, bis(4-t-butyl-m-cresyl)-
Structure	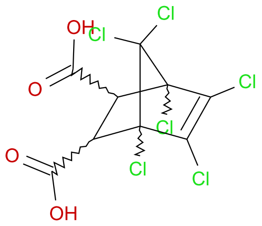	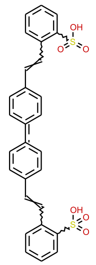	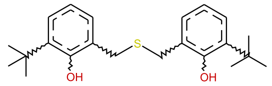
Actual Endpoint	Irritant	Irritant	Irritant
Predicted Endpoint	Irritant	Non-Irritant	Irritant
Distance	0.844	0.871	0.884
Reference	85JCAE "Prehled Prumyslove Toxikologie; Organicke Latky," Marhold, J., Prague , Czechoslovakia, Avicenum, 1986 Volume(issue)/page/year: -,581,1986	MVCRB3 MVC-Report. (Stockholm, Sweden) No.1-2, 1972-73. Discontinued. Volu me(issue)/page/year: 2,193,1973	AMIHBC AMA Archives of Industrial Hygiene and Occupational Medicine. (Chicago , IL) V.2-10, 1950-54. For publisher information, see AEHLAU. Volume(issue)/pag e/year: 5,311,1952

Model Applicability

Unknown features are fingerprint features in the query molecule, but not found or appearing too infrequently in the training set.

1. All properties and OPS components are within expected ranges.

Feature Contribution

Top features for positive contribution				
Fingerprint	Bit/Smiles	Feature Structure	Score	Irritant in training set

FCFP_12	-124655670	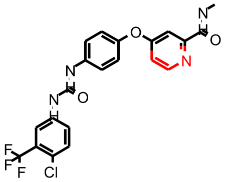 [*]:[cH]:[cH]:n:[*]	0.0821	13 out of 13
FCFP_12	-1539132615	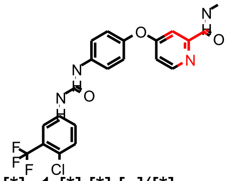 [*]n1:[*]:[*]:[c]([*]):[c]:1C(=[*])[*]	0.0795	9 out of 9
FCFP_12	-1695756380	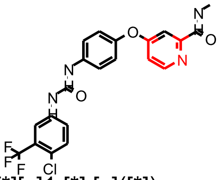 [*][c]1:[*]:[c]([*]): n:[cH]:[cH]:1	0.0772	7 out of 7
**Top Features for negative contribution**				
**Fingerprint**	**Bit/Smiles**	**Feature Structure**	**Score**	**Irritant in training set**
FCFP_12	-789307649	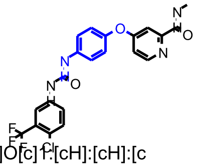 [*]O[c]1:[cH]:[cH]:[c](NC(=[*])[*]):[cH]: [cH]:1	-1.54	0 out of 4
FCFP_12	-1838187238	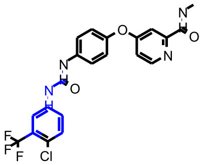 [*]C(=[*])N[c]1:[cH]: [cH]:[*]:[cH]:[cH]:1	-0.692	5 out of 12

FCFP_12	1294255210	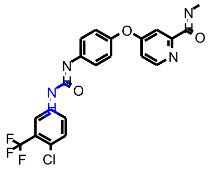 [*]C(=[*])N[c](:[*]):[*]	-0.486	12 out of 22

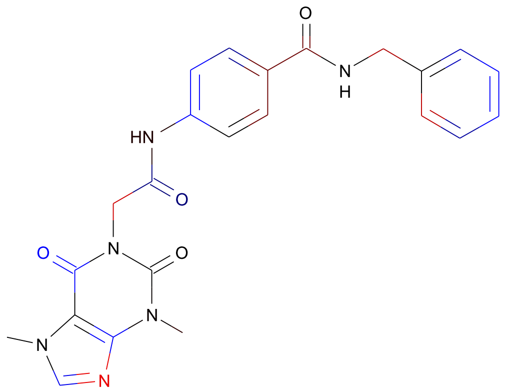

$C_{23}H_{22}N_6O_4$
Molecular Weight: 446.45858
ALogP: 1.328
Rotatable Bonds: 6
Acceptors: 5
Donors: 2

Model Prediction

Prediction: 18.7
Unit: mg/kg_body_weight/day
Mahalanobis Distance: 12.2
Mahalanobis Distance p-value: 7.09e-006

Mahalanobis Distance: The Mahalanobis distance (MD) is a generalization of the Euclidean distance that accounts for correlations among the X properties. It is calculated as the distance to the center of the training data. The larger the MD, the less trustworthy the prediction.
Mahalanobis Distance p-value: The p-value gives the fraction of training data with an MD greater than or equal to the one for the given sample, assuming normally distributed data. The smaller the p-value, the less trustworthy the prediciton. For highly non-normal X properties (e.g., fingerprints), the MD p-value is wildly inaccurate.

Structural Similar Compounds

Name	Ochratoxin A	542	470
Structure	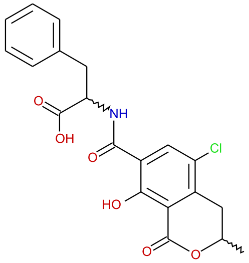	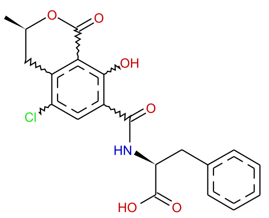	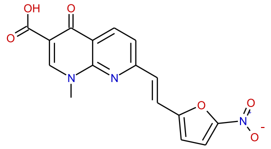
Actual Endpoint (-log C)	4.79932	4.79932	4.62839
Predicted Endpoint (-log C)	3.6353	3.6353	3.93264
Distance	0.719	0.719	0.782
Reference	CPDB	CPDB	CPDB

Model Applicability

Unknown features are fingerprint features in the query molecule, but not found or appearing too infrequently in the training set.

1. All properties and OPS components are within expected ranges.
2. Unknown ECFP_2 feature: -960717516: [*]C(=[*])N(C)[c](:[*]):[*]
3. Unknown ECFP_2 feature: -661097313: [*]CN(C(=[*])[*])C(=[*])[*]
4. Unknown ECFP_2 feature: 1135573248: [*]N([*])C(=O)N([*])[*]
5. Unknown ECFP_2 feature: -37698365: [*]N([*])CC(=[*])[*]

Feature Contribution

Top features for positive contribution

Fingerprint	Bit/Smiles	Feature Structure	Score
ECFP_6	655739385	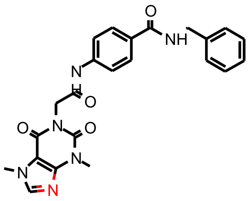 [*]:n:[*]	0.229

ECFP_6	1559650422	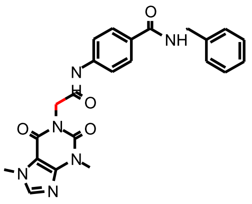 [*]C[*]	0.203
ECFP_6	-2024255407	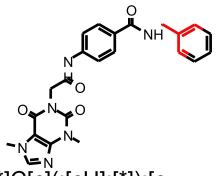 [*]C[c](:[cH]:[*]):[c H]:[*]	0.172
**Top Features for negative contribution**			
**Fingerprint**	**Bit/Smiles**	**Feature Structure**	**Score**
ECFP_6	2106656448	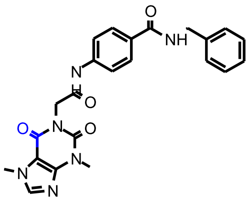 [*]C(=O)[*]	-0.275
ECFP_6	1996767644	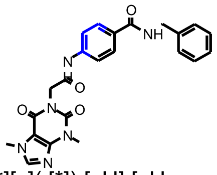 [*][c](:[*]):[cH]:[cH]:[*]	-0.251
ECFP_6	642810091	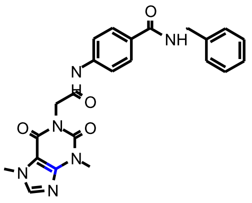 [*][c](:[*]):[*]	-0.247

Sorafenib

TOPKAT_Carcinogenic_Potency_TD50_Mouse

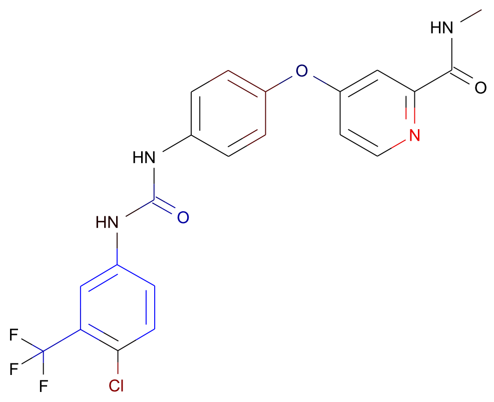

$C_{21}H_{16}ClF_3N_4O_3$
Molecular Weight: 464.82494
ALogP: 4.175
Rotatable Bonds: 6
Acceptors: 4
Donors: 3

Structural Similar Compounds

Name	Ochratoxin A	542	4-Chloro-6-(2,3-xylidino)-2-pyri-mi-dinylthio(N-b-hydroxy-ethyl) acetamide
Structure	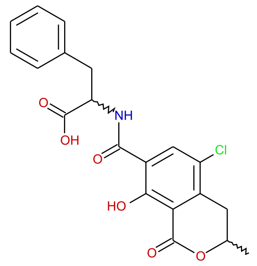	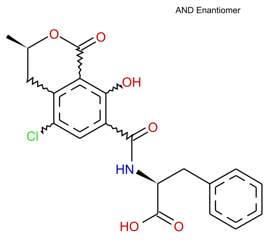	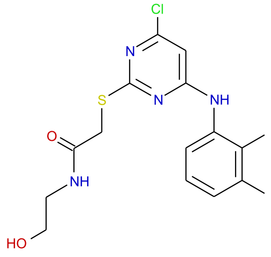
Actual Endpoint (-log C)	4.79932	4.79932	3.91517
Predicted Endpoint (-log C)	3.6353	3.6353	3.92186
Distance	0.718	0.718	0.738
Reference	CPDB	CPDB	CPDB

Model Prediction

Prediction: 19.2
Unit: mg/kg_body_weight/day
Mahalanobis Distance: 12.4
Mahalanobis Distance p-value: 2.94e-006

Mahalanobis Distance: The Mahalanobis distance (MD) is a generalization of the Euclidean distance that accounts for correlations among the X properties. It is calculated as the distance to the center of the training data. The larger the MD, the less trustworthy the prediction.
Mahalanobis Distance p-value: The p-value gives the fraction of training data with an MD greater than or equal to the one for the given sample, assuming normally distributed data. The smaller the p-value, the less trustworthy the prediciton. For highly non-normal X properties (e.g., fingerprints), the MD p-value is wildly inaccurate.

Model Applicability

Unknown features are fingerprint features in the query molecule, but not found or appearing too infrequently in the training set.

1. All properties and OPS components are within expected ranges.
2. Unknown ECFP_2 feature: 1338334141: [*]C(=[*])NC
3. Unknown ECFP_2 feature: 1413420509: [*]C(=[*])[c](:n:[*]):c:[*]

Feature Contribution

Top features for positive contribution

Fingerprint	Bit/Smiles	Feature Structure	Score
ECFP_6	655739385	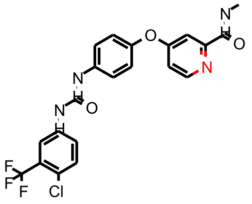 [*]:n:[*]	0.229

ECFP_6	-817402818	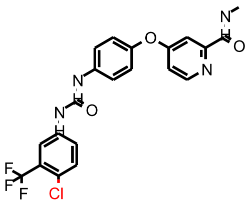 [*]Cl	0.129
ECFP_6	-176455838	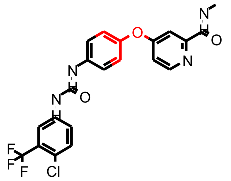 [*]O[c](:[cH]:[*]):[cH]:[*]	0.0818
**Top Features for negative contribution**			
**Fingerprint**	**Bit/Smiles**	**Feature Structure**	**Score**
ECFP_6	1996767644	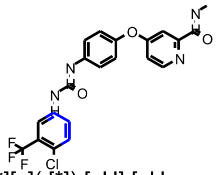 [*][c](:[*]):[cH]:[cH]:[*]	-0.251
ECFP_6	642810091	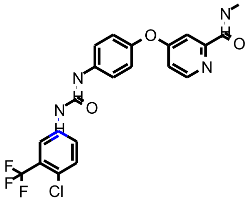 [*][c](:[*]):[*]	-0.247
ECFP_6	-182236392	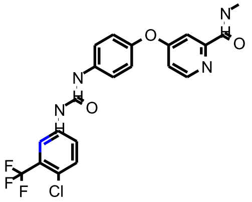 [*]:[cH]:[*]	-0.232

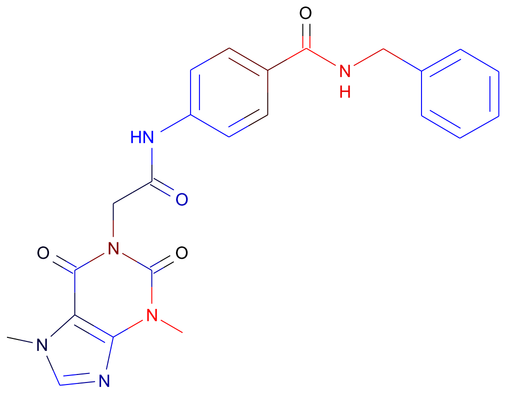

$C_{23}H_{22}N_6O_4$
Molecular Weight: 446.45858
ALogP: 1.328
Rotatable Bonds: 6
Acceptors: 5
Donors: 2

Model Prediction

Prediction: 33.3
Unit: mg/kg_body_weight/day
Mahalanobis Distance: 16.4
Mahalanobis Distance p-value: 1.21e-015

Mahalanobis Distance: The Mahalanobis distance (MD) is a generalization of the Euclidean distance that accounts for correlations among the X properties. It is calculated as the distance to the center of the training data. The larger the MD, the less trustworthy the prediction.
Mahalanobis Distance p-value: The p-value gives the fraction of training data with an MD greater than or equal to the one for the given sample, assuming normally distributed data. The smaller the p-value, the less trustworthy the prediciton. For highly non-normal X properties (e.g., fingerprints), the MD p-value is wildly inaccurate.

Structural Similar Compounds

Name	4-Bis(2-hydroxyethyl)amino-2-(5-nitro-2-thienyl)quinazoline	Ochratoxin A	542
Structure	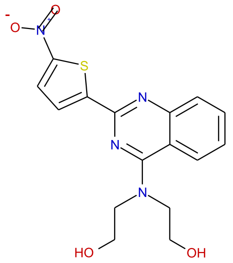	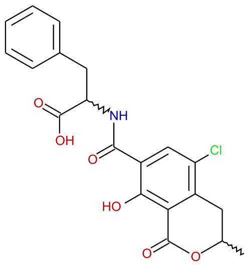	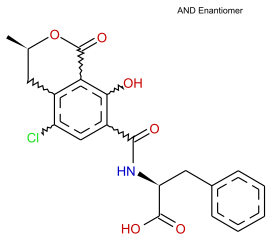
Actual Endpoint (-log C)	5.05984	6.47264	6.59334
Predicted Endpoint (-log C)	4.23808	5.06501	5.06501
Distance	0.723	0.731	0.731
Reference	CPDB	CPDB	CPDB

Model Applicability

Unknown features are fingerprint features in the query molecule, but not found or appearing too infrequently in the training set.

1. All properties and OPS components are within expected ranges.

Feature Contribution

Top features for positive contribution

Fingerprint	Bit/Smiles	Feature Structure	Score
FCFP_6	136627117	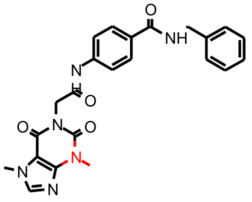 [*]N([*])C	0.69

FCFP_6	1	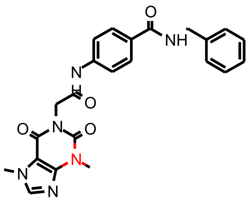<br>[*]N([*])[*]	0.234
FCFP_6	-885550502	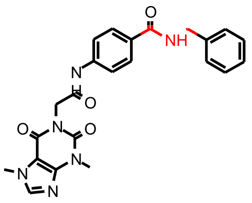<br>[*]CNC(=[*])[*]	0.229
**Top Features for negative contribution**			
**Fingerprint**	**Bit/Smiles**	**Feature Structure**	**Score**
FCFP_6	991735244	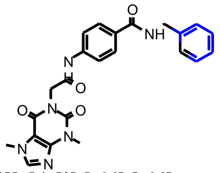<br>[*][c]1:[*]:[cH]:[cH]<br>:[cH]:[cH]:1	-0.422
FCFP_6	-2093839777	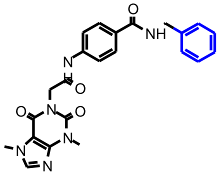<br>[*][c]1:[cH]:[cH]:[cH<br>]:[cH]:[cH]:1	-0.378
FCFP_6	16	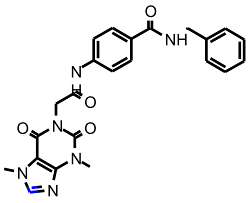<br>[*]:[cH]:[*]	-0.354

Sorafenib

TOPKAT_Carcinogenic_Potency_TD50_Rat

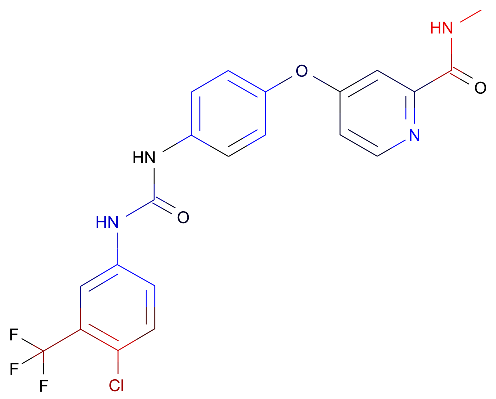

$C_{21}H_{16}ClF_3N_4O_3$
Molecular Weight: 464.82494
ALogP: 4.175
Rotatable Bonds: 6
Acceptors: 4
Donors: 3

Model Prediction

Prediction: 14.2
Unit: mg/kg_body_weight/day
Mahalanobis Distance: 20.4
Mahalanobis Distance p-value: 9.56e-031

Mahalanobis Distance: The Mahalanobis distance (MD) is a generalization of the Euclidean distance that accounts for correlations among the X properties. It is calculated as the distance to the center of the training data. The larger the MD, the less trustworthy the prediction.
Mahalanobis Distance p-value: The p-value gives the fraction of training data with an MD greater than or equal to the one for the given sample, assuming normally distributed data. The smaller the p-value, the less trustworthy the prediciton. For highly non-normal X properties (e.g., fingerprints), the MD p-value is wildly inaccurate.

Structural Similar Compounds

Name	Fluvastatin	913	Ochratoxin A
Structure	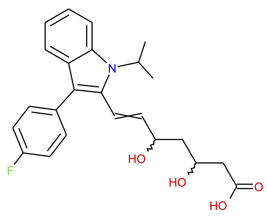	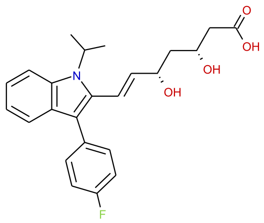	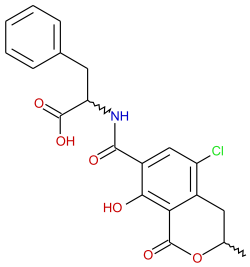
Actual Endpoint (-log C)	3.51742	3.51742	6.47264
Predicted Endpoint (-log C)	5.41573	5.41573	5.06501
Distance	0.597	0.597	0.666
Reference	CPDB	CPDB	CPDB

Model Applicability

Unknown features are fingerprint features in the query molecule, but not found or appearing too infrequently in the training set.

1. All properties and OPS components are within expected ranges.
2. Unknown FCFP_2 feature: -1029533685: [*]:[c](:[*])C(F)(F)F

Feature Contribution

Top features for positive contribution			
**Fingerprint**	**Bit/Smiles**	**Feature Structure**	**Score**
FCFP_6	1	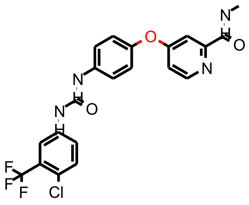 [*]N([*])[*]	0.234

FCFP_6	-885550502	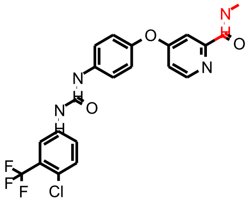<br>[*]CNC(=[*])[*]	0.229
FCFP_6	32	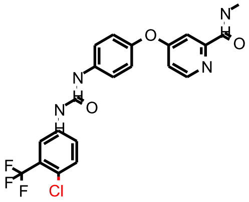<br>[*]Cl	0.154

Top Features for negative contribution

Fingerprint	Bit/Smiles	Feature Structure	Score
FCFP_6	16	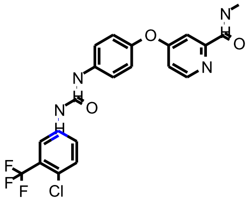<br>[*]:[cH]:[*]	-0.354
FCFP_6	590925877	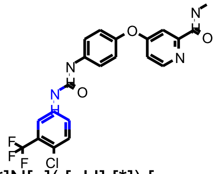<br>[*]N[c](:[cH]:[*]):[cH]:[*]	-0.323
FCFP_6	1674451008	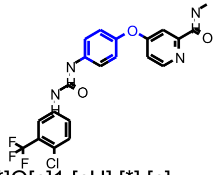<br>[*]O[c]1:[cH]:[*]:[c]([*]):[cH]:[cH]:1	-0.233

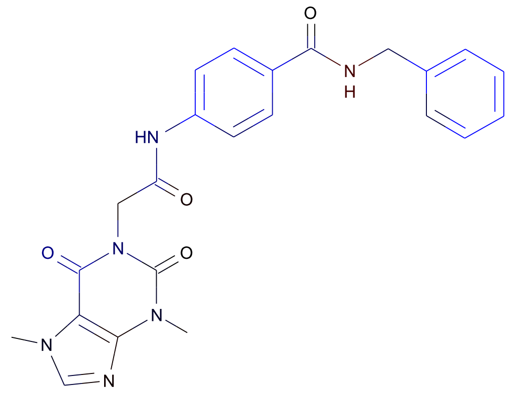

$C_{23}H_{22}N_6O_4$
Molecular Weight: 446.45858
ALogP: 1.328
Rotatable Bonds: 6
Acceptors: 5
Donors: 2

Model Prediction

Prediction: 0.0377
Unit: g/kg_body_weight
Mahalanobis Distance: 30.7
Mahalanobis Distance p-value: 5e-026

Mahalanobis Distance: The Mahalanobis distance (MD) is a generalization of the Euclidean distance that accounts for correlations among the X properties. It is calculated as the distance to the center of the training data. The larger the MD, the less trustworthy the prediction.
Mahalanobis Distance p-value: The p-value gives the fraction of training data with an MD greater than or equal to the one for the given sample, assuming normally distributed data. The smaller the p-value, the less trustworthy the prediciton. For highly non-normal X properties (e.g., fingerprints), the MD p-value is wildly inaccurate.

Structural Similar Compounds

Name	GLIPIZIDE	CHLORSULFURON	DANTROLENE.NA
Structure	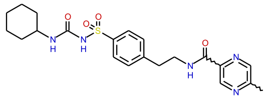	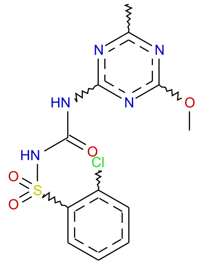	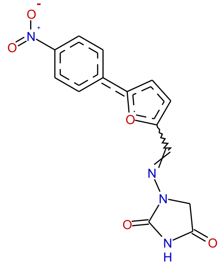
Actual Endpoint (-log C)	3.94991	4.15566	4.19625
Predicted Endpoint (-log C)	3.95594	3.79771	4.62637
Distance	0.639	0.723	0.727
Reference	NDA-17583	EPA COVER SHEET 0027;880301;(1)	NDA-17443

Model Applicability

Unknown features are fingerprint features in the query molecule, but not found or appearing too infrequently in the training set.

1. All properties and OPS components are within expected ranges.
2. Unknown ECFP_6 feature: 672362763: [*]n(:[*]):[*]
3. Unknown ECFP_6 feature: -677309799: [*][c]1:[*]:[*]:[cH]:n:1
4. Unknown ECFP_6 feature: -708878603: [*]n1:[*]:[*]:n:[cH]:1
5. Unknown ECFP_6 feature: -407983022: [*][c]1:[*]:[*]:[cH]:n:1C
6. Unknown ECFP_6 feature: -960717516: [*]C(=[*])N(C)[c](:[*]):[*]
7. Unknown ECFP_6 feature: -509950643: [*]N([*])[c]1:n:[*]:[*]:[c]:1[*]
8. Unknown ECFP_6 feature: -813242890: [*]n1:[*]:[*]:[c]([*]):[c]:1C(=[*])[*]
9. Unknown ECFP_6 feature: 1945129186: [*]N([*])C(=O)[c](:[*]):[*]
10. Unknown ECFP_6 feature: -661097313: [*]CN(C(=[*])[*])C(=[*])[*]
11. Unknown ECFP_6 feature: 1135573248: [*]N([*])C(=O)N([*])[*]
12. Unknown ECFP_6 feature: -37698365: [*]N([*])CC(=[*])[*]
13. Unknown ECFP_6 feature: 1731843802: [*]CC(=O)N[*]
14. Unknown ECFP_6 feature: -177077903: [*]N[c](:[cH]:[*]):[cH]:[*]
15. Unknown ECFP_6 feature: 866343404: [*]N([*])C
16. Unknown ECFP_6 feature: 866450950: [*]:n(:[*])C
17. Unknown ECFP_6 feature: -175146122: [*]C(=[*])[c](:[cH]:[*]):[cH]:[*]
18. Unknown ECFP_6 feature: 1430169877: [*]NC(=O)[c](:[*]):[*]
19. Unknown ECFP_6 feature: 497523368: [*]CNC(=[*])[*]

20. Unknown ECFP_6 feature: 769925792: [*]NC[c](:[*]):[*]
21. Unknown ECFP_6 feature: 1997021792: [*]:[cH]:[cH]:[cH]:[*]

Feature Contribution

Top features for positive contribution			
**Fingerprint**	**Bit/Smiles**	**Feature Structure**	**Score**
ECFP_6	1559650422	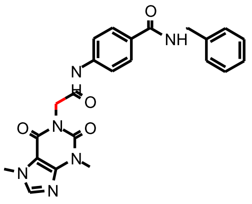 [*]C[*]	0.129
FCFP_6	3	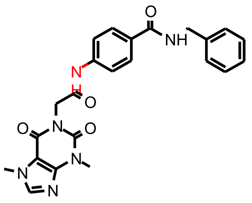 [*]N[*]	0.0924
FCFP_6	-2093839777	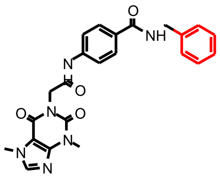 [*][c]1:[cH]:[cH]:[cH]:[cH]:[cH]:1	0.078
**Top Features for negative contribution**			
**Fingerprint**	**Bit/Smiles**	**Feature Structure**	**Score**
FCFP_6	991735244	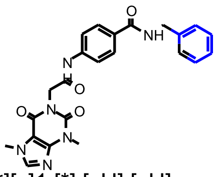 [*][c]1:[*]:[cH]:[cH]:[cH]:[cH]:1	-0.134

ECFP_6	1564392544	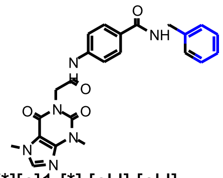 [*][c]1:[*]:[cH]:[cH]:[cH]:[cH]:1	-0.133
ECFP_6	2106656448	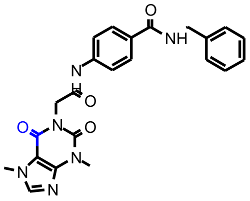 [*]C(=O)[*]	-0.11

Sorafenib

TOPKAT_Chronic_LOAEL

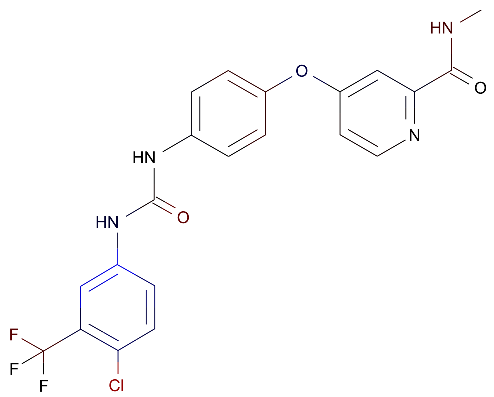

$C_{21}H_{16}ClF_3N_4O_3$

Molecular Weight: 464.82494

ALogP: 4.175

Rotatable Bonds: 6

Acceptors: 4

Donors: 3

Model Prediction

Prediction: 0.00483

Unit: g/kg_body_weight

Mahalanobis Distance: 30

Mahalanobis Distance p-value: 1.21e-024

Mahalanobis Distance: The Mahalanobis distance (MD) is a generalization of the Euclidean distance that accounts for correlations among the X properties. It is calculated as the distance to the center of the training data. The larger the MD, the less trustworthy the prediction.

Mahalanobis Distance p-value: The p-value gives the fraction of training data with an MD greater than or equal to the one for the given sample, assuming normally distributed data. The smaller the p-value, the less trustworthy the prediciton. For highly non-normal X properties (e.g., fingerprints), the MD p-value is wildly inaccurate.

Structural Similar Compounds

Name	GLYBURIDE	D & C RED 9	SODIUM ACIFLUORFEN
Structure	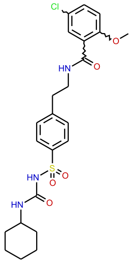	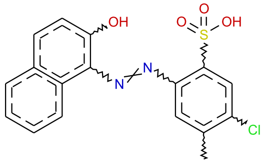	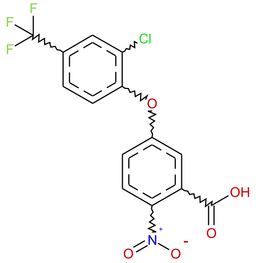
Actual Endpoint (-log C)	4.21661	3.87715	4.16036
Predicted Endpoint (-log C)	4.21035	3.6546	4.65915
Distance	0.636	0.722	0.736
Reference	UPJ-26452	NTP REPORT # 225	EPA COVER SHEET 0192;891101;(1)

Model Applicability

Unknown features are fingerprint features in the query molecule, but not found or appearing too infrequently in the training set.

1. All properties and OPS components are within expected ranges.
2. Unknown ECFP_6 feature: -1046436026: [*]F
3. Unknown ECFP_6 feature: 99947387: [*]:[c](:[*])Cl
4. Unknown ECFP_6 feature: 226796801: [*]C([*])([*])F
5. Unknown ECFP_6 feature: 1305253718: [*]:[c](:[*])O[c](:[*]):[*]
6. Unknown ECFP_6 feature: -677309799: [*][c]1:[*]:[*]:[cH]:n:1
7. Unknown ECFP_6 feature: 1338334141: [*]C(=[*])NC
8. Unknown ECFP_6 feature: -177077903: [*]N[c](:[cH]:[*]):[cH]:[*]
9. Unknown ECFP_6 feature: 1336678434: [*][c](:[*]):[c](:[cH]:[*])C([*])([*])[*]
10. Unknown ECFP_6 feature: -649580166: [*]NC(=O)N[*]
11. Unknown ECFP_6 feature: -1952889961: [*]:[c](:[*])C(F)(F)F
12. Unknown ECFP_6 feature: 1413420509: [*]C(=[*])[c](:[cH]:[*]):n:[*]
13. Unknown ECFP_6 feature: 1996163143: [*]:[cH]:[cH]:n:[*]
14. Unknown ECFP_6 feature: 1430169877: [*]NC(=O)[c](:[*]):[*]
15. Unknown ECFP_6 feature: 864287155: [*]NC

Feature Contribution

Top features for positive contribution

Fingerprint	Bit/Smiles	Feature Structure	Score
ECFP_6	-176455838	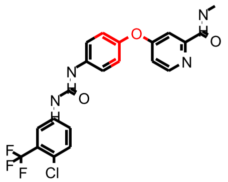 [*]O[c](:[cH]:[*]):[cH]:[*]	0.106
FCFP_6	32	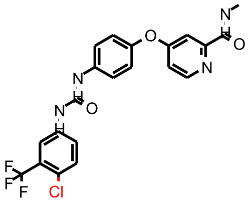 [*]Cl	0.101
FCFP_6	3	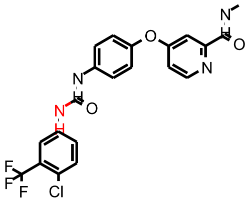 [*]N[*]	0.0924

Top Features for negative contribution

Fingerprint	Bit/Smiles	Feature Structure	Score
FCFP_6	1	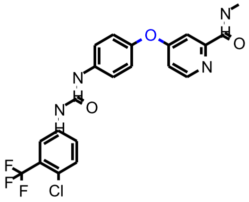 [*]N([*])[*]	-0.102

ECFP_6	-1236483485	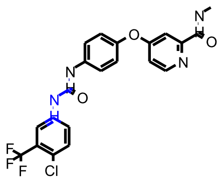 [*]C(=[*])N[c](:[*]):[*]	-0.0747
FCFP_6	203677720	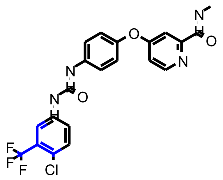 [*]C(=[*])[c](:[cH]:[*]):[cH]:[*]	-0.0713

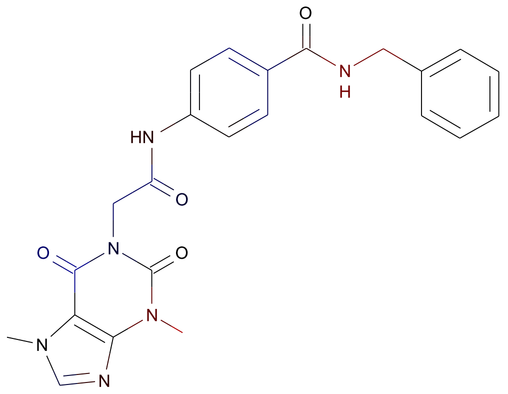

$C_{23}H_{22}N_6O_4$
Molecular Weight: 446.45858
ALogP: 1.328
Rotatable Bonds: 6
Acceptors: 5
Donors: 2

Model Prediction

Prediction: 0.0266
Unit: g/kg_body_weight
Mahalanobis Distance: 10.2
Mahalanobis Distance p-value: 2.29e-005

Mahalanobis Distance: The Mahalanobis distance (MD) is a generalization of the Euclidean distance that accounts for correlations among the X properties. It is calculated as the distance to the center of the training data. The larger the MD, the less trustworthy the prediction.
Mahalanobis Distance p-value: The p-value gives the fraction of training data with an MD greater than or equal to the one for the given sample, assuming normally distributed data. The smaller the p-value, the less trustworthy the prediciton. For highly non-normal X properties (e.g., fingerprints), the MD p-value is wildly inaccurate.

Structural Similar Compounds

Name	FUROSEMIDE	SALICYLAZOSULFAPYRIDINE	DAPSONE
Structure	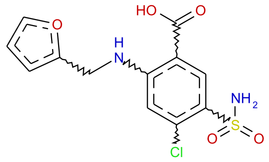	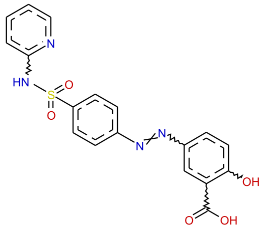	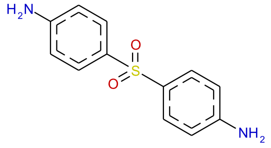
Actual Endpoint (-log C)	4.04236	3.375	3.66258
Predicted Endpoint (-log C)	2.8614	2.80292	3.26993
Distance	0.679	0.769	0.826
Reference	NCI/NTP TR-356	NCI/NTP TR-457	NCI/NTP TR-20

Model Applicability

Unknown features are fingerprint features in the query molecule, but not found or appearing too infrequently in the training set.

1. OPS PC9 out of range. Value: 3.4935. Training min, max, SD, explained variance: -2.8548, 3.3954, 1.263, 0.0360.
2. Unknown FCFP_2 feature: -124685461: [*]n1:[*]:[*]:n:[cH]:1
3. Unknown FCFP_2 feature: -306856457: [*][c]1:[*]:[*]:[cH]:n:1C
4. Unknown FCFP_2 feature: 136150461: [*]:n(:[*])C

Feature Contribution

Top features for positive contribution			
**Fingerprint**	**Bit/Smiles**	**Feature Structure**	**Score**
FCFP_2	136627117	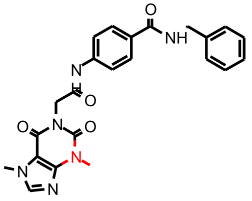 [*]N([*])C	0.173

FCFP_2	-885550502	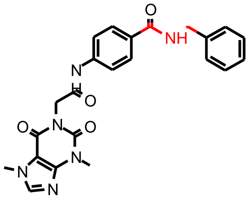 [*]CNC(=[*])[*]	0.115
FCFP_2	3	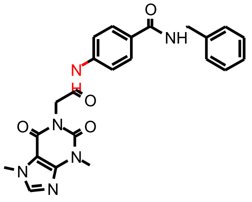 [*]N[*]	0.0737
**Top Features for negative contribution**			
**Fingerprint**	**Bit/Smiles**	**Feature Structure**	**Score**
FCFP_2	1872154524	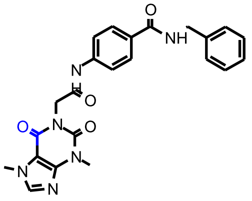 [*]C(=O)[*]	-0.105
FCFP_2	203677720	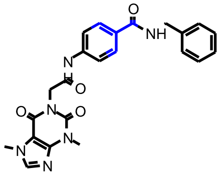 [*]C(=[*])[c](:[cH]:[*]):[cH]:[*]	-0.0829
FCFP_2	1	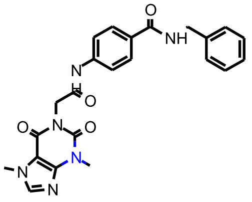 [*]N([*])[*]	-0.0796

Sorafenib

TOPKAT_Rat_Maximum_Tolerated_Dose_Feed

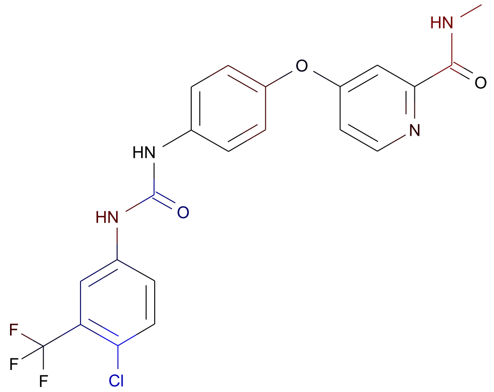

$C_{21}H_{16}ClF_3N_4O_3$
Molecular Weight: 464.82494
ALogP: 4.175
Rotatable Bonds: 6
Acceptors: 4
Donors: 3

Model Prediction

Prediction: 0.0885
Unit: g/kg_body_weight
Mahalanobis Distance: 12.4
Mahalanobis Distance p-value: 1.76e-009

Mahalanobis Distance: The Mahalanobis distance (MD) is a generalization of the Euclidean distance that accounts for correlations among the X properties. It is calculated as the distance to the center of the training data. The larger the MD, the less trustworthy the prediction.
Mahalanobis Distance p-value: The p-value gives the fraction of training data with an MD greater than or equal to the one for the given sample, assuming normally distributed data. The smaller the p-value, the less trustworthy the prediciton. For highly non-normal X properties (e.g., fingerprints), the MD p-value is wildly inaccurate.

Structural Similar Compounds

Name	FUROSEMIDE	PHENOLPHTHALEIN	DISPERSE YELLOW 3
Structure	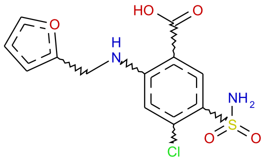	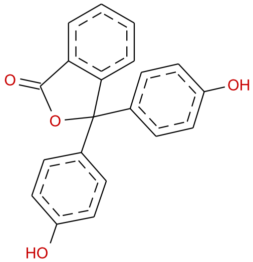	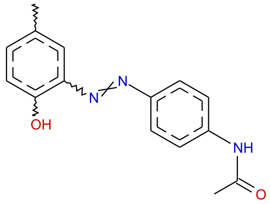
Actual Endpoint (-log C)	4.04236	2.20184	2.77703
Predicted Endpoint (-log C)	2.8614	2.8857	2.80195
Distance	0.741	0.780	0.799
Reference	NCI/NTP TR-356	NCI/NTP TR-465	NCI/NTP TR-222

Model Applicability

Unknown features are fingerprint features in the query molecule, but not found or appearing too infrequently in the training set.

1. All properties and OPS components are within expected ranges.

Feature Contribution

Top features for positive contribution

Fingerprint	Bit/Smiles	Feature Structure	Score
FCFP_2	-885550502	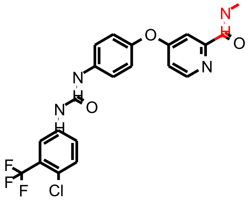 [*]CNC(=[*])[*]	0.115

FCFP_2	3	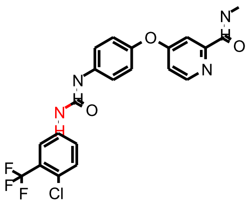 [*]N[*]	0.0737
FCFP_2	332760439	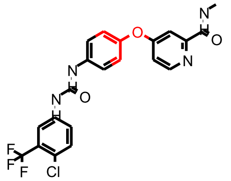 [*]O[c](:[cH]:[*]):[cH]:[*]	0.0611
**Top Features for negative contribution**			
**Fingerprint**	**Bit/Smiles**	**Feature Structure**	**Score**
FCFP_2	71476542	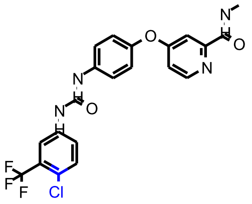 [*]:[c](:[*])Cl	-0.134
FCFP_2	1872154524	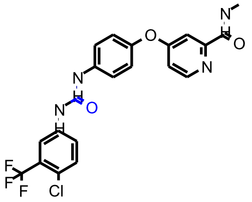 [*]C(=O)[*]	-0.105
FCFP_2	203677720	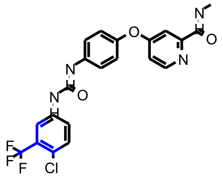 [*]C(=[*])[c](:[cH]:[*]):[cH]:[*]	-0.0829

11RR

TOPKAT_Rat_Maximum_Tolerated_Dose_Gavage

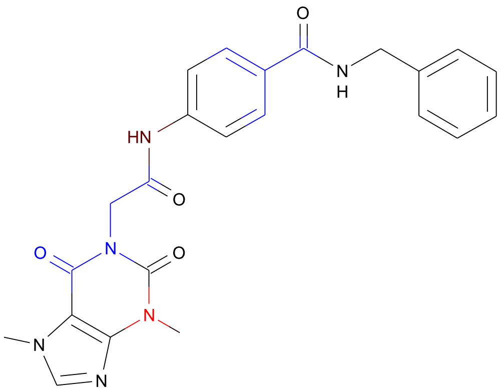

$C_{23}H_{22}N_6O_4$

Molecular Weight: 446.45858

ALogP: 1.328

Rotatable Bonds: 6

Acceptors: 5

Donors: 2

Model Prediction

Prediction: 0.232

Unit: g/kg_body_weight

Mahalanobis Distance: 11.3

Mahalanobis Distance p-value: 1.01e-007

Mahalanobis Distance: The Mahalanobis distance (MD) is a generalization of the Euclidean distance that accounts for correlations among the X properties. It is calculated as the distance to the center of the training data. The larger the MD, the less trustworthy the prediction.

Mahalanobis Distance p-value: The p-value gives the fraction of training data with an MD greater than or equal to the one for the given sample, assuming normally distributed data. The smaller the p-value, the less trustworthy the prediciton. For highly non-normal X properties (e.g., fingerprints), the MD p-value is wildly inaccurate.

Structural Similar Compounds

Name	OCHRATOXIN	SULFISOOXAZOLE	PENICILLIN VK
Structure	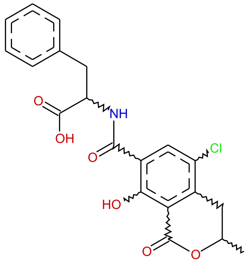	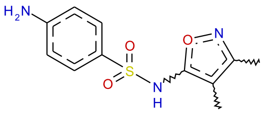	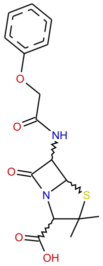
Actual Endpoint (-log C)	6.28396	2.82494	2.54455
Predicted Endpoint (-log C)	5.12358	3.0705	3.9702
Distance	0.800	0.832	0.936
Reference	NCI/NTP TR-358	NCI/NTP TR-138	NCI/NTP TR-336

Model Applicability

Unknown features are fingerprint features in the query molecule, but not found or appearing too infrequently in the training set.

1. Molecular_Weight out of range. Value: 446.46. Training min, max, mean, SD: 68.074, 434.63, 171.13, 85.06.
2. Num_AromaticRings out of range. Value: 3. Training min, max, mean, SD: 0, 2, 0.5625, 0.693.
3. Unknown FCFP_2 feature: -124685461: [*]n1:[*]:[*]:n:[cH]:1
4. Unknown FCFP_2 feature: -306856457: [*][c]1:[*]:[*]:[cH]:n:1C
5. Unknown FCFP_2 feature: -1410049896: [*]N([*])[c]1:n:[*]:[*]:[c]:1[*]
6. Unknown FCFP_2 feature: -1986158408: [*]N([*])C(=O)N([*])[*]
7. Unknown FCFP_2 feature: 136150461: [*]:n(:[*])C

Feature Contribution

Top features for positive contribution			
**Fingerprint**	**Bit/Smiles**	**Feature Structure**	**Score**

FCFP_2	1	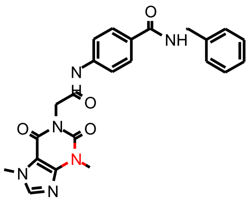 [*]N([*])[*]	0.511
FCFP_2	3	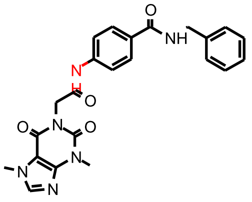 [*]N[*]	0.104
FCFP_2	136627117	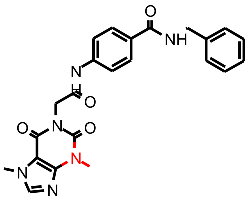 [*]N([*])C	0.0304

Top Features for negative contribution

**Fingerprint**	**Bit/Smiles**	**Feature Structure**	**Score**
FCFP_2	203677720	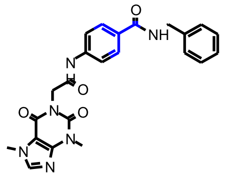 [*]C(=[*])[c](:[cH]:[*]):[cH]:[*]	-0.406
FCFP_2	1872154524	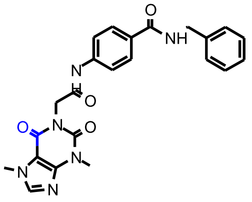 [*]C(=O)[*]	-0.307

FCFP_2	0	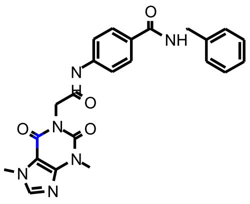<br>[*]C(=[*])[*]	-0.29

Sorafenib

TOPKAT_Rat_Maximum_Tolerated_Dose_Gavage

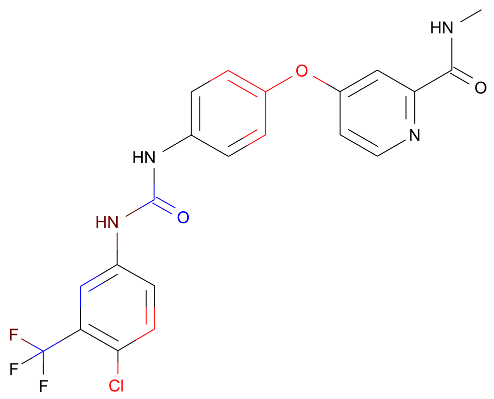

$C_{21}H_{16}ClF_3N_4O_3$

Molecular Weight: 464.82494

ALogP: 4.175

Rotatable Bonds: 6

Acceptors: 4

Donors: 3

Model Prediction

Prediction: 0.000918

Unit: g/kg_body_weight

Mahalanobis Distance: 12.2

Mahalanobis Distance p-value: 4.69e-009

Mahalanobis Distance: The Mahalanobis distance (MD) is a generalization of the Euclidean distance that accounts for correlations among the X properties. It is calculated as the distance to the center of the training data. The larger the MD, the less trustworthy the prediction.

Mahalanobis Distance p-value: The p-value gives the fraction of training data with an MD greater than or equal to the one for the given sample, assuming normally distributed data. The smaller the p-value, the less trustworthy the prediciton. For highly non-normal X properties (e.g., fingerprints), the MD p-value is wildly inaccurate.

Structural Similar Compounds

Name	OCHRATOXIN	SULFISOOXAZOLE	PENICILLIN VK
Structure	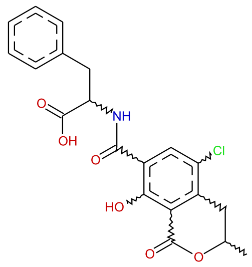	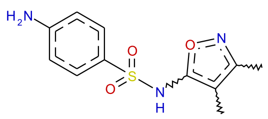	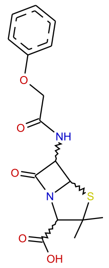
Actual Endpoint (-log C)	6.28396	2.82494	2.54455
Predicted Endpoint (-log C)	5.12358	3.0705	3.9702
Distance	0.758	0.997	1.159
Reference	NCI/NTP TR-358	NCI/NTP TR-138	NCI/NTP TR-336

Model Applicability

Unknown features are fingerprint features in the query molecule, but not found or appearing too infrequently in the training set.

1. Molecular_Weight out of range. Value: 464.82. Training min, max, mean, SD: 68.074, 434.63, 171.13, 85.06.
2. Num_AromaticRings out of range. Value: 3. Training min, max, mean, SD: 0, 2, 0.5625, 0.693.
3. OPS PC5 out of range. Value: -3.5737. Training min, max, SD, explained variance: -3.4, 4.1587, 1.489, 0.0686.
4. OPS PC7 out of range. Value: -3.8342. Training min, max, SD, explained variance: -2.8003, 2.9332, 1.16, 0.0416.
5. Unknown FCFP_2 feature: 1499521844: [*]NC(=O)N[*]
6. Unknown FCFP_2 feature: -1029533685: [*]:[c](:[*])C(F)(F)F
7. Unknown FCFP_2 feature: 136686699: [*]NC

Feature Contribution

Top features for positive contribution			
Fingerprint	Bit/Smiles	Feature Structure	Score

FCFP_2	332760439	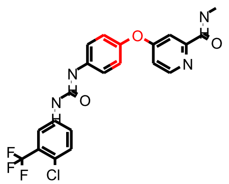<br>[*]O[c](:[cH]:[*]):[cH]:[*]	0.672
FCFP_2	32	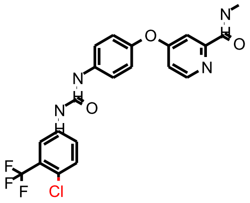<br>[*]Cl	0.526
FCFP_2	1	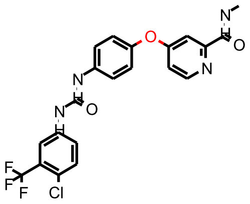<br>[*]N([*])[*]	0.511

Top Features for negative contribution

**Fingerprint**	**Bit/Smiles**	**Feature Structure**	**Score**
FCFP_2	203677720	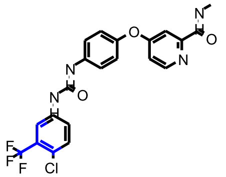<br>[*]C(=[*])[c](:[cH]:[*]):[cH]:[*]	-0.406
FCFP_2	1872154524	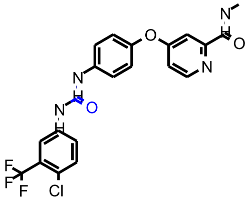<br>[*]C(=O)[*]	-0.307

FCFP_2	0	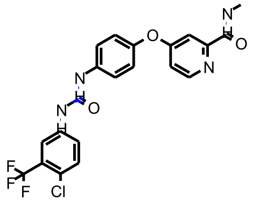 [*]C(=[*])[*]	-0.29

11RR

TOPKAT_Rat_Oral_LD50

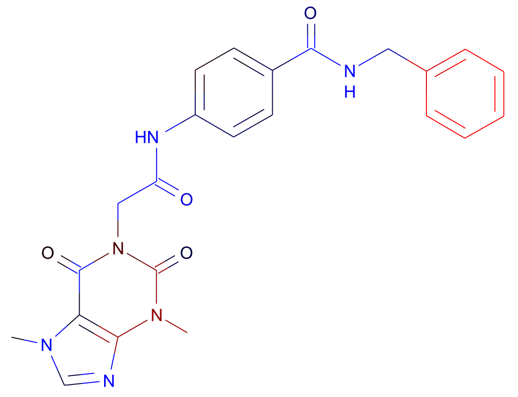

$C_{23}H_{22}N_6O_4$

Molecular Weight: 446.45858

ALogP: 1.328

Rotatable Bonds: 6

Acceptors: 5

Donors: 2

Structural Similar Compounds

Name	PRASOZIN .HCl (HCl STRIPPED)	CARBAMIC ACID; [1-[(5-CYANOPENTYL)CARBAMOYL]BENZIMIDAZOL-2-YL]-; METHYL ESTER	PIRETANIDE
Structure	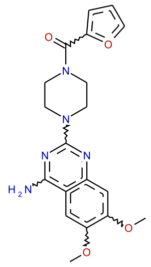	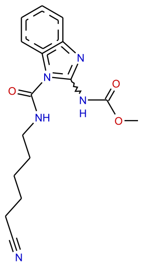	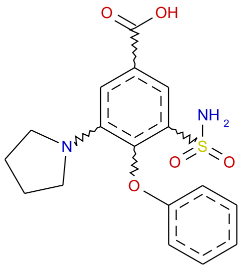
Actual Endpoint (-log C)	2.294	2.12	1.811
Predicted Endpoint (-log C)	3.00765	1.78415	1.83976
Distance	0.675	0.693	0.703
Reference	NIIRDN 6;688;82	85ARAE 4;118;76/77	DRFUD4 2;393;77

Model Prediction

Prediction: 7.25

Unit: g/kg_body_weight

Mahalanobis Distance: 20.4

Mahalanobis Distance p-value: 2.58e-010

Mahalanobis Distance: The Mahalanobis distance (MD) is a generalization of the Euclidean distance that accounts for correlations among the X properties. It is calculated as the distance to the center of the training data. The larger the MD, the less trustworthy the prediction.
Mahalanobis Distance p-value: The p-value gives the fraction of training data with an MD greater than or equal to the one for the given sample, assuming normally distributed data. The smaller the p-value, the less trustworthy the prediciton. For highly non-normal X properties (e.g., fingerprints), the MD p-value is wildly inaccurate.

Model Applicability

Unknown features are fingerprint features in the query molecule, but not found or appearing too infrequently in the training set.

1. All properties and OPS components are within expected ranges.
2. Unknown FCFP_6 feature: 16: [*]:[cH]:[*]
3. Unknown FCFP_6 feature: 1747237384: [*][c]1:[*]:[*]:[cH]:n:1
4. Unknown FCFP_6 feature: -124685461: [*]n1:[*]:[*]:n:[cH]:1
5. Unknown FCFP_6 feature: -306856457: [*][c]1:[*]:[*]:[cH]:n:1C
6. Unknown FCFP_6 feature: -1410049896: [*]N([*])[c]1:n:[*]:[*]:[c]:1[*]
7. Unknown FCFP_6 feature: 136150461: [*]:n(:[*])C
8. Unknown FCFP_6 feature: 1618154665: [*][c](:[*]):[cH]:[cH]:[*]
9. Unknown FCFP_6 feature: 907096426: [*]NC[c](:[*]):[*]

Feature Contribution

Top features for positive contribution			
**Fingerprint**	**Bit/Smiles**	**Feature Structure**	**Score**

ECFP_6	642810091	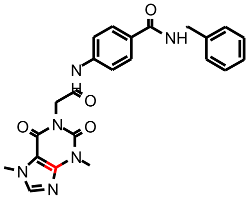 [*][c](:[*]):[*]	0.281
ECFP_6	-1897341097	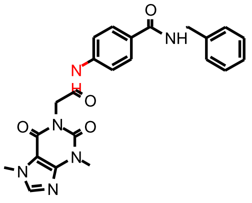 [*]N[*]	0.216
ECFP_6	1571214559	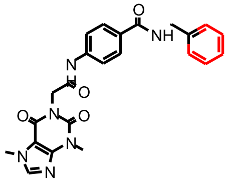 [*]1:[cH]:[cH]:[cH]:[cH]:[cH]:1	0.19

Top Features for negative contribution

Fingerprint	Bit/Smiles	Feature Structure	Score
ECFP_6	2106656448	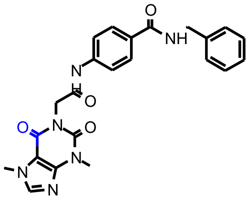 [*]C(=O)[*]	-0.352
ECFP_6	497523368	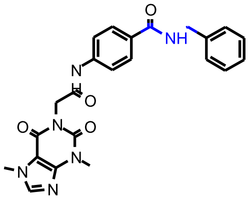 [*]CNC(=[*])[*]	-0.301

ECFP_6	655739385	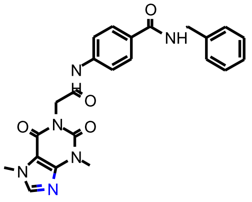<br>[*]:n:[*]	-0.239

Sorafenib

TOPKAT_Rat_Oral_LD50

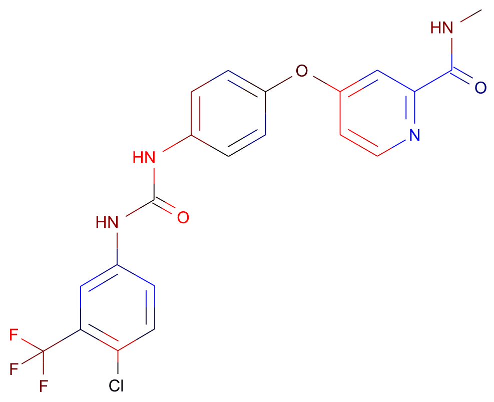

$C_{21}H_{16}ClF_3N_4O_3$

Molecular Weight: 464.82494

ALogP: 4.175

Rotatable Bonds: 6

Acceptors: 4

Donors: 3

Structural Similar Compounds

Name	FLUBENDAZOLE	PHOSPHORAMIDOTHIOIC ACID; ACETIMIDOYL-; O;O-bis-(p-CHLOROPHENYL)ESTER	BEZAFIBRATE
Structure	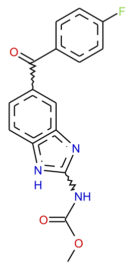	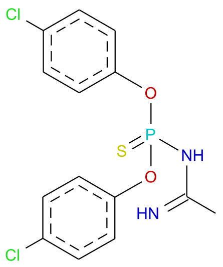	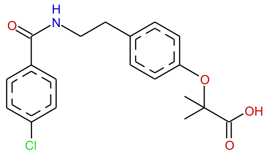
Actual Endpoint (-log C)	2.088	5.006	1.946
Predicted Endpoint (-log C)	2.69288	3.23989	2.54395
Distance	0.697	0.703	0.721
Reference	YRTMA6 9;11;78	FMCHA2 -;C149;89	ARZNAD 30;2023;80

Model Prediction

Prediction: 0.823

Unit: g/kg_body_weight

Mahalanobis Distance: 21

Mahalanobis Distance p-value: 1.93e-012

Mahalanobis Distance: The Mahalanobis distance (MD) is a generalization of the Euclidean distance that accounts for correlations among the X properties. It is calculated as the distance to the center of the training data. The larger the MD, the less trustworthy the prediction.
Mahalanobis Distance p-value: The p-value gives the fraction of training data with an MD greater than or equal to the one for the given sample, assuming normally distributed data. The smaller the p-value, the less trustworthy the prediciton. For highly non-normal X properties (e.g., fingerprints), the MD p-value is wildly inaccurate.

Model Applicability

Unknown features are fingerprint features in the query molecule, but not found or appearing too infrequently in the training set.

1. All properties and OPS components are within expected ranges.
2. Unknown FCFP_6 feature: 16: [*]:[cH]:[*]
3. Unknown FCFP_6 feature: 71476542: [*]:[c](:[*])Cl
4. Unknown FCFP_6 feature: 1747237384: [*][c]1:[*]:[*]:[cH]:n:1
5. Unknown FCFP_6 feature: 1618154665: [*][c](:[*]):[cH]:[cH]:[*]
6. Unknown FCFP_6 feature: 136686699: [*]NC

Feature Contribution

Top features for positive contribution			
**Fingerprint**	**Bit/Smiles**	**Feature Structure**	**Score**

FCFP_6	71953198	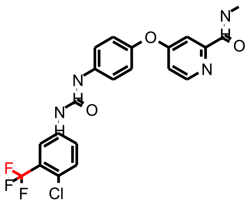 [*]C([*])([*])F	0.392
ECFP_6	-1046436026	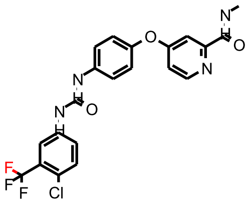 [*]F	0.349
ECFP_6	642810091	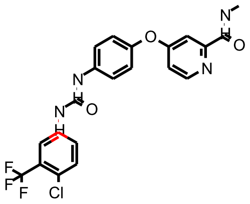 [*][c](:[*]):[*]	0.281

Top Features for negative contribution

**Fingerprint**	**Bit/Smiles**	**Feature Structure**	**Score**
ECFP_6	226796801	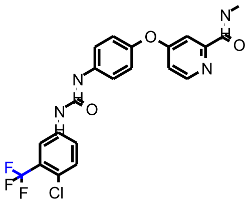 [*]C([*])([*])F	-0.32
ECFP_6	-817402818	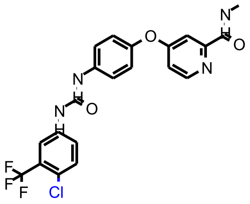 [*]Cl	-0.263

ECFP_6	-176455838	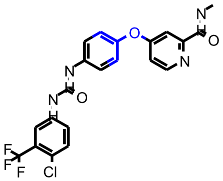 [*]O[c](:[cH]:[*]):[cH]:[*]	-0.257
